# Multidisciplinary guidelines on renal replacement therapy in intensive care medicine

**DOI:** 10.1186/s13054-025-05817-6

**Published:** 2026-01-14

**Authors:** Melanie Meersch-Dini, Mariam Abu-Tair, Matthias Bayer, Alexander Brinkmann, Romuald Bellmann, Frank Brunkhorst, Florian Custodis, David Czock, Otto Frey, Jan Galle, Carsten Hermes, Michael Joannidis, Stefan John, Achim Jörres, Thomas Kerz, Detlef Kindgen-Milles, Martin Koczor, Rainer Kram, Martin Kuhlmann, Michael Oppert, Georg Schlieper, Michael Schmitz, Alexander Zarbock, Carsten Willam

**Affiliations:** 1 Department of Anesthesiology, Intensive Care and Pain Medicine, University Hospital Münster, Münster, Germany; 2 Department of Nephrology and Diabetology, Evangelisches Klinikum Bethel, Bielefeld, Germany; 3 Asklepios Klinik Lich GmbH, Lich, Germany; 4 Department of Anesthesia and Critical Care Medicine, General Hospital of Heidenheim, Heidenheim, Germany; 5 Division of Medical Intensive Care and Emergency Medicine, Clinical Pharmacokinetics Unit, Department of Internal Medicine I, Medical University of Innsbruck, Innsbruck, Austria; 6 Institute of Infectious Diseases and Infection Control, Jena University Hospital, Jena, Germany; 7 Department of Internal Medicine II, Klinikum Saarbrücken, Saarbrücken, Germany; 8 Internal Medicine IX, Department of Clinical Pharmacology and Pharmacoepidemiology, Heidelberg University Hospital, Heidelberg, Germany; 9 Department of Pharmacy, General Hospital of Heidenheim, Heidenheim, Germany; 10 Märkische Kliniken GmbH, Klinikum Lüdenscheid, Lüdenscheid, Germany; 11 University of Applied Sciences Hamburg, Hamburg, Germany; 12 Division of Intensive Care and Emergency Medicine, Department of Internal Medicine, Medical University of Innsbruck, Innsbruck, Austria; 13 Department of Internal Medicine, Klinikum Nürnberg, Nürnberg, Germany; 14 Department of Medicine I-Nephrology, Transplantation & Medical Intensive Care, Medical Center Cologne-Merheim, University Witten/Herdecke, Cologne, Germany; 15 Department of Neurosurgery, University Medical Center Mainz, Mainz, Germany; 16 Department of Anesthesiology, University Hospital Düsseldorf, Heinrich Heine University, Düsseldorf, Germany; 17 Bundesverband Niere e.V, Mainz, Germany; 18 Department of Internal Medicine - Nephrology, Vivantes Klinikum im Friedrichshain, Berlin, Germany; 19 Department of Emergency and Intensive Care Medicine, Klinikum Ernst von Bergmann Klinikum, Potsdam, Germany; 20 Health and Medical University Potsdam, Potsdam, Germany; 21 Center for Nephrology, Hypertension and Metabolic Diseases, Hannover, Germany; 22 Department of Nephrology, Städtisches Klinikum Solingen, Solingen, Germany; 23 Department of Nephrology and Hypertension, Friedrich-Alexander-University Erlangen-Nürnberg, University Hospital Erlangen, Erlangen, Germany; 24 Department of Anaesthesiology, Intensive Care and Pain Medicine, University of Muenster, Albert-Schweitzer-Campus 1, Building A1, 48149 Muenster, Germany

**Keywords:** Renal replacement therapy, Guidelines, ICU, Acute kidney injury, Adults, Pharmacotherapy

## Abstract

**Background:**

Renal replacement therapy (RRT) is frequently used in critically ill patients with acute kidney injury (AKI). Here, we provide guidelines for the management of RRT in critically ill patients on the intensive care unit (ICU).

**Methods:**

We convened a systemic literature research and a Delphi process with a bi-national multidisciplinary consensus panel including 22 clinicians of 12 different German-speaking societies (Germany and Austria) with expertise in RRT. This structured guideline process was the basis for the evidence-based statements and recommendations.

**Results:**

We identified seven clinical areas needing guidance: (1) start, (2) modality (diffusion and convection), (3) continuous/ intermittent, (4) anticoagulation, (5) dose (6) pharmacotherapy, (7) stopping criteria. The consensus produced 73 statements and recommendations regarding key clinical areas, the most important 47 statements and recommendations are summarized in this overview.

**Conclusions:**

This evidence-based bi-national guideline should provide physicians with guidance for delivering best practice to critically ill patients with a dialysis-dependent AKI.

**Supplementary Information:**

The online version contains supplementary material available at 10.1186/s13054-025-05817-6.

## Introduction

Renal replacement therapy (RRT) is one of the most frequently used techniques for supporting critically ill patients with acute kidney injury (AKI) in the intensive care unit (ICU). In this specific setting, AKI occurs in one of two patients with 24% requiring RRT [1]. In 2012, the *Kidney Disease: Improving Global Outcomes* (KDIGO) group developed a guideline on AKI that included RRT management [2]. In 2018, the *National Institute for Health and Care Excellence* (NICE) guidelines followed (NG107). Since then, numerous studies have been published, providing new insights into RRT treatment strategies for critically ill patients with AKI.

To update clinical guidelines for RRT in intensive care, a national multidisciplinary and multiprofessional panel of experts specialized in critical care, nephrology, anesthesiology, and other RRT-related fields was assembled to provide clinical practice consensus recommendations based on the highest level of international evidence. Patient organizations including patients with lived experience (PWLE) were also involved. We identified seven key topics and, based on a targeted literature review, developed corresponding recommendations based on available evidence.

Although this guideline was developed under the auspices of German and Austrian national medical societies, its scope is not limited to Germany/Austria. The underlying evidence was deliberately derived from international literature, and the recommendations follow widely accepted methodological standards. The statements and guidance are intended to be broadly applicable and useful to clinicians in any healthcare setting, not only within Germany/Austria.

## Methods

### Consensus guideline members

A national multidisciplinary and multiprofessional consensus panel of 22 experts selected by 10 different national societies and 2 patient organizations was assembled. The panel included anesthesiologists, intensivists, nephrologists, internists, surgeons, and other professionals in intensive care medicine with expertise or involvement in RRT. Each participant had to disclose their conflicts of interest before the voting process began, and the permissions of each participant in the voting process were transparently defined in advance (https://register.awmf.org/de/leitlinien/detail/040-017). Overall, seven different topics were identified by the complete group and scientific questions were formulated according to the PICO (P: Population, I: Intervention, C: Comparison, and O: Outcome) framework [3]. The individual topics were then [1] developed in break-out groups by means of a systematic literature analysis [2], agreed in advance in a DELPHI procedure, and [3] discussed and agreed in consensus conferences using the AGREE II criteria (according to the guidelines of the *Working Group of the Scientific Medical Societies* (AWMF)). Pubmed, Scopus, and Cochrane were designated as literature databases.

### Guideline development

Two leaders were nominated for each group for leading and cross group coordination. In each group, articles were selected and reviewed by two experts, a third expert performed an additional manual search. The groups summarized the findings and developed guidelines for each subsection. The consensus guideline members met regularly throughout the year in subgroups and whole-group settings to discuss their progress and reach a consensus on the finalized document.

A modified Delphi process was performed. The structured consensus was reached as part of a nominal group process and structured consensus conference under independent moderation of the AWMF. All recommendations were discussed in a total of 9 complete-group consensus conferences and agreed under the leadership of the AWMF. Following the GRADE approach, the guideline panel rated the certainty of evidence (Table 1) for each critical outcome and determined the strength of each recommendation based on the certainty of evidence (Table 2), the balance between benefits and harms, patient values and preferences, and resource use. Determination of consensus strength was defined as strong (> 95% of panelists), moderate (> 75–95% of panelists), majority consent (> 50–75% of panel-ists), or no consent (< 50% of panelists). Additionally, three independent experts peer reviewed the recommendations before finalization. The complete evidence report can be found in the Supplementary Material (Supplementary Material – Evidence Report).

**Table 1:** Certainty of evidence

Symbol	Quality rating	Interpretation
⊕⊕⊕⊕	High	We have high certainty that the true effect is close to the estimated effect
⊕⊕⊕⊝	Moderate	We have moderate confidence in the effect estimate: the true effect is likely to be close to the estimate, but there is a possibility that it is substantially different
⊕⊕⊝⊝	Low	Our confidence in the effect estimate is limited: the true effect may be substantially different from the estimate
⊕⊝⊝⊝	Very low	We have very little confidence in the effect estimate: the true effect is likely to be substantially different from the estimate
		Expert opinion

The guideline and recommendations are summarized and presented in 7 sections: (1) start, (2) diffusion and convection, (3) continuous/intermittent, (4) anticoagulation, (5) dose (6) pharmacotherapy, (7) stopping criteria. The consensus produced 73 statements and recommendations in total regarding key clinical areas (Supplementary Material – Further Recommendations). The most important 47 statements and recommendations are summarized in this overview.

## Results

1. **Start of Renal Replacement Therapy**

**Table:** 

1.1- We recommend to start RRT immediately in case of life-threatening changes in fluid, acid-base or electrolyte balance	Expert consensus
1.2- If RRT is expected to become necessary in AKI based on the clinical condition, course of illness and/or pre-existing diseases, it should be initiated without further delay	B ⊕⊕⊝⊝
1.3- We suggest that in cases of non-life-threatening changes, or when the need for RRT is uncertain, conservative measures should be implemented to avoid initiating RRT, with regular re-evaluation of the patient’s condition	B ⊕⊕⊝⊝
1.4- RRT should not be initiated solely for the purpose of enhancing renal recovery	B ⊕⊕⊝⊝
1.5- In patients with isolated elevations in serum urea or creatinine without clinical signs or symptoms attributable to kidney failure, initiation of RRT may be deferred	0 ⊕⊝⊝⊝
1.6- We suggest that a negative furosemide stress test alone should not prompt the initiation of RRT	B ⊕⊕⊝⊝
1.7- To date, biomarkers alone cannot be recommended for the decision to start RRT	Expert consensus

The initiation of RRT in case of life-threatening complications (hypervolemia, hyperkalemia, acidosis, uremia) is largely undisputed, despite the lack of evidence due to ethical reasons (Table 3) (*recommendation 1.1*). However, when relative indications occur, it is unclear whether an early start of treatment, aimed at preventing life-threatening complications and ensuring a more stable metabolic status, may lead to improved outcomes.

**Table 2:** Grade of recommendations

Grade of recommendation	Description
A	Strong recommendation
B	Weak/conditional recommendation
0	No recommendation or insufficient evidence

**Table 3:** Absolute indications for renal replacement therapy

Absolute indicators	Standard values	Notes
Hyperkalaemia	> 6.0 mmol/l	Therapy-resistant
Acidosis	pH < 7.2	Therapy-resistant and caused by AKI
Volume overload	Edema, pulmonary venous congestion	Therapy-resistant
Uremia	Clinical presentation	Among others disorders of consciousness

### Mortality

Current evidence suggests that the KDIGO criteria alone are not sufficient for guiding RRT initiation [4–7]. A Cochrane analysis by Fayad et al. [8], updated in 2022, compared mortality outcomes between early and late initiation of RRT in critically ill patients [9]. The review found that early initiation had little to no effect on 30-day mortality (12 studies, 4,826 participants: RR 0.97, 95% CI 0.87 to 1.09; I² = 29%; low-certainty evidence) or on mortality beyond 30 days (7 studies, 4,534 participants: RR 0.99, 95% CI 0.92 to 1.07; I² = 6%; moderate-certainty evidence) [9]. However, a critical limitation of RCTs and systematic reviews is that a significant proportion of patients in the “late” arms never received RRT. Notably, in the largest RCT to date by Investigators [4] only 61.8% of patients in the delayed group ultimately received RRT [4]. Based on the fact that there is some evidence showing that an early start (at KDIGO stage 2) is associated with improved survival in patients with high illness severity [7] while a too late start (KDIGO stage 3, oliguria for > 72 h or BUN > 112 mg/dl, and mandatory indication (noticeable hyperkalemia or metabolic acidosis or pulmonary edema) or until blood urea nitrogen con-centration reached 140 mg/dL) may increase mortality [10], as well as the fact that different studies have shown that clinical assessment is an important aspect [4, 11], the consensus group recommends that RRT initiation should consider the clinical situation, the course of the disease and underlying comorbidities rather than single laboratory values. (*recommendations 1.2*,* 1.3*)

### Renal recovery

Three reviews defined renal recovery as independence from RRT, while five others used different definitions. The results across studies demonstrated substantial hetero-geneity (I² = 22–70%), and no systematic review found a consistent benefit of early RRT ini-tiation on renal recovery. STARRT-AKI found early RRT to be associated with higher RRT de-pendency at day 90 (10.4% vs. 6.0%; RR 1.74 (95% CI, 1.24–2.43) although renal recovery (eGFR reduction of > 25% from baseline at 90 days) was not different [4]. A secondary analysis of the STARRT-AKI trial suggested that early RRT initiation may also impair renal recovery in patients with pre-existing CKD [12]. However, a separate analysis of the STARRT-AKI trial which included both CRRT and IHD indicated that CRRT was associated with a higher likelihood of renal recovery (OR 0.81; 95% CI 0.66–0.99) [13]. (*recommendation 1.4*)

### Relative indications for RRT

There has been a notable paradigm shift in the literature emphasizing the patient’s overall clinical condition instead of relying on isolated surrogate markers, such as urea levels or oliguria. This shift reflects a more precise, individualized approach to determining the appropriate timing for RRT. The systematic review by Karvellas et al. [14] analyzed 6 RCTs that used elevated urea levels as initiation criteria, 3 that focused on reduced urine output, 3 on elevated creatinine, one on hyperkalemia, and only 2 that employed simple time-based criteria (e.g., 24–48 h of anuria) [14]. In contrast, more recent large RCTs have adopted time-based initiation strategies, typically between 12 and 48 h after AKI diagnosis, while incorporating the presence or absence of clinical complications [4, 7, 15, 16]. The Cochrane review by Fayad et al. [9] likewise focused on RCTs that used such time-based criteria, rather than relying solely on biochemical surrogates [9]. (*recommendation 1.5*)

### Furosemide stress test (FST)

The test involves administering a furosemide bolus of 1 mg/kg in furosemide-naïve patients (1.5 mg/kg in those previously treated with furosemide) [17]. A urine output of less than 200 mL within the subsequent two hours is considered a negative result, indicating severe and progressive tubular injury.

A systematic review by Chen et al. [18], which included 11 studies (non-RCTs) with a total of 1,366 patients, evaluated the predictive value of the FST for severe AKI progression and the need for RRT [18]. The results showed strong predictive power for AKI progression: OR 29.7 (95% CI 17.0–51.6) and RRT: OR 13.6 (95% CI 5.74–32.17). Based on current findings, the FST may be recommended as a non-invasive tool to assess the likelihood of AKI progression. However, a negative FST (poor diuretic response) suggests a high probability of AKI progression but does not automatically indicate the need for RRT. Conversely, a positive FST indicates preserved tubular function, making the development of progressive AKI less likely. (*recommendation 1.6*)

### Biomarkers

Only two initiation RCTs incorporated novel renal biomarkers with no clear benefit [7, 19]. More recently, Meersch et al. [20] showed that combining functional testing (FST) with structural biomarkers significantly improved the prediction of absolute indications for initiating RRT [20]. While the integration of biomarkers into RRT decision-making is an area of active research, the current evidence is insufficient to support a formal recommendation for or against their routine clinical use. (*recommendation 1.7*)

2. **Diffusion and convection in Renal Replacement Therapy**

**Table:** 

2.1- We recommend that in patients with AKI and an indication for RRT, diffusive, convective, or a combination of them produce similar outcomes	A ⊕⊝⊝⊝
2.2- In hemofiltration, the use of pre- or post-dilution can equally be considered; however, pre-dilution may be preferred in cases of repeated filter clotting	0 ⊕⊝⊝⊝
2.3- We recommend that for patients with sepsis requiring RRT in the ICU, diffusive, convective, or combined modalities can be used equally	A ⊕⊝⊝⊝
2.4- We recommend that in cases of severe, life-threatening hyperkalemia, a diffusive procedure with high dialysate flow rates should be preferred over a convective procedure, if available	Expert consensus (strong)
2.5- We recommend that RRT for rhabdomyolysis should be initiated only in the presence of AKI with a clear indication for RRT	A ⊕⊝⊝⊝

Current literature does not provide evidence that either diffusion or convection is superior in critically ill patients with AKI. Both methods provide effective metabolic control. Two small RCTs (*n* = 60 and *n* = 161) showed longer filter lifespans with diffusive techniques compared to pure convection [21, 22]. The systematic review by Côté et al. [23] including 615 patients of clinical trials and observational studies receiving intermittent modalities found no difference in mortality, renal recovery, or hemodynamic stability between the two methods [23]. (*recommendation 2.1*) However, convective methods may have shorter filter lifespans due to hemoconcentration and increased clotting risk even when citrate anticoagulation is used [24]. 

### Pre- and postdilution in convection

Predilution requires a significantly higher substitution volume to achieve the same clearance. In contrast, postdilution offers greater dialysis efficiency with identical substitution volumes. Uchino et al. [25] reported significantly longer filter life with predilution (18 vs. 13 h; *p* = 0.021) [25], while Nurmohamed et al. [26] found only a non-significant trend toward longer filter life (29 ± 46 vs. 24 ± 38 h; *p* = 0.58) [26]. Despite a 19% higher CRRT dose with postdilution in the study by Nurmohamed (26.7 ± 9.6 vs. 22.4 ± 4.4 ml/kg/h; *p* = 0.01), neither study demonstrated a clear advantage in controlling uremia, as measured by creatinine and urea levels. (*recommendation 2.2*)

### Diffusion and convection in sepsis

In theory, convective procedures offer advantages for patients with sepsis because they provide a higher clearance of middle molecules, which way pro-inflammatory mediators may be cleared more efficiently [27]. By using high-cut-off membranes, the elimination of larger molecules is also possible. However, unselective elimination of proteins may not be beneficial. Systematic reviews of septic AKI patients by Zha et al. [28] and Snow et al. [29] found no significant differences in mortality or other outcomes when comparing diffusion and convection techniques. A RCT by Jang et al. [30] with 96 septic AKI patients found no differences in 7-, 28- or 90-day mortality, SOFA scores, or reductions in nitrogen, creatinine or ß2-microglobulin levels between continuous hemodialysis and continuous hemodiafiltration [30]. (*recommendation 2.3*)

### Diffusion and convection to treat hyperkalemia

In a systematic review, Côté et al. [23] compared diffusion and convection in intermittent procedures and found no differences in mortality, renal recovery, or hemodynamic instability [23]. The flux of potassium across dialysis membranes depends primarily on the potassium concentration gradient in the dialysate, the size of the dialysis membrane surface, and the blood and dialysate flow rates. Diffusive procedures allow a significantly higher dialysate fluid turnover compared to substitution fluid turnover in convective procedures, because the latter are limited by hemoconcentration. Consequently, diffusion is more efficient at removing small molecules, especially when high dialysate flow rates are applied. In continuous procedures, potassium decreases more slowly due to lower dialysis doses per hour, and thus gradually approaching the dialysate concentration. In combined methods (e.g., hemodiafiltration), the diffusive component plays a crucial role in potassium elimination when used in a higher dose [31]. *(recommendation 2.4*)

### Rhabdomyolysis

Severe rhabdomyolysis can lead to high serum myoglobin levels, which may cause tubular obstruction and AKI. Myoglobin (17.8 kDa) is freely filtered in the glomerulus but poorly cleared by standard dialysis. No clear myoglobin threshold exists to guide RRT initiation. Convective techniques (e.g., hemofiltration, hemodiafiltration, high cut-off filters) have shown improved myoglobin clearance in small studies [32], but without consistent evidence of better outcomes [33, 34]. However, RRT may be necessary in cases of oliguria or anuria with suspected tubular obstruction to prevent life-threatening complications of acute kidney injury (AKI). (*recommendation 2.5*)

3. **Continuous and intermittent Renal Replacement Therapy**

**Table:** 

3.1- Continuous or intermittent RRT modality can be used equally to ensure patient survival in severe AKI	0 ⊕⊝⊝⊝
3.2- We recommend that when selecting the RRT modality the individual clinical situation of the patient be considered, which may justify a preference for either continuous or intermittent therapy	Expert consensus (strong)
3.3- We suggest that in hemodynamically unstable patients with AKI requiring dialysis, continuous or prolonged RRT modalities be preferred to ensure hemodynamic stability and reduce hypotension	B ⊕⊝⊝⊝
3.4- Intermittent or continuous procedures can be equally considered for patients with thrombocytopenia	0 ⊕⊝⊝⊝
3.5- We recommend that patients with increased intracranial pressure be treated with RRT adapted to maintain appropriate serum osmolality. Serum osmolality, sodium, urea, and blood glucose should be regularly monitored using validated laboratory methods to prevent disequilibrium syndrome	Expert consensus
3.6- We recommend using intermittent, prolonged or continuous modalities as they all have the potential to achieve a negative fluid balance in cases of fluid overload	Expert consensus
3.7- We recommend that early mobilization should not be prevented or delayed by the use of continuous RRT	A ⊕⊝⊝⊝

CRRT provides continuous 24-hour treatment, while IHD runs 4–6 h per session, and PIRRT typically lasts 6–12 h. Continuous methods offer stable solute and fluid removal, whereas intermittent therapies require higher doses over shorter periods, causing greater shifts in serum osmolality and fluid balance.

### Mortality

In the Cochrane analysis by Rabindranath et al. [35], which included 15 RCTs with 1,550 patients, a RR of 1.01 (95% CI: 0.92–1.12) was reported when comparing mortality of CRRT to IHD [35]. The most recent systematic review by Ye et al. [36], incorporating 30 RCTs and 3,774 patients, found a similar RR of 1.04 (95% CI: 0.93–1.18) [36]. These reviews, limited to RCTs, were unable to demonstrate a mortality difference between CRRT and IHD.

Six systematic reviews have assessed mortality outcomes comparing PIRRT to CRRT, with mixed results. Four reviews found no significant difference, while two suggested a slight survival benefit for PIRRT: Zhang et al. [37] a RR of 0.86 (95% CI: 0.74–1.00) [37], and Kovacs et al. [38] a RR of 1.21 (95% CI: 1.02–1.43; I² = 47%, *p* = 0.03) [38]. Overall, the available data remain insufficient and too heterogeneous to support a definitive conclusion regarding mortality differences between RRT modalities (*recommendation 3.1*).

### Renal recovery

The impact of IHD and CRRT on renal recovery after AKI has been studied in 10 systematic reviews between 2002 and 2021. Reviews based solely on RCTs found no significant difference in renal recovery or dialysis dependence. However, 4 out of 10 reviews including observational and retrospective data reported a moderate benefit for CRRT, supported by a substantially larger sample size. For instance, Schoenfelder et al. [39] analyzed 1,870 patients from RCTs (no difference) and 15,689 from non-randomized studies, favoring CRRT [39]. Notably, large post hoc analyses of the ATN, RENAL, and STARRT-AKI trials were not included in these reviews [4–6]. A secondary analysis of ATN and RENAL (*n* = 2,542) found no mortality difference but showed improved renal recovery with CRRT in the ATN cohort [40]. This finding is limited since an increased cardiovascular SOFA score automatically led to allocation to the CRRT group. Ultimately, 85.5% of patients were in the CRRT group because there was no randomization for continuous or intermittent procedures. Similarly, a post-hoc analysis of the AKIKI 1 and IDEAL-ICU trials found no difference on re-nal recovery between both techniques although a non-significant improved survival could be demonstrated for CRRT in patients with higher illness severity [41]. 

A post hoc analysis of the STARRT-AKI trial (*n* = 2,196) showed improved renal recovery with CRRT at 90 days (OR 0.61; 95% CI 0.39–0.94), but no mortality difference [13]. However, modality assignment was not randomized and was subject to center preference, with significant group imbalance (1,590 CRRT vs. 606 IHD).

French data involving 25,750 cases showed better recovery with CRRT (OR 0.91; 95% CI 0.83–0.99) [42], while the OUTCOMEREA study found no significant difference in a combined outcome of mortality and renal recovery [43]. A recent insurance-based analysis found lower dialysis dependence at 90 days with CRRT (4.9% vs. 7.4%; OR 0.68; *p* = 0.03), though patient characteristics between groups differed significantly [44].

In summary, controlled trials show no difference in renal recovery between modalities, but large observational studies suggest potential advantages for CRRT. (*recommendation 3.2*)

### Effect on hemodynamics

Hemodynamic instability can be considered both an outcome parameter and an indication criterion. As an *outcome parameter*, hemodynamic instability includes hypotension, vasopressor use, and declines in mean arterial pressure (MAP). A Canadian study identified new-onset instability as a primary reason for transitioning to CRRT [45]. Regional differences can be observed when used as an indication criterion: CRRT is preferred for unstable patients at many German and Austrian centers [46], while IHD is for instance more commonly used in France, even for unstable patients [42, 43].

A Cochrane review of 15 RCTs (*n* = 1,550) found no significant difference between CRRT and IHD in rates of hemodynamic instability or hypotension, although CRRT was associated with higher post-treatment MAP and reduced vasopressor escalation [35]. A systematic review of 49 studies including RCTs, observational and cost-effectiveness studies similarly found no significant differences (hypotensive episodes RR 0.71 (95% CI 0.39–1.31), hemodynamic instability RR 0.48 (95% CI 0.10–2.28)) [39]. More recently, a review analyzed 12 RCTs (*n* = 1,419) and reported mixed results with high heterogeneity and risk of bias [47].

While evidence is limited, CRRT may be associated with fewer hypotensive events and reduced vasopressor needs. Therefore, we recommend CRRT or prolonged therapies for patients with significant hemodynamic instability, despite the low level of supporting evidence. (*recommendation 3.3*).

### Effect on thrombocytopenia and need for transfusion

In a retrospective study of 541 ICU patients, 65% had pre-existing thrombocytopenia before CRRT while 20% developed it after initiation. Platelet counts below 50,000/µL were linked to higher mortality [48]. A secondary analysis of the RENAL trial found that both lower platelet nadir and a > 50% drop in platelet count were associated with fewer RRT-free days (OR 0.94, 95% CI 0.90–0.97; and OR 0.91, 95% CI 0.88–0.95, respectively) [49].

Transfusions may result from bleeding or frequent circuit clotting. In a prospective RCT, bleeding-related mortality with RRT was 3.6% [50]. In the largest RCT (*n* = 360), thrombocytopenia (< 50,000/µL) occurred in 18% of CRRT vs. 12% of IHD patients (*p* = 0.12) [51]. Schwenger et al. [52] found that CRRT patients received a higher transfused volume compared to PIRRT (1,976 mL vs. 1,375 mL; *p* < 0.019) [52]. A secondary analysis of the COVINT study reported no significant differences between CRRT and IHD in platelet drop or transfusion needs [50]. However, circuit clotting without re-transfusion occurred more frequently in CRRT compared to IHD (57.4% vs. 30.4%; *p* < 0.01), without clear impact on transfusion volume. Due to limited and heterogeneous data, no definitive conclusions can be drawn. Importantly, none of these studies utilized regional citrate anticoagulation, which is now widely used and may influence these outcomes (*recommendation 3.4*).

### Effect on intracranial pressure

Intermittent RRT is highly efficient but can cause rapid shifts in osmolality, electrolytes, and fluid balance. In a large retrospective study, patients with AKI and either stroke or intracerebral hemorrhage (ICH) who received RRT had higher mortality than those without RRT (stroke: OR 1.30, 95% CI 1.12–1.48; ICH: OR 1.95, 95% CI 1.61–2.36) [53]. Rapid osmolality changes, particularly in patients with a compromised blood–brain barrier, can elevate intracranial pressure, worsening neurological outcomes. Lund et al. [54] reported a median intracranial pressure (ICP) increase from 11.9 mmHg to 21 mmHg during RRT [54]. Although the extent of the rise was similar across modalities, it occurred significantly earlier with intermittent therapy (75 vs. 375 min; *p* < 0.05). Parsons et al. [55] reviewed 11 studies (*n* = 58 neurosurgical patients) and found ICP increases were more frequent with IHD (73% vs. 37.5%, *p* = 0.01), which was also associated with higher mortality (75% vs. 39.1%, *p* = 0.01) [55]. 

A literature search identified one RCT, five observational, and nine retrospective studies addressing neurological complications during RRT. These included heterogeneous populations with conditions such as hepatic encephalopathy, traumatic brain injury (TBI), and stroke. Davenport et al. [56] compared continuous arteriovenous RRT with IHD [56] and observed increases of ICP in IHD, while Wu et al. [57] found no difference in ICP between PIRRT and CRRT [57]. Similarly, Johansen et al. [58] reported minimal differences in brain volume changes between diffusion and convection-based dialysis in chronic patients [58].

The consensus group concluded that disequilibrium risk is not determined solely by modality but by the rate of osmotic shifts. Therefore, RRT prescriptions should be individualized, considering treatment intensity, filter performance, duration, and modality, along with adjunctive measures to manage sodium and glucose (*recommendation 3.5*).

### Effects on fluid status

A 2020 systematic review of 34 observational studies (31,076 patients) confirmed that fluid overload significantly increased mortality in AKI (RR 2.38, 95% CI 1.75–2.98) and post-surgical patients (RR 6.17, 95% CI 4.81–7.97) [59]. RCTs comparing IHD and CRRT showed no significant differences in ultrafiltration in five out of six studies; only Augustine et al. reported higher rates with CRRT, though normovolemia was not assessed [60]. In the PICARD study, persistent fluid overload was associated with higher 30-day mortality (OR 2.52, 95% CI 1.55–4.08) [61]. CRRT achieved lower residual fluid overload (8%) compared to IHD (18%). Similarly, Mehta et al. [62] found ultrafiltration targets were not met in 9% of CRRT and 28.8% of IHD cases [62].

Five studies comparing PIRRT and CRRT showed no difference in ultrafiltration. Albino et al. [63] found similar fluid balance with 6 h and 10 h PIRRT treatments [63]. Three systematic reviews also found no significant difference between PIRRT and CRRT in fluid removal [37, 64, 65].

Overall, all modalities can effectively manage fluid overload. The consensus group emphasizes that success depends less on modality and more on tailoring ultrafiltration to the patient’s clinical condition (*recommendation 3.6*).

### Effect on patient mobilization

Evidence from available metaanalyses show that early mobilization is associated with improved outcomes [66–68]. A recent guideline recommended to start mobilization within 72 h of ICU admission [69]. Early mobilization has been shown to improve patient-centered outcomes [68]. Concerns about catheter dislodgement can reduce adherence to mobilization protocols [69], but guidelines state that also femoral catheters are not a contraindication [70]. While intermittent modalities offer dialysis-free windows that may facilitate mobilization, studies confirm that safe mobilization is also feasible during CRRT [71–73]. Implementation of a specialized nursing quality control team for CRRT and interprofessional training led to fewer unplanned events, improved adherence to treatment duration, longer filter life, fewer infections, and reduced costs, along with higher patient satisfaction [74–77] (*recommendation 3.7*).

4. **Anticoagulation**

**Table:** 

4.1- We suggest that regional citrate and systemic heparin anticoagulation be used equally, as they do not differ in terms of patient outcomes, including mortality, renal recovery, and transfusion frequency	B ⊕⊕⊕⊝
4.2- We suggest that regional citrate anticoagulation be preferred in patients for whom heparin is contraindicated (e.g., due to bleeding) and in those with an increased risk of bleeding	B ⊕⊕⊝⊝
4.3- We suggest that if systemic heparin anticoagulation leads to shortened filter lifetimes and the treatment cannot be delivered to the desired extent, a switch to regional citrate anticoagulation should be considered	B ⊕⊝⊝⊝
4.4- In patients with shock or liver failure, regional citrate anticoagulation is not an absolute contraindication. Regional citrate anticoagulation can be performed under regular monitoring of lactate and ionized and total calcium	0 ⊕⊕⊝⊝
4.5- In cases of citrate accumulation, we recommend to switch to CRRT procedures with no or heparin anticoagulation and bicarbonate as the buffering substance	A ⊕⊕⊝⊝
4.6- We suggest that regional citrate anticoagulation be avoided, if possible, in cases of pronounced, progressive lactic acidosis associated with shock and severe liver failure	B ⊕⊕⊝⊝
4.7- Low-molecular-weight and unfractionated heparins may be considered equally for systemic anticoagulation; however, unfractionated heparin is preferred due to simpler monitoring and a shorter half-life	0 ⊕⊝⊝⊝
4.8- In patients with acute heparin-induced thrombocytopenia type II, argatroban alone may be considered for effective anticoagulation during RRT	0 ⊕⊝⊝⊝

### Regional citrate anticoagulation (RCA)

Citrate effectively inhibits coagulation when ionized calcium is reduced to ~0.3 mmol/L within the circuit [78, 79]. RCTs have not consistently shown a mortality benefit, with the exception of Oudemans-van Straaten et al. [80], who reported reduced mortality compared to Nadroparin (45% vs. 62%, *p* = 0.03) [80]. Recent systematic reviews and meta-analyses confirmed no significant effect on mortality or renal recovery [81–85] (*recommendations 4.1*,* 4.2*).

Citrate is associated with significantly longer filter life spans compared to systemic heparin [83, 84] although the prolonged circuit life may contribute to higher infection rates [86] (*recommendation 4.3*). All RCTs consistently reported fewer bleeding complications with citrate. Despite a significant reduction in bleeding events (RR 0.32; 95% CI 0.22–0.47; *p* < 0.0001), Jacobs et al. [84] found no corresponding reduction in transfusion rates (RR 1.02; 95% CI 0.93–1.12; *p* = 0.644) [84]. Anticoagulation-free protocols may be considered for patients with severe coagulopathy or thrombocytopenia but are generally regarded as inferior [81–84, 87].

### Adverse events in RCA

Citrate can critically decrease divalent cations like calcium and magnesium in the blood. Citrate is metabolized to bicarbonate, which can cause metabolic alkalosis, and its sodium load (as trisodium citrate) may induce hypernatremia. In cases where citrate metabolism is impaired, accumulation may result in metabolic acidosis with an elevated anion gap and an increased total/ionized calcium ratio. Systematic reviews consistently reported a higher incidence of hypocalcemia (ionized calcium < 0.7–1.0 mmol/L) under RCA [81–84]. The review of Tsujimoto et al. [83] reported more cases of alkalosis with RCA [83]. However, this analysis did not include the RICH trial [88], the largest RCT on this topic. Regarding thrombocytopenia, findings are inconsistent with some systematic reviews showing lower rates with RCA [81, 82] and some showing no difference [83, 84].

### RCA in liver failure

Citrate is primarily metabolized in the liver to bicarbonate. Thus, hepatic dysfunction or severe lactic acidosis may reduce its clearance, leading to citrate accumulation [89, 90]. Citrate accumulation manifests as increased calcium requirements, elevated total/ionized calcium ratios, and metabolic acidosis with an increased anion gap. While earlier concerns suggested higher risks in liver failure, more recent studies show that significant citrate toxicity is uncommon. Tan et al. [91] linked elevated baseline lactate (> 4 mmol/L) and delayed lactate clearance to greater risk of citrate accumulation in liver failure, supporting prior findings [89, 91].

A systematic review of nine randomized and non-randomized studies (*n* = 348) in liver failure patients found no significant mortality difference between RCA (58.9%) and heparin (47.4%) [85]. Citrate accumulation occurred in 5.3% and metabolic acidosis in 26.4% of RCA patients. Similarly, Peng et al. [92] reported citrate accumulation in 6.7% of cases, with a mortality rate of 45.9% [92], and Zhang et al. [93] found an incidence of 12% (95% CI 3–22%) [93].

Current evidence does not support considering liver failure as absolute contraindications for RCA (*recommendations 4.4*,* 4.5)*. However, RCA should be avoided in cases of progressive lactic acidosis with impaired lactate clearance (*recommendation 4.6*).

### Low molecular weight heparins

Low-molecular-weight heparins (LMWHs) offer improved bioavailability and longer half-life compared to unfractionated heparin (UFH). In AKI, LMWHs can accumulate, and their monitoring requires anti-factor Xa activity measurements. Typically, IHD involves an initial dose and reduced second dose, while continuous modalities use a bolus followed by continuous infusion.

RCTs comparing LMWH to UFH in RRT are small and yield inconsistent results. Reeves et al. [94] (*n* = 47) and Garcés et al. [95] (*n* = 40) found no significant differences in filter patency or bleeding [94, 95], while Joannidis et al. [96] (*n* = 40) reported significantly longer filter lifetimes with enoxaparin (30.6 ± 25.3 h vs. 21.7 ± 16.9 h; *p* = 0.017) [96]. Tsujimoto et al. [83] reviewed these trials and found no clear differences in bleeding or thrombocytopenia risk [83]. The Cochrane review by Natale et al. [97] focused on IHD and similarly found no statistically significant differences [97]. In the review of Zhou et al. [87] LMWHs were ranked higher for filter lifespan (51.9%) compared to UFH (23.1%) [87] (*recommendation 4.7)*.

### Argatroban for patients with HIT type II

In cases of HIT-II, the discontinuation of heparins and substitution with alternative anticoagulants is standard to prevent thromboembolic events. Argatroban, a synthetic direct thrombin inhibitor, is approved for HIT-II management. It is primarily metabolized hepatically and is not significantly cleared by standard dialysis filters.

An unpublished RCT by Shi et al. [98] involving 104 IHD patients reported a higher bleeding risk compared to heparin (RR 1.92, 95% CI 0.62–6.00), while a second RCT by Sun et al. [99] with 101 patients found no significant differences in bleeding, filter runtimes, or serious adverse events [99]. Observational data from Link et al. [100] and retrospective studies [101–103] support its use in both IHD and CRRT. For critically ill patients, especially those with liver dysfunction, a significantly reduced starting dose of 0.1–0.5 µg/kg/min is recommended to avoid overdosing [102]. A recent Cochrane analysis focused only on Argatroban use in chronic dialysis patients undergoing IHD but relied exclusively on unpublished data [97] (*recommendation 4.8)*.

Other anticoagulants in this setting, such as bivalirudin, were not discussed as these are not approved for use in RRT in Germany/Austria.

5. **Dose of Renal Replacement Therapy**

**Table:** 

5.1- We recommend that for continuous RRT a dose of 20–25 ml/kg/h be administered	A ⊕⊕⊕⊝
5.2- We recommend that for intermittent dialysis modalities, the dose be determined based on dialysis membrane, blood flow rate, dialysate flow rate, and in case of hemodiafiltration, convective filtration volume. Treatment time and frequency should be guided by clinical and laboratory parameters, particularly electrolytes and acid-base balance	Expert consensus
5.3- We recommend paying particular attention to the risk of disequilibrium when initiating RRT and adjusting the dose accordingly	Expert consensus
5.4- We do not recommend the use of high-volume hemofiltration in patients with sepsis or septic shock	0 ⊕⊝⊝⊝

### Dose in CRRT

Studies comparing higher versus lower dialysis or filtration doses have primarily aimed at improving the control of uremia and enhancing the clearance of medium-sized molecules. In particular, during the treatment of sepsis, it was hypothesized that higher filtration intensity could improve clinical outcomes.

In clinical practice, the prescribed dose often differs from the actual delivered dose due to factors such as interruptions for transport, filter clotting, or equipment set-up time. To account for these interruptions and ensure that the target dose of 20–25 ml/kg/h is achieved, a slightly higher dose (e.g., 25–30 ml/kg/h) is typically prescribed in clinical settings. A total of seven systematic reviews and a Cochrane Review were evaluated. The Cochrane analysis by Fayad et al. [8] and all systematic reviews consistently found no survival benefit from a higher dialysis dose (35 ml/kg/h) compared to a lower dose (20 ml/kg/h) with respect to 30-day mortality.

A multicenter retrospective study from Japan examined the effects of various doses of CRRT [104]. In patients receiving < 10 ml/kg/h, urea and creatinine levels increased. In those patients receiving 10–15 ml/kg/h, urea levels decreased by approximately 20%; in the 15–20 ml/kg/h group 35%; and in the > 20 ml/kg/h group by around 70% over seven days. Creatinine levels followed a similar trend. These findings suggest that a minimum effluent dose of at least 20 ml/kg/h is needed for effective uremia control. However, this evidence is observational, and no RCTs have specifically evaluated the lower limit of dosing.

Current recommendations are primarily based on two large RCTs, ATN [5] and RENAL [105]. The prescribed dose in the high-intensity group was 36.2 ± 3.8 ml/kg/h, with an actual delivered dose of 35.8 ± 6.4 ml/kg/h. In the lower-dose group, the prescribed dose was 21.5 ± 4.3 ml/kg/h and the actual delivered dose was 22.0 ± 6.1 ml/kg/h. There was no significant difference in 60-day mortality (OR 1.09; 95% CI, 0.86–1.40; *P* = 0.47). Similarly, the RENAL trial compared 40 ml/kg/h with 25 ml/kg/h and found no difference in mortality (OR 1.00; 95% CI, 0.81–1.23; *P* = 0.99).

Five of the seven systematic reviews also examined renal recovery and adverse effects. Renal recovery did not differ between high- and low-intensity groups (RR 0.98; 95% CI, 0.94–1.01; moderate-quality evidence). However, side effects were more frequent with higher-dose therapies, particularly hypophosphatemia, as reported in both the ATN and RENAL trials (*recommendation 5.1)*.

### Dose in intermittent RRT

In the intensive care setting, an intensive dose of intermittent procedures is usually defined as daily dialysis compared to three sessions per week. Urea clearance (Kt/V) and the urea reduction rate (URR) are the main indicators used to measure the effectiveness of dialysis procedures for patients requiring chronic dialysis. Kt/V is the ratio of urea clearance (K) to treatment time (t), expressed in relation to the urea distribution volume (V). For dialysis patients, a Kt/V of at least 1.2 and a URR of at least 65% are considered to be the minimum acceptable standards. These parameters can also, in principle, be used for AKI patients [106–108], but they are primarily evaluated for clinically stable patients with constant organ function. However, given the rapidly changing organ functions of critically ill patients, who are at an increased risk of dysequilibrium and have varying proportions of endogenous and mechanical clearance, we recommend checking the adequacy of the dialysis dose using laboratory diagnostic parameters such as creatinine, urea, phosphate and acid-base status.

In an RCT involving 160 patients with AKI, daily dialysis was associated with improved survival compared to dialysis 3-times per week (28% vs. 46% mortality; *p* = 0.01) [109]. In contrast, the ATN trial, which also evaluated intensified IHD, found no significant benefit in terms of mortality [5]. Similarly, the Hannover Dialysis Outcome Study, which randomized 156 patients to either intensified or standard dialysis using a SLED (sustained low-efficiency dialysis) protocol, found no mortality benefit [110]. The intensity in this study was guided by target urea concentrations: <90 mg/dL (intensified) versus 120–150 mg/dL (standard).

The systematic review by Van Wert et al. [111] examined three randomized and quasi-randomized studies on intensified IHD and found no significant differences in mortality (RR 0.89; 95% CI, 0.77–1.03) or in the rate of long-term dialysis dependence (RR 1.15; 95% CI, 0.92–1.44). Importantly, the review did not differentiate between dialysis modalities [111]. Similarly, Wang et al. [112] found no difference in 28-day mortality in their meta-analysis (RR 1.04; 95% CI, 0.92–1.18) [112].

Because intermittent procedures are highly efficient, particular attention must be paid to the risk of dialysis disequilibrium syndrome (DDS). DDS refers to a spectrum of neurological symptoms resulting from the rapid removal of osmotically active solutes, such as urea, leading to a sudden drop in serum osmolality. This creates an osmotic gradient between extracellular and intracellular compartments, promoting fluid shifts into cells—especially in the brain—and potentially resulting in cerebral edema.

Given the dynamic clinical course of critically ill patients, it is recommended to individualize the dialysis dose for each session by setting specific targets for dialysate flow, blood flow, and treatment duration (*recommendations 5.2*,* 5.3)*.

### High-volume hemofiltration

In contrast to studies on dose intensity, which compare higher doses (approximately 35–40 ml/kg/h) with standard doses (usually around 20 ml/kg/h), trials of high-volume hemofiltration (HVHF) specifically aimed to enhance the removal of inflammatory mediators in septic patients. In these studies, doses of up to 100 ml/kg/h were used. The concept of HVHF has been controversially discussed in the literature, and several hypotheses have been proposed. These include the Peak Concentration Hypothesis [113], the Threshold Immunomodulation Hypothesis [114], the Mediator Delivery Hypothesis [115], and the Monocyte HLA-DR Expression Hypothesis [116]. One key unresolved issue is how effective immunomodulation is achieved in a non-selective procedure that removes both pro- and anti-inflammatory cytokines.

To clearly differentiate HVHF from trials evaluating standard versus high-intensity RRT, the guideline group defined HVHF as a dose ≥ 50 ml/kg/h. The evidence from systematic reviews is inconsistent. Two meta-analyses including RCTs reported a mortality benefit from HVHF (Junhai et al. [117] RR 0.88 for mortality decrease (95% CI, 0.81–0.96; *p* = 0.004); Huang et al. [118] OR 1.66 (95% CI, 1.36–2.01; *p* < 0.001) for improved survival). However, all other systematic reviews failed to demonstrate a survival benefit for high-volume therapy in patients with sepsis.

Due to the small, heterogeneous study designs, potential regional bias, and generally low quality of evidence, a clear recommendation in favor of HVHF cannot be made (*recommendation 5.4)*.

6. **Pharmacology in Renal Replacement Therapy**

**Table:** 

6.1- The starting dose of an anti-infective agent should be based on the current distribution volume	Expert consensus
6.2- The starting dose should correspond to general dosage recommendations and should not be reduced	Expert consensus
6.3- We recommend that a starting dose should be administered as a short infusion to achieve rapid saturation and effective concentration, even if continuous or prolonged administration is planned subsequently	A ⊕⊕⊝⊝
6.4- If effective antiinfective levels are unknown, the maintenance dose should be based on total clearance. Total clearance should be estimated from residual renal function and extrarenal clearance, including machine clearance	Expert consensus (strong)
6.5- The continuous or prolonged administration of time-dependent anti-infectives under therapeutic drug monitoring may be considered in continuous procedures to achieve a more reliable pharmacokinetic/pharmacodynamic target attainment (defined target ranges), and may be preferred over short infusions	0 ⊕⊝⊝⊝
6.6- We recommend that in the case of IHD, a short infusion or prolonged administration of a time-dependent anti-infective agent may be preferable to continuous administration	Expert consensus
6.7- We recommend that at the end of IHD, depending on the pharmacokinetic properties of the anti-infective agent and the dialysis dose applied, an additional dose be administered according to evidence-based, recognized guidelines to restore adequate anti-infective levels	Expert consensus
6.8- We suggest that Therapeutic Drug Monitoring should be used for antibiotics such as β-lactams, vancomycin, and aminoglycosides in patients receiving RRT, if available	B ⊕⊝⊝⊝

### Starting dose

The starting dose should be chosen based on pharmacokinetic principles, particularly the patient’s apparent volume of distribution. Lipophilic agents (fluoroquinolones, metronidazole, rifampicin, linezolid, and chloramphenicol) have high volumes of distribution and lower plasma levels, whereas hydrophilic antibiotics (β-lactams, aminoglycosides, vancomycin) remain mostly in plasma and interstitial fluid.

Conditions such as hypoalbuminemia or edema may increase the volume of distribution, potentially leading to subtherapeutic levels. The SAFE study reported that 40–50% of ICU patients had serum albumin levels < 25 g/dL [119]. For many β-lactams, volume of distribution can increase by one-third under these conditions [120], necessitating higher starting doses (*recommendations 6.1*,* 6.2)*. KDIGO recommended increasing starting doses by 25–50%, based on empirical estimates [2] (*recommendation 6.2)*. One loading dose is usually sufficient, except for drugs like teicoplanin, which require 3–5 loading doses due to its large volume of distribution.

### Maintenance dose

The maintenance dose is determined by drug *clearance* and PK/PD targets relative to the minimum inhibitory concentration (MIC) of the pathogen. Most antibiotics have a molecular weight between 500 and 1,000 Da and can be removed by high-flux dialysis membranes, assuming the drug is not highly protein-bound or redistributed into peripheral compartments.

Most RRTs today use synthetic high-flux membranes, such as polyacrylonitrile or polysulfone. These membranes may exhibit some degree of adsorption, particularly during early phases of treatment or after a filter change. Experimental studies have shown that these membranes can bind a certain fraction of antibiotics like gentamicin, ciprofloxacin, and vancomycin [121]. However, the clinical relevance of adsorption is limited, as the effect is often modest and saturable.

Empirical maintenance dosing is necessary initially, particularly before Therapeutic Drug Monitoring (TDM) results are available, but standard dosing regimens are often inadequate during RRT [122]. In a study by Seyler et al. [123], 20–50% of patients on β-lactams during CRRT failed to reach therapeutic levels with standard maintenance doses [123]. Overall, TDM can be regarded as an appropriate method to achieve individual concentrations within the target range *(recommendation 6.8)*.

### Continuous or bolus application

The efficacy of an anti-infective agent can be characterized by PK/PD indices. These include.

- *Concentration-dependent* antibiotics (Cmax/MIC and/or AUC/MIC): aminoglycosides, fluoroquinolones, and daptomycin.
- For some antibiotics, *AUC/MIC* is considered the relevant parameter, including glycopeptides, linezolid, and tigecycline.
- *Time-dependent* antibiotics (fT > MIC, i.e., the time during which the free, unbound concentration of the antibiotic remains above the MIC of the pathogen): This includes β-lactams such as penicillins, cephalosporins, and carbapenems, as well as clindamycin.

From a pharmacodynamic perspective, it is advantageous to maintain an effective concentration of β-lactams above the MIC for a sufficiently long duration. Thus, continuous (24 h) or prolonged (3–4 h) infusions may be beneficial. Many studies have examined whether continuous or prolonged antibiotic administration is superior; however, these trials often excluded critically ill patients undergoing RRT.

In 2024, the large BLING III trial was published, which randomized 7,202 patients to receive either continuous or intermittent administration of piperacillin/tazobactam or meropenem for sepsis [124]. The primary endpoint, all-cause mortality at day 90, was not statistically significant (continuous 24.9% vs. intermittent 26.8%; absolute difference − 1.9%, 95% CI − 4.9% to 1.1%; OR 0.91, 95% CI 0.81 to 1.01; *p* = 0.08). However, the secondary endpoint of clinical cure was significantly better in the continuous group (55.7% vs. 50.0%; absolute difference 5.7%, 95% CI 2.4% to 9.1%) [124]. Notably, patients undergoing RRT were excluded from the BLING III trial. At the same time, a new systematic review including RCTs was published, which included the results of the BLING III trial and concluded that continuous administration reduced 90-day mortality (RR 0.86, 95% CI 0.72–0.98) [125]. Despite these conflicting data, most national and international guidelines recommend prolonged administration of β-lactams to achieve better PK/PD targets [126–129].

Because many studies excluded patients undergoing RRT, evidence on prolonged or bolus administration of anti-infectives in RRT patients is limited *(recommendation 6.5)*. In the BLING II cohort, approximately 26% of patients in both groups received RRT and were included in a joint analysis [130]. The SMARRT study included patients with RRT and reported very high variability in measured antibiotic levels [131]. Jamal et al. (2015) showed that continuous administration of piperacillin and meropenem led to more stable steady-state concentrations, while short infusions resulted in higher peak concentrations under both CRRT and IHD [132, 133]. Studies concluded that continuous antibiotic administration during CRRT led to more reliable PK/PD target attainment and tended to outperform intermittent short infusions [134–137].

Due to the temporary, high clearance of some anti-infective agents during IHD, the literature recommends administering a supplementation dose at the end of IHD. The required supplementation dose depends on how effectively the substance is eliminated by the filter and dialysis procedure *(recommendations 6.6 and 6.7)*.

### Therapeutic drug monitoring (TDM)

In clinical practice, blood samples for TDM are usually measured at the end of the dosing interval before the next dose is applied. After 4–5 half-lives, it can be assumed that a steady state has been reached in blood and tissue. For intermittent short-infusion administration, a target trough level (free concentration, fC_min) 1× > MIC is considered appropriate. In continuous administration, a target level of 2–6 × > MIC is recommended. This target level depends on disease severity and the identified pathogen [126, 127, 138, 139].

It is difficult to evaluate the effects of TDM in critically ill patients undergoing RRT because most studies excluded patients with AKI or those undergoing RRT. One exception is the large retrospective study by Richter et al. [136], which included patients on RRT and found that 10.1% of patients overall were underdosed with piperacillin/tazobactam at the initial empirical dose [136].

The randomized TARGET study evaluated TDM-based individual dosing of piperacillin under continuous administration in 254 patients, about one-quarter of whom received RRT [140]. The primary endpoint, SOFA score, was not significantly different between groups (7.9 with TDM vs. 8.2 without TDM). Similarly, 28-day mortality (21.6% vs. 25.8%; RR 0.8, 95% CI 0.5–1.3; *p* = 0.44) and clinical cure (OR 1.9, 95% CI 0.5–6.2; *p* = 0.30) showed no significant differences. However, PK/PD target attainment was significantly improved with TDM (37.3% vs. 14.6%; OR 4.5, 95% CI 2.9–6.9; *p* < 0.001). Notably, 28-day mortality was significantly higher in patients with piperacillin concentrations > 96 mg/L compared to those with concentrations between 32 and 64 mg/L (33.7% vs. 8.3%; OR 4.21, 95% CI 1.4–12.5; *p* = 0.01) or 64–96 mg/L (33.7% vs. 19.7%; OR 2.5, 95% CI 1.1–5.8; *p* = 0.03). According to the authors, this was likely due to reduced renal elimination and subsequent drug accumulation in the most severely ill patients.

A systematic review by Sanz-Codina et al. [141] evaluated 10 RCTs on TDM effects [141]. Mortality and clinical cure were not significantly improved with TDM (RR 0.86, 95% CI 0.71–1.05 and RR 1.33, 95% CI 0.94–1.33, respectively). Conversely, PK/PD target attainment was significantly better (RR 1.41, 95% CI 1.13–1.76), as was reduced nephrotoxicity (RR 0.55, 95% CI 0.31–0.97).

The question of TDM effects in patients undergoing continuous and intermittent RRT was addressed in a systematic review by Matusik et al. [139] including 139 reports, who recommended TDM for aminoglycosides, β-lactams, glycopeptides, linezolid, and colistin due to the high variability of antibiotic levels, and suggested considering it for tigecycline, daptomycin, and fluoroquinolones [139].

Given the significant risk of incorrect dosing in patients with multiple organ failure and RRT, we recommend that TDM be performed whenever possible for critical antibiotics in life-threatening infections *(recommendation 6.8)*.

The topic of intoxications can be found in the Supplementary Material. Further Recommendations.

7. **Stopping Renal Replacement Therapy**

**Table:** 

7.1- Diuretics may be considered when discontinuing RRT in order to increase the amount of diuresis	0 ⊕⊝⊝⊝
7.2- We suggest the use of urine output as a predictive marker for the discontinuation of RRT	B ⊕⊝⊝⊝
7.3- We are unable to make a recommendation on a precise minimum urine output volume for successful termination of RRT; as guidance, a spontaneous urine output of 300–600 ml/day without diuretics may be considered indicative	0 ⊕⊝⊝⊝
7.4- The kinetic GFR, based on endogenous clearance, can be considered as a predictive factor after discontinuation of RRT to assess the endogenous concentrating capacity of the kidney	0 ⊕⊝⊝⊝
7.5- Persistent hyperkalemia (> 5.5 mmol/L) may indicate an increased risk of weaning failure from RRT and should be investigated using differential diagnosis before attempting weaning	0 ⊕⊝⊝⊝
E7.6- We are unable to make a recommendation regarding the use of new damage or function markers as predictive markers for discontinuing RRT	0 ⊕⊝⊝⊝
7.7- We suggest that persistent metabolic acidosis (pH < 7.3) should be recognized as a potential indicator of increased risk for weaning failure from RRT and that differential diagnosis be conducted before attempting weaning	B ⊕⊝⊝⊝
7.8- We suggest correction of fluid overload before attempting to discontinue RRT	B ⊕⊝⊝⊝

Criteria for discontinuing RRT may include an absence of the absolute indications that initially prompted its initiation. Our systematic literature search identified 20 studies that provided information on defining successful discontinuation. The mean duration used across these studies was 5.95 days, with eight studies choosing a period of 7 days. The consensus group considered a seven-day period an appropriate measure for defining successful primary discontinuation (weaning failure as reestablishment of RRT within seven days of intentionally stopping dialysis or filtration procedures).

Patients who successfully discontinued RRT had lower mortality rates than those who did not. A literature search found 14 studies reporting mortality data for a total of 3,017 patients. Among patients with successful discontinuation, 422 out of 1,752 died, compared to 694 out of 1,265 patients without successful discontinuation (in-hospital mortality) (OR 3.83; 95% [CI], 3.27–4.47; *p* < 0.0001).

### Loop diuretics

Two RCTs investigated whether administering diuretics immediately after discontinuing RRT could improve the success rate of discontinuation. Cantarovich et al. [142] studied 338 patients, comparing 35 mg/kg of furosemide to placebo; 235 patients received IHD and 95 received CRRT [142]. While diuretics increased urine output, they did not improve the rate of successful discontinuation. van der Voort et al. [143] reached a similar conclusion in a smaller RCT [143]. Additionally, three observational studies and four retrospective analyses found an association between higher urine output at the end of RRT and successful discontinuation.

The systematic review by Katulka et al. including 23 observational studies confirmed that diuretics consistently increased urine output during RRT weaning [144]. However, the available RCTs demonstrated that despite increased urine output, the rate of successful discontinuation did not improve [142, 143, 145]. *(recommendation 7.1*,* 7.2)* An interesting approach is the use of FST to evaluate renal reserve before discontinuing RRT [145]. Studies on this method are still pending.

### Weaning protocols

In recent years, five observational and retrospective studies have investigated various protocols and algorithms. Despite limited evidence, these studies indicated that specific protocols facilitated a more consistent cessation of RRT [146, 147]. Baeg et al. [146] identified four variables that correlated well with actual weaning success: mean arterial pressure (MAP) between 50 and 78 mmHg, blood urea nitrogen below 35 mg/dL (± 12.5 mmol/L), potassium level below 4.1 mmol/L on the day of termination, and urine output greater than 300 mL on the day after RRT discontinuation [146].

### Urine output

Diuresis volume is the parameter that correlates best with successful discontinuation of RR [144, 148, 149]. However, at present, no consensus exists regarding a definitive urine output threshold required before discontinuation of RRT. Thresholds reported in individual studies vary widely between 191 ml/day [150] and 1700 ml/day [151]. In clinical practice, a urine output threshold of 300 or 500 ml/day is frequently used which aligns with studies showing 70% unsuccessful weaning rate with a diuresis below 0.3 ml/kg/h [152, 153]. Uchino et al. [154] found that patients with 400 ml/day without diuretics, and 2300 ml/day with diuretics, had an 80% probability of successful RRT discontinuation [154]. Based on low to moderate evidence, a pragmatic approach is to recommend a minimum urine output of 300–600 ml/day (or 0.3 ml/kg/h), which may be associated with a 70–80% probability of success *(recommendation 7.3)*.

### Kinetic GFR

Creatinine alone is not a fully reliable marker because its levels are influenced by both endogenous renal clearance and clearance via the dialysis machine. A similar limitation likely applies to cystatin C, which has been associated with an increased risk of weaning failure when elevated, but is also substantially cleared during dialysis [155, 156]. To overcome this limitation, kinetic eGFR estimation models have been developed. These models assess the change in creatinine over time, including the Chen formula [151], which demonstrated an AUC of 0.87 in a small retrospective study for predicting successful discontinuation *(recommendation 7.4)*.

### New biomarkers

Recent research has explored whether new structural biomarkers can predict successful discontinuation of RRT. Katulka et al. [144] found an AUC of 0.65 for serum NGAL [144]. Other studies reported AUCs in the range of 0.66–0.88 for NGAL [156–159]. In contrast, a larger prospective observational study by Stads et al. [160] showed that although urine NGAL was elevated two days after RRT discontinuation (776 vs. 277 ng/ml; *p* = 0.005), only an incremental serum creatinine ratio was an independent predictor of unsuccessful weaning (AUC 0.76) [160]. Overall, biomarkers such as NGAL, cystatin C, and others (e.g., FABP, KIM1, proenkephalin) tend to be elevated in cases of weaning failure, but threshold values and predictive accuracy (AUCs) vary widely *(recommendation 7.5)*.

### Negative indicators

Hyperkalemia and acidosis are two potential factors that may negatively impact the successful weaning of patients from RRT. A meta-analysis by Wang et al. [148] summarized two observational studies that investigated the effect of acidosis on unsuccessful discontinuation of RRT [148]. Among the 754 patients included, the mean difference was 0.16 (95% CI: 0.06–0.31; *p* = 0.03; heterogeneity *p* = 0.70, I² = 0%), indicating a statistically significant association *(recommendation 7.6)*.

A retrospective Brazilian cohort study involving 316 patients receiving RRT found that a higher pH—but not elevated potassium levels—was associated with a higher rate of successful RRT discontinuation [161] *(recommendation 7.7)*.

Fluid overload has been identified as one of the leading indications for resumption of RRT. Volbeda et al. [162] reported that 88% of respondents in a Dutch survey resumed therapy due to fluid overload [163]. Data on fluid balance were reported in nine studies identified through a systematic literature search. Four of these studies reported a correlation between a positive fluid balance and unsuccessful discontinuation of RRT [160, 164–166]. In contrast, van der Voort et al. [143] observed a non-significant trend [143], and Chen et al. [158] found no association between fluid overload and failure to discontinue RRT [158] *(recommendation 7.8)*.

## Conclusions

RRT needs ongoing evaluation and timely adjustments to treatment strategies, particularly in critically ill patients whose clinical status is often highly dynamic, necessitating ongoing evaluation and timely adjustments to treatment strategies. This guideline provides evidence-based recommendations for the optimal management of RRT in this setting.

## Supplementary Information

Supplementary Material 1.

Supplementary Material 2.

## Data Availability

All data generated or analyzed during this study are included in this published article and its supplementary information can be found at *https://register.awmf.org/de/leitlinien/detail/040-017*.

## Abbreviations

AKI: Acute Kidney Injury
CKD: Chronic Kidney Disease
CRRT: Continuous Renal Replacement Therapy
IHD: Intermittent Hemodialysis
KDIGO Kidney Disease: Improving Global Outcomes
PIRRT: Prolonged Intermittent Renal Replacement Therapy
RCT: Randomized Controlled Trial
SLEDD: Sustained Low Efficient Daily Dialysis
TDM: Therapeutic drug monitoring
