# Supplementary material for: Multidisciplinary guidelines on renal replacement therapy in intensive care medicine
Source: Crit Care. 2026 Jan 14;30:46. doi: 10.1186/s13054-025-05817-6 (PMC12849416; doi:10.1186/s13054-025-05817-6)
Supplement: Supplementary file 1 — Supplementary Material 1. [file 13054_2025_5817_MOESM1_ESM.docx]

***Evidence Report***

**Guideline on Renal Replacement Therapy in the Intensive Care Unit**

[*https://register.awmf.org/de/leitlinien/detail/040-017*](https://register.awmf.org/de/leitlinien/detail/040-017)

Deutsche Interdisziplinäre Vereinigung für Intensiv- und Notfallmedizin (DIVI)* - *German Interdisciplinary Association for Intensive Care and Emergency Medicine*

Deutsche Gesellschaft für Internistische Intensivmedizin und Notfallmedizin (DGIIN)* *- German Society for Internal Intensive Care Medicine and Emergency Medicine*

Deutsche Gesellschaft für Anästhesie und Intensivmedizin (DGAI) *– German Society of Anaesthesia and Intensive Care Medicine*

Deutsche Gesellschaft für Nephrologie (DGfN) – German Society of Nephrology

Deutsche Gesellschaft für Innere Medizin (DGIM) - German Society of Internal Medicine

Deutsche Gesellschaft für Kardiologie (DGK) - German Society of Cardiology

Deutsche Gesellschaft für Neurointensiv- und Notfallmedizin (DGNI) - German Society for Neurointensive Care and Emergency Medicine

Deutsche Gesellschaft für Pflegewissenschaften (DGP) - German Society for Nursing Sciences

Deutsche Sepsis Gesellschaft (DSG) - German Sepsis Society (DSG)

Österreichische Gesellschaft für Internistische und Allgemeine Intensiv- und Notfallmedizin (ÖGIAIN)

Austrian Society for Internal and General intensive Care and Emergency Medicine

Bundesverband Niere e.V. (BVN) - Federal Kidney Association

Deutsche Sepsis Hilfe (DSH) - German Sepsis Aid (DSH)

Content

[1 Start of Renal Replacement Therapy 7](#_Toc209691404)

[1.1 PRISMA start 9](#_Toc209691405)

[1.2 Systematic Reviews and AMSTAR2 10](#_Toc209691406)

[1.3 Risk of Bias: Trials Start 11](#_Toc209691407)

[1.4 RCTs: mortality 12](#_Toc209691408)

[1.5 Systematic Reviews: Mortality 14](#_Toc209691409)

[1.6 Systematic Reviews: renal recovery 16](#_Toc209691410)

[1.7 RCTs: renal recovery 18](#_Toc209691411)

[1.8 Furosemide Stress Test: PRISMA chart 20](#_Toc209691412)

[1.9 Trial evidence: Furosemide Stress Test 21](#_Toc209691413)

[2 Diffusion / convection 23](#_Toc209691414)

[2.1 PRISMA Chart: Diffusion vs convection 25](#_Toc209691415)

[2.2 Systematic Reviews and AMSTAR2 26](#_Toc209691416)

[2.3 Systematic Reviews: Diffusion and Convection 27](#_Toc209691417)

[2.4 Trial evidence: Diffusion vs Convection – RCTs from 2021 28](#_Toc209691418)

[2.5 Trial evidence: Intracranial Pressure and RRT 29](#_Toc209691419)

[2.6 Trial evidence: Rhabdomyolysis 30](#_Toc209691420)

[3 Continuous and Intermittent Renal Replacement Therapy 31](#_Toc209691421)

[3.1 PRISMA Chart: continuous or Intermittent Kidney Replacement in intensive Care Medicine 32](#_Toc209691422)

[3.2 Systematic Reviews und AMSTAR2 33](#_Toc209691423)

[3.3 Systematic Reviews evidence table: CVVH versus IHD Renal Replacement Procedure 34](#_Toc209691424)

[3.4 Systematic Reviews CVVH versus IHD - Outcome mortality 35](#_Toc209691425)

[3.5 Systematic Reviews CVVH vs SLED - Outcome mortality 36](#_Toc209691426)

[3.6 Systematic Reviews: CVVH versus IHD - Outcome renal recovery 37](#_Toc209691427)

[3.7 Systematic Reviews: CVVH versus SLED - Outcome Renal recovery 38](#_Toc209691428)

[3.8 Trials evidence table: CVVH versus IHD - Outcome Renal recovery 40](#_Toc209691429)

[3.9 Systematic Reviews: CVVH versus IHD - Outcome hemodynamic stability 43](#_Toc209691430)

[3.10 Trials: CVVH versus IHD - Outcome hemodynamic stability 44](#_Toc209691431)

[3.11 Trials evidence table: Outcome SAE - Thrombocytopenia and Transfusion Frequency 45](#_Toc209691432)

[3.12 PRISMA Chart: Intracranial Pressure in continuous versus Intermittent Renal Replacement Procedures 46](#_Toc209691433)

[3.13 Trials evidence table: Intracranial Pressure in continuous versus Intermittent Renal Replacement Procedures 47](#_Toc209691434)

[1.1. Systematic Reviews: Fluid Overload 49](#_Toc209691435)

[3.14 Trials evidence table: Fluid Overload 50](#_Toc209691436)

[3.15 PRISMA Chart: Liver Failure 52](#_Toc209691437)

[3.16 Trials: Liver Failure 53](#_Toc209691438)

[4 Anticoagulation of Renal Replacement Therapy 54](#_Toc209691439)

[4.1 PRISMA Chart 1: RCA and UFH 56](#_Toc209691440)

[4.2 PRISMA Chart 2: LMWH 57](#_Toc209691441)

[4.3 PRISMA Chart 3: Argatroban 58](#_Toc209691442)

[4.4 Systematic Reviews und AMSTAR2 59](#_Toc209691443)

[4.5 Synopsis Systematic Reviews: Citrate 60](#_Toc209691444)

[4.6 Systematic Reviews: Citrate vs Heparin mortality 61](#_Toc209691445)

[4.7 Systematic Reviews: Renal recovery from AKI 62](#_Toc209691446)

[4.8 Systematic Reviews : Filter Runtimes 62](#_Toc209691447)

[4.9 Systematic Reviews: Bleeding 62](#_Toc209691448)

[4.10 Systematic Reviews evidence table: Hypocalcemia 63](#_Toc209691449)

[4.11 Systematic Reviews: Acid/Base Complications 64](#_Toc209691450)

[4.12 Systematic Reviews: Thrombocytopenia 64](#_Toc209691451)

[4.13 Systematic Reviews: RCA in liver failure 65](#_Toc209691452)

[4.14 Systematic Reviews: LMWH versus UFH 66](#_Toc209691453)

[4.15 Trials: Further studies LMWH versus UFH 67](#_Toc209691454)

[4.16 Systematic Reviews: Argatroban 68](#_Toc209691455)

[4.17 Trials: Further studies Argatroban 69](#_Toc209691456)

[5 Dose of Renal Replacement Therapy 70](#_Toc209691457)

[5.1 PRISMA Chart Dose 72](#_Toc209691458)

[5.2 Systematic Reviews und AMSTAR2 Dose 73](#_Toc209691459)

[5.3 Systematic Reviews synopsis: CVVH – Standard vs. high dose 74](#_Toc209691460)

[5.4 Trials: CVVH - Standard vs. high dose 75](#_Toc209691461)

[5.5 Trials: Standard vs. high dose in IHD 76](#_Toc209691462)

[5.6 Observational Studies: Standard vs. High Dose in IHD 77](#_Toc209691463)

[5.7 Systematic Reviews: Standard vs. high volume dose 78](#_Toc209691464)

[5.8 Trials: Standard vs. high-volume dose 79](#_Toc209691465)

[6 Pharmacology in Renal Replacement Therapy 81](#_Toc209691466)

[6.1 PRISMA Chart 83](#_Toc209691467)

[6.2 Systematic Reviews synopsis PK/PD 1 84](#_Toc209691468)

[6.3 Systematic Reviews synopsis PK/PD 2 85](#_Toc209691469)

[6.4 Systematic Reviews synopsis PK/PD 3 86](#_Toc209691470)

[6.5 Systematic Reviews continuous vs prolonged antibiotic application: AMSTAR2 88](#_Toc209691471)

[6.6 Systematic Reviews: Continuous vs prolonged anti-infective doses 89](#_Toc209691472)

[6.7 Trials: New studies (without RRT) - continuous vs prolonged anti-infective drugs 90](#_Toc209691473)

[6.8 Trials: New studies (with RRT) - continuous vs prolonged anti-infective doses 91](#_Toc209691474)

[6.9 Systematic Reviews AMSTAR: TDM 93](#_Toc209691475)

[6.10 Systematic Reviews: TDM in AKI and RRT 94](#_Toc209691476)

[6.11 Trials: TDM in AKI and RRT 95](#_Toc209691477)

[6.12 Trials: Lithium 96](#_Toc209691478)

[7 Stopping Renal Replacement Therapy 98](#_Toc209691479)

[7.1 PRISMA Chart 100](#_Toc209691480)

[7.2 Systematic Reviews und AMSTAR2 101](#_Toc209691481)

[7.3 Evidence table: Definition for successful weaning 102](#_Toc209691482)

[7.4 Trials: Effects of diuretics 103](#_Toc209691483)

[7.5 Trials: Weaning protocols and scoring systems 105](#_Toc209691484)

[7.6 Trials: Diuresis, creatinine and urea in urine as biomarkers 107](#_Toc209691485)

[7.7 Trials: Diuresis minimum quantity 107](#_Toc209691486)

[7.8 Trials: Blood biomarkers and successful weaning 109](#_Toc209691487)

[7.9 Trials: Successful weaning and urine biomarkers 112](#_Toc209691488)

[7.10 Trials: Successful weaning and fluid overload 112](#_Toc209691489)

[8 References 115](#_Toc209691490)

Abbreviations

AKI Acute Kidney Injury

AMSTAR Assessing the Methodological Quality of Systematic Reviews

AUC Area under the Curve

CI Confidence Interval

Clr Clearance

CKD Chronic Kidney Disease

CRRT Continuous Renal Replacement Therapy

CVVHD Continuous venovenous hemodialysis

CVVH Continuous venovenous hemofiltration

RBC Red Blood Cell

FST Furosemide Stress Test

HV High Volume

IHD Intermittent hemodialysis

IHF Intermittent hemofiltration

KDIGO Kidney Disease Improving Global Outcomes

LMWH Low Molecular Weight Heparin

LV Low Volume

n.a. not applicable

US Newcastle Ottawa Scale for non-randomized studies

OR Odds Ratio

OT Observational Trial

PEO Patients – Interventions – Outcomes (no comparator)

PICO Patients – Interventions – Comparison – Outcomes

PK/PD Pharmakokinetic/Pharmacodynamic

RCT Randomized Controlled Trial

REV Review

ROB Risk of Bias

RR Risk Ratio

RRT Renal Replacement Therapy

RT Retrospective Trial

SAE/AE Serious Adverse Events/Adverse Events

SLED Sustained Low Efficient Dialysis

SR Systematic Review

TDM Therapeutic Drug Monitoring

UFH Unfractionated Heparin

UO Urine Output

Vd Volume of distribution

Database search

databases: Scopus, PubMed and Cochrane

exclusion: children

years: 2002 – 2023
language: english, german

# Start of Renal Replacement Therapy

PICO questions

| P | In critically ill patients with AKI and RRT |
| --- | --- |
| I | Does early initiation of RRT improve outcomes in terms of  a) mortality  b) renal recovery  Does early use of RRT reduce the rate of RRT-associated complications |
| C | Compared to late use |
| O | mortality, renal recovery, SAE/AE |

PIO questions

| P | In critically ill patients with AKI and RRT |
| --- | --- |
| I | 3 What are absolute and relative indications for initiation?  4 What is the predictive value of the furosemide stress test (FST) in determining the indication for initiating RRT?  5 What role do new renal biomarkers play in the indication for RRT? |
| O | mortality, renal recovery, SAE/AE |

PICO Search Terms

| **Patient** | **Intervention** | **Comparison** | **Outcome** |
| --- | --- | --- | --- |
| critical care  critically ill  intensive care | renal replacement therapy  dialysis  hemofiltration  hemodiafiltration | standard of care | recovery of renal function |
| acute kidney injury  acute renal failure | start  early  beginning  delayed  timing |  | duration of RRT  mortality |
|  |  |  |  |

Database Search string

(renal replacement therapy OR dialysis OR hemofiltration OR hemodiafiltration)

AND (acute kidney injury OR acute renal failure)

AND (critical care OR intensive care OR critically ill)

AND NOT[TI] pediatric OR neonates OR children

AND (timing OR early OR delayed OR start OR beginning OR late)

## PRISMA start

**Screening**

**Included**

**Eligibility**

**Identification**

Records identified through Cochrane (n = 431 )

Off topic (n = 1370)

Full-text articles assessed for eligibility (n = 104)

Non-controlled trials(n = 63)

RCTs(n =19 )

Systematic Reviews (n = 16)

Meta-analysis (n=4)

Cochrane Review (n =2)

Records identified through Scopus searching(n = 523 )

Narrative Reviews, editorials, notes excluded (n = 50)

Records identified through PubMed (n = 720)

Records after duplicates removed(n = 1524)

Records screened(n = 154)

## Systematic Reviews and AMSTAR2

## Risk of Bias: Trials Start

|  | **Random sequence generation (selection bias)** | **Allocation concealment (selection bias)** | **Blinding of participants and personnel (performance bias)** | **Blinding of Outcome assessment (detection bias)** | **Incomplete Outcome data (attrition bias)** | **Selective Reporting (Reporting bias)** | **Other bias** |
| --- | --- | --- | --- | --- | --- | --- | --- |
| Bouman 2002 (28) | + | + | ? | + | + | + | ? |
| Sugahara 2004 (202) | ? | ? | ? | + | - | + | ? |
| Forest 2015 (224) | + | + | ? | + | + | + | + |
| Gaudry 2016 (75) | + | + | ? | + | + | + | + |
| Zarbock 2016 (245) | + | + | ? | + | + | + | + |
| Lumlertgul 2018 (128) | + | + | ? | + | + | + | + |
| Srisawat 2018 (196) | + | + | ? | + | + | + | + |
| Barbar 2018 (19) | + | + | ? | + | + | + | + |
| Bagshaw 2020 (17) | + | + | ? | + | + | + | + |
| Gaudry 2021 (74) | + | + | ? | ? | + | + | + |

## RCTs: mortality

| **Study** | **Design** | **N** | **Setting** | **Definition of randomization groups** | **Primary outcome: mortality** | **Patients with RRT** | **Mortality** | **RRT modality** | **Notes** |
| --- | --- | --- | --- | --- | --- | --- | --- | --- | --- |
| Bouman 2002 (28) | RCT  2 Centers | 106 | mixed | early: within 12 hours after meeting the following criteria:  urine excretion <30ml/h for 6h and creatinine clearance <20ml/min  late: BUN > 40mmol/L, potassium >6.5mmol/L or severe pulmonary edema | 28d | early: 70/70 (100%)  late: 30/36 (83.3%) | early: 20/70 (28.6%)  late: 9/36 (25.0%) | LVHF |  |
| Sugahara 2004 (202) | RCT  mono | 28 | surgical | early: Urine output <30ml/h in 3h or <750ml in 24h  late: Urine output < 20ml/hin 2h or <500ml in 24h | 14d | 100%, patients without RRT were excluded | early: 2/14 (14.3%)  late: 12/14 (85.7%) | CRRT |  |
| Forest 2015 (224) | RCT  multi | 100 | mixed | early: min 2 of the following criteria: 2-fold creatinine increase, RO < 6ml/kg in 12h, NGAL ≥400ng/ml  late: severe hyperkalemia (>6mmol/l), severe pulmonary edema, severe metabolic acidosis (bicarbonate <10mmol/l) | 90d | Q: 48/48 (100%)  late:33/52 (63%)) | early: 18/48 (37.5%)  late: 19/52(36.5%) | HVHF, CRRT, PIRRT, IHD |  |
| Gaudry 2016 (75) | RCT  multi | 619 | mixed | early: KDIGO3 (within 6h)  late: severe hyperkalemia (>6mmol/l), severe diuretic-refractory pulmonary edema, severe acidosis (pH<7.15), urea >40mmol/l, oligo-/anuria > 72h | 60d | early: 305/311 (98.1%)  late: 157/308 (51.0%) | early: 150/311 (48.5%)  late: 153/308 (49.7%) | CRRT, IHD |  |
| Zarbock 2016 (245) | RCT  mono | 231 | mixed | early: KDIGO2 and pNGAL >150ng/ml (within 8h)  late: within 12h after KDIGO stage 3 and pNGAL >150ng/ml | 90d | early: 112/112 (100%)  late: 108/119 (90.8%) | early: 44/112 (39.3%)  late: 65/119 (54.7%) | CRRT |  |
| Lumlertgul 2018 (128) | RCT  multi | 118 | mixed | early: AKI (at any stage) and lack of response to the FST (within 6 hours)  late: only if one of the following criteria is present: BUN ≥100mg/dl, potassium >6mmol/l, bicarbonate <12mmol/l, pH <7.15, PaO2/FiO2 <200, severe pulmonary oedema | 28d | early: 57/58 (98.3%)  late: 45/60 (75%) | early: 36/58 (62.1%)  late: 35/60 (58.3%) | CRRT, IHD, PIRRT |  |
| Srisawat 2018 (196) | RCT  2 Centers | 40 | mixed | early: AKI, every RIFLE stage  late: severe metabolic acidosis (pH<7.20), potassium >6.2mmol/l, severe diuretic-resistant pulmonary edema, persistent oliguria or anuria, urea >40mg/dl | 28d | Q: 20/20 (100%)  late: 12/20 (60%) | early: 10/20 (50.0%)  late: 9/20 (45.0%) | CRRT |  |
| Barbar 2018 (19) | RCT  multi | 488 | septic shock | early: within 12h to RIFLE-F  late: severe hyperkalemia (K>6.5mmol/l), severe diuretic-resistant pulmonary edema, severe metabolic acidosis (pH <7.15), no renal recovery after 48h | 90d | early: 239/246 (97.2%)  late: 149/242 (61.6%) | early: 138/239 (57.7%)  late: 128/238 (53.8%) | CRRT, PIRRT, IHD |  |
| Bagshaw 2020 (17) | RCT  multi | 2927 | mixed | early: 2-fold increase in creatinine from baseline, urine output <6mmol/kg in 12h  late: min 1 of the following criteria: potassium >6mmol/l, pH<7.20, bicarbonate ≤12mmol/l, PaO2/FiO2 <200 AND fluid overload, persistent AKI for min 72h after randomization | 90d | early: 1418/1465 (96.8%)  late: 903/1462 (61.8%) | early: 643/1465 (43.9%)  late: 639/1462 (43.7%) | CRRT, PIRRT, IHD | CAVE: Late high crea levels!  CAVE: Clinical *equipoise* Bias |
| Gaudry 2021 (74) | RCT  multi | 278 | mixed | inclusion of critically ill patients with severe AKI (KDIGO 3) and oliguria for >72h or BUN >112mg/dl.  late: immediate inclusion  very late: RRT only if hyperkalemia or severe metabolic acidosis or pulmonary edema or BUN ≥ 140mg/dl | days between Rando and d28 (alive+ without RRT) | W: 134/137 (98.0%)  late: 111/141 (78.7%) | Day 28  late: 52/137 (38%)  very late: 63/141 (45%)  Day 60  LATE: 60/137 (44%)  Very late: 77/141 (55%) | CRRT, IHD | got RRT:  late: 134/137 (98%)  very late: 111/141 (79%)  modality :  late: 39% CRRT, 60% IHD  Very late: 40% CRRT, 58% IHD |
| **Result** | **10 RCTs** | **4935** | **Mostly mixed** | **early RRT (currently KDIGO3) does not reduce patient mortality compared to late use (KDIGO3 plus clinical indication or longer-lasting KDIGO3)**  **very late use of RRT appears to increase mortality at day 60 (AKIKI2)**  **CAVE: considerable differences in the definition of the groups!**  **CAVE: significant differences in modalities**  **CAVE: differences in the primary objective criteria** | | | **OR 1.04**  **95%CI**  **(0.92-1.16) p=0.53**  **without**  **Gaudry 2021** | **Modality mixed** | **GRADE**  **mortality ⊕⊕⊕⊝ moderate evidence** |

## Systematic Reviews: Mortality

|  | **Design** | **Included studies (n)** | **Mortality**  **early vs. late** | **I^2^** | **AMSTAR** |
| --- | --- | --- | --- | --- | --- |
| Karvellas 2011 (103) | SR | 15 (2 RCT, 4 OT, 9RT) | 28d: GOLD 0.45 (95% CI, 0.28-0.72) | 78% | Critically low |
| Liu 2014 (125) | SR | 11 (2 RCT, 9 RT) | 28d: OR 0.29 (95% CI, 0.16-0.52) | 56% | Critically low |
| Wang 2016 (227) | SR | 12 | OR 0.78 (95% CI, 0.52-1.19) | 67% | Critically low |
| Wierstra 2016 (230) | SR | 9 | OR 0.67 (95% CI, 0.38-1.15) | 84% | Critically low |
| Feng 2017 (64) | SR | 9 RCT | RR: 0.93 (95% CI, 0.74-1.18) | 57% | Critically low |
| Xu 2017 (236) | SR | 6 | RR 0.93 (95% CI, 0.68-1.26) | 65% | Critically low |
| Yang 2017 (239) | SR | 9 RCTs | RR 0.98 (95% CI, 0.78-1.23) | 60% | Critically low |
| Fayad 2018 (63) | Cochrane | 5 | 30d: RR 0.83 (95% CI, 0.61-1.13) | 52% | High |
| Broom 2019 (21) | SR | 62 (11 RCT, 51 OT/RT) | RCTs: OR 0.78 (95% CI, 0.52-1-19)  OTs: OR 0.69 (95% CI, 0.49-0.96)  RT: OR 0.61 (95% CI, 0.41-0.92) | RCT 63%  OT 86%  RT 91% | Critically low |
| Chaudhuri 2019 (34) | SR | 7 RCTs | 28d: GOLD 0.90 (95% CI, 0.70-1.15) | 10% | Critically low |
| Read 2019 (119) | SR | 5 | 28d: GOLD 0.76 (95% CI, 0.58-1.00)  90d: OR 0.79 (95% CI, 0.59-1.06) | 76% | Critically low |
| Pasin 2019 (154) | SR | 10 RCTs | OR 0.99 (95% CI, 0.66-1.50) | 74% | Critically low |
| Xiao 2019 (234) | SR | 11 | RR 0.99 (95% CI, 0.84-1.17) | 44% | Critically low |
| Gaudry 2020 (73) | SR | 10 | 28d: RR 1.01 (95% CI, 0.91-1.13) | 0% | Low |
| Zhang 2020 (248) | SR | 18 RCTs | RR 0.98 (95% CI, 0.89, 1.08) | 2% | Critically low |
| Bhatt 2021 (22) | SR | 14 RCTs | Total: RR 0.99 (95% CI, 0.89-1.10)  d30: RR 1.00 (95% CI, 0.91-1.09)  d90: RR 1.00 (95% CI, 0.88-1.13) | O: 36%  D30: 15%  D90: 44% | Low |
| Li 2021 (118) | SR | 11 | 28d: RR 1.01 (95% CI, 0.94-1.09)  90d: RR 1.02 (95% CI, 0.92-1.13) | 28d: 0%  90d: 37% | Critically low |
| Naorungroj 2021 (142) | SR | 8 RCTs | 28d: RR 1.01 (95% CI, 0.94-1.09) | 0% | Critically low |
| Pan 2021 (149) | SR | 10 RCTs | OR -0.04 (95% CI, -0.16-0.07) | 59% | Low |
| Fayad 2022 (62) | Cochrane | 12 RCTs | 30d: RR 0.97 (95% CI, 0.87-1.09) | 29% | High |
| Li 2022 (120) | SR | 15 RCTs | 28d: RR 1.01 (95 CI, 0.94-1.08)  60d: RR 1.00 (95% CI, 0.91-1.11)  90d: RR 1.01 (95% CI, 0.94-1.08) | 28d: 0%  60d: 11%  90d: 36% | Critically low |
| **Result** | **20 SR**  **2 Cochrane** |  | **2 SR (and limited *Besen 2019*) showed survival advantage, 18 SR showed no advantage** | **High heterogeneity** | **GRADE ⊕⊕⊕⊝ evidence moderate** |

## Systematic Reviews: renal recovery

|  | **Definition renal recovery** | **renal recovery**  **early vs. late** | **I^2^** |
| --- | --- | --- | --- |
| Karvellas 2010 (102) | RRT independency | OR 0.62 (95% CI, 0.34-1.13) | 70% |
| Liu 2014 (125) | n.a. | n.a. | n.a. |
| Wang 2016 (227) | n.a. | n.a. | n.a. |
| Wierstra 2016 (230) | n.a. | n.a. | n.a. |
| Feng 2017 (64) | n.a. | n.a. | n.a. |
| Xu 2017 (236) | n.a. | n.a. | n.a. |
| Yang 2017 (239) | Renal function *recovery* | RR 1.02 (95% CI, 0.88-1.19) | 59% |
| Fayad 2018 (63) | *renal recovery* | RR 0.83 (95% CI, 0.66-1.05) | 54% |
| Broom 2019 (21) | *recovery* criteria of each study | RR 1.01 (95% CI, 0.98-1.04) | 22% |
| Chaudhuri 2019 (34) | n.a. | n.a. | n.a. |
| Read 2019 (119) | n.a. | n.a. | n.a. |
| Pasin 2019 (154) | n.a. | n.a. | n.a. |
| Xiao 2019 (234) | Renal function *recovery* | RR 1.02 (95% CI, 0.97-1.07) | 32% |
| Gaudry 2020 (73) | n.a. | n.a. | n.a. |
| Zhang 2020 (248) | Dialysis independency | RR 0.75 (95% CI, 0.47-1.20) | 0% |
| Bhatt 2021 (22) | n.a. | n.a. | n.a. |
| Li 2021 (118) | n.a. | n.a. | n.a. |
| Naorungroj 2021 (142) | n.a. | n.a. | n.a. |
| Pan 2021 (149) | RRT independency | OR -0-03 (95% CI, -0.14-0.09) | 51% |
| Fayad 2022 (62) | *recovery* of kidney function | RR 1.07 (95% CI, 0.94-1.22) | 55% |
| Li 2022 (120) | n.a. | n.a. | n.a. |
| **Result** | **Definitions heterogeneous**  **8/21 SR analyzed renal recovery** | **no SR showed significant benefits at early start for renal recovery** | **High heterogeneity** |
| **GRADE renal recovery ⊕⊕⊝⊝ evidence Low** | | | |

## RCTs: renal recovery

| **Studies** | **Design** | **N** | **Setting** | **Dialysis-free**  **Day 28** | **Dialysis-free**  **Day 60** | **Dialysis-free**  **Day 90** | **Other definition** |
| --- | --- | --- | --- | --- | --- | --- | --- |
| Bouman 2002 (28) | RCT  2 Centers | 71 | mixed | F. 38/39 (97.4%)  late: 22/22 (100%) |  |  |  |
| Sugahara 2004 (202) | RCT  mono | 28 | surgical |  |  |  | day 14 (recovery from dialysis):  early: 10/12 (83.3%)  late: 2/2 (100%) |
| Forest 2015 (224) | RCT  multi | 100 | mixed |  |  | Q: 48/48 (100%)  late: 50/52 (96.2%) |  |
| Gaudry 2016 (75) | RCT  multi | 619 | mixed | early: 157/179 (87.7%)  late: 161/178 (90.4) | early: 154/157 (98.1%)  late: 147/155 (94.8%) |  |  |
| Zarbock 2016 (245) | RCT  mono | 231 | mixed |  |  | early: 60/112 (88.2%)  late: 46/119 (38.7%) |  |
| Lumlertgul 2018 (128) | RCT  multi | 118 | mixed | early: 51/58 (87.9%)  late: 50/60 (83.3%) |  |  |  |
| Srisawat 2018 (196) | RCT  2 Centers | 40 | mixed | early: 21/22 (95.5%)  late: 19/25 (76.0% |  |  |  |
| Barbar 2018 (19) | RCT  multi | 488 | septic shock | early: 117/134 (87%)  late: 123/140 (88%) |  | early: 99/101 (98%)  late: 107/110 (97%) |  |
| Bagshaw 2020 (17) | RCT  multi | 2927 | mixed |  |  | early: 729/814 (89%)  late: 766/815 (94%) | reduction <25% of baseline GFR at d90  early: 264/403 (65.5%)  late: 255/427 (59.7%) |
| Gaudry 2021 (74) | RCT  multi | 278 | mixed | early: 72/85 (84.7%)  late: 71/78 (91.0%) | late: 74/77 (96.1%)  very late: 63/64 (98.4%) |  |  |
| **Result** | **10 RCTs** | **4900** | **mostly mixed** | **early: 456/503**  **late: 446/517**  **OR 0.96, 95%CI 0.65-1.40, P=0.816** | **early: 228/234**  **late: 210/219**  **OR 1.63, 95%CI 0.57-4.65, P=0.36** | **early: 936/1075**  **late: 969/1096**  **OR 0.88, 95%CI 0.68-1.14, P=0.34** | **GRADE**  **renal recovery ⊕⊕⊝⊝ evidence Low** |

## Furosemide Stress Test: PRISMA chart

Records identified through PubMed searching (n = 76)

**Screening**

**Included**

**Eligibility**

**Identification**

Records identified through Cochrane searching (n = 14)

Records after duplicates removed(n = 96)

Records screened (n = 19)

Off topics (n = 77)

Full-text articles assessed for eligibility (n = 8)

Non-controlled trials (OT n = 4)

(RT n = 1)

RCTs (n = 2)

Systematic Reviews

(n=1)

Cochrane Review (n = 0)

Records identified through Scopus searching (n = 42)

Reviews, publishers
(n = 11)

## Trial evidence: Furosemide Stress Test

Abbr.: AUC: Area Under the Curve, UO: Urine Output, FST: Furosemide Stress Test, spec.: specificity, Sens: sensitivity, NOS: Newcastle Ottawa Scale, ROB: Risk OF Bias

| **Study** | **Design** | **N** | **AUC** | **Sensitivity/specificity**  **KDIGO3 Prediction** | **Outcome** | **ROB/US** | **Notes** |
| --- | --- | --- | --- | --- | --- | --- | --- |
| Chen 2020 (36) | SR | 1366 |  | Sens: 0.84 (95% CI 0.72–0.91)  Spec. 0.77 (95% CI 0.64–0.87) | AUC for AKI 0.81 (95% CI 0.74–0.87)  AUC for RRT 0.88 (95% CI 0.82–0.92) | See AMSTAR chart | 7 trials included |
| Lumlertgul 2018 (128) | RCT | 118 |  | FST neg 118:  - 58 early RRT  - 60 late RRT | 28-d *mortality*  early: 36/58 (62.1%)  late: 35/60 (58.3%)  p=0.68 | 5/6 ROB2 | in case of negative FST, always RRT  62.1 vs 58.3% p=0.68, ns |
| Zhang 2023 (247) | RCT | 187 | RRT AUC 0.966 | FST++ 18/48 (37.5%) 🡪 RRT  FST-- 124/139 (89.2%) 🡪 RRT |  | 9/9 US |  |
| Chawla 2013 (35) | OT | 77 | KDIGO3 AUC 0.87 | 2h UO 200 ml  Sens 87.1%  Spec 84.1% |  | 9/9 US | 77 pooled with KDIGO3 retrospective and prospective cohort |
| Koyner 2015 (111) | OT | 77 | RRT prediction  AUC 0.87  P=0.001 |  | 2h UO after FST better than urine biomarkers to predict KDIGO3 (P,0.05). | 9/9 US | biomarker |
| Rewa 2019 (171) | OT | 92 | KDIGO3 AUC 0.87 | 2h UO 200 ml  Sens 73.9%  Spec 90.0% |  | 9/9 US |  |
| Bolgiaghi 2021 (23) | OT | 40 |  | FST -- 190 [175- 250]ml/2h  FST++ 1225 [1000-1800] ml/2h | Correlation of Renal Index and FST | 8/9 US |  |
| Pon 2021 (160) | OT | 80 | KDIGO3 AUC 0.89 | 300 ml 2h FST  Sens 82.1%  Spec 82.7% |  | n.a. |  |
| Fox ,Decleene 2023 (68) | OT | 225 |  | FST++ 88/225 (39.1%) | MAP >75 mmHg 60/104 (57.7%) FST++  MAP < 75 mmHg 28/121 (23.1%) FST++  OR 4.53, 95% CI, 2.55-8.74, *P* < 0.001 with vasopressors: 30.4% vs 68.2%, *p* = 0.026 | 9/9 US | MAP <75 mmHg OR 4.53, 95% CI, 2.55-8.74, *P* < 0.001, hypotension correlated with FST |
| Meersch 2023 (134) | OT | 208 | AUC for RRT:  FST alone 0.78 (0.74 – 0.85) | FST– 108  FST++ 100 |  | 9/9 US | Combination of FST with CCL14 for RRT Prediction  AUC for RRT:  FST+CCL14 0.8 |
| Su 2024 (201) | RT | 499  &  3188 | AUC RRT 0.81  AUC RRT 0.59 |  |  | good | Cardiac Surgery  2 Cohorts analyzed  FST increases RRT prediction |
| Sakhuja 2019 (179) | RT | 687 | RRT AUC 2h FST 0.71  RRT AUC 6h FST 0.67 p=.02) | 300 ml 6h FST  Sens 64.2%  Spec 68.2 |  | good | Retrospective from Charts  6h AUC was better than 2h FST to predict RRT |
| Matsuura 2018 (132) | RT | 95 | KDIGO3 AUC 0.84 |  |  | good | 18/95 developed post surgery KDIGO3 |
| **Result** | **2 RCT**  **8 OT**  **2 RT** | **FST to KDIGO3 and RRT Prediction: GRADE renal recovery ⊕⊕⊝⊝ evidence low**  **FST AUC for KDIGO 0.84-0.89**  **FST AUC for RRT 0.79-0.96**  **Sensitivity of FST 64.2 – 87.1% for KDIGO3 prognosis, specificity of FST 68.2 – 90.0% for KDIGO3 prognosis** | | | | | |

# Diffusion / convection

PICO Questions

| P | In critically ill patients with AKI and RRT |
| --- | --- |
| I | Does convection lead to improved outcomes compared to diffusion  Does the combination of convection and diffusion lead to improved outcomes |
| C | compared to diffusion or convection alone? |
| O | mortality, renal recovery, hemodynamic stability, filter life, SAE/AE |

PIO Questions

| P | In critically ill patients with AKI and RRT |
| --- | --- |
| I | Do certain patient groups (sepsis, intracranial pressure, rhabdomyolysis, etc.) benefit more or less from convection vs. diffusion or the combination? |
| O | mortality, renal recovery, hemodynamic stability, filter life, SAE/AE |

PICO search terms

| **Patient** | **Intervention** | **Comparison** | **Outcome** |
| --- | --- | --- | --- |
| critical care  critically ill  intensive care | filtration  convection | dialysis  diffusion | *recovery* of renal function  duration of RRT  *mortality*  SAE/AE  hemodynamic stability |
| acute kidney injury  acute renal failure |  |  |
|  |  |
| renal replacement therapy  dialysis, hemofiltration, hemodiafiltration |  |  |

Database Search string

(renal replacement therapy OR dialysis OR hemofiltration OR hemodiafiltration)

AND (acute kidney injury OR acute renal failure)

AND (critical care OR intensive care OR critically ill)

AND (dialysis OR diffusion OR hemodialysis)

AND (filtration OR convection OR hemofiltration OR hemodiafiltration) AND NOT[TI] pediatric OR neonates OR children OR infants

## PRISMA Chart: Diffusion vs convection

Records identified through PubMed searching (n=432)

**Screening**

**Included**

**Eligibility**

**Identification**

Records identified through Cochrane searching

(n = 222)

Records after duplicates removed

(n = 774)

Records screened

(n = 120)

Off topic

(n = 654)

Full-text articles assessed for eligibility (n = 82

Non-controlled trials

(observational n = 27)

(retrospective = 15)

RCTs (n = 37)

Systematic Reviews (n = 3)

Records identified through Scopus searching

(n = 248)

Pediatric, narrative Reviews unfinished studies excluded (n = 38)

## Systematic Reviews and AMSTAR2

|  | **Design** | **N** | **RRT mode** | **Outcome Parameter** | **Variables** | **Significance** | **Note** |
| --- | --- | --- | --- | --- | --- | --- | --- |
| Zha 2019 (246) | SR | 22 RCT  3360 patients  no blinding | Different "blood purification methods" for sepsis-AKI  IHD, CVVH, CVVHDF, CVVHD, HV-CVVH, PMX, PMX+RRT (+adjuvant drugs e.g. AP) | *Survival rate*  *renal recovery*  *ICU-stay* | PRC vs no PRC  Comparing different RRT strategies | OR 0.75  n.s. | no significant differences between the different RRT strategies. no study comparing CVVH vs CVVHDF |
| Snow 2021 (193) | SR | No diffusion vs convection  Not AKI –  Non RRT | "Blood purification" in sepsis  CVVH, Endotoxin removal, Nonspecific adsorption, Cytokine removal, CPFA, Combined CVVH and adsorption, Plasma exchange (PE) | *mortality* | Pooled Analysis  *Trial sequential analysis* | OR 0.49 (p<0.001) | Inadequate sample size to conclude a mortality benefit from extracorporeal blood purification in sepsis |
| Côté 2022 (44) | SR | 615 pat.  With ICU-AKI | Diffuse vs Konv at ICU AKI  Intermittent IHF/IHDF vs IHD only | *mortality*  *renal recovery*  *Clearance*  *Hemodynamic stab.*  *Circuit loss*  *Inflammation* | ns  ns  small molecules conv.↓  ns  ns  not investigated | RR 1.23 | no significant difference between intermittent. convective methods to intermittent. diffusive RRT |
| **Result** | **3 SR** |  | **Data heterogeneous** | | | **GRADE mortality**  **⊕⊝⊝⊝ evidence very low**  **GRADE renal recovery**  **⊕⊝⊝⊝ evidence very low** | |

## Systematic Reviews: Diffusion and Convection

## Trial evidence: Diffusion vs Convection – RCTs from 2021

|  | **Design** | **Patients** | **N** | **RRT mode** | **Outcome Parameter** | **Result** | **P-value** | **ROB/NOS** | **Note** |
| --- | --- | --- | --- | --- | --- | --- | --- | --- | --- |
| Abdelsalam 2021 (1) | RT | Chronic  Dialysis  retrospective | 1115 | Intermittent  HDF (n=215) vs. HD (n=900) | S-PO4  S-Ca  Kt/V  Hb | ↓ for HDF  ↑ for HDF  ↑ for HDF  ↑ for HDF | P<0.001  P<0.012  P<0.0001  P<0.024 | good | Additional convection in CHD improves dialysis goals |
| Jang 2021 (93)  Jang ,Lee 2022 (94) | RCT | ICU patients with septic AKI | 100 | CVVHDF (n=49) vs CVVH (n=47)  same BF, same effluent  same baseline characteristics | 7-, 28-, and 60-day mortality  Reduction of Crea, HST, ß2-MG, APACHE II, SOFA | no difference  no difference | n.s.  n.s. | abstract only | With the same net effluent, CVVH and CVVHDF no difference in substance removal and survival |
| Xu 2022 (235) | RCT | ICU patients | 60 | CVVHDF (n=30) vs. CVVH (n=30) | *Filter Life Span*  *Molecular solute clearance*  - Urea and creatinine  - IL-6, ß2-MG, Myoglobin  -TMP | CVVH shorter  (8 p.m. vs. 37.5 p.m.)  no difference  ↑ at CVVH  ↑ at CVVH | p=0.002  p>0.05  p<0.01 | ROB 3/6 | CVVHDF leads to longer filter life, with the same clearance of small molecules but lower clearance of medium molecules |
| Mann 2023 (131) | RCT  Quasi randomized by time blocks | ICU patients with severe AKI III | 161  (591 Filter) | CVVHD (n=67, 245 Filter) vs. predilution CVVH (n=94, 346 Filter) | *Filter Life Span*  *Numbers of Filters used*  *In hospital mortality* | CVVH shorter  (79% of CVVHD)  11.8% vs 21.2% ns | p=0.02  n.s.  n.s. | ROB 2/6 | CVVHF led to significantly shorter filter life time than CVVHD (diffusion) |
| **Result** | **3 RCTs**  **1 RT** |  |  |  |  |  | **ROB 2-3/6 available**  **GRADE SAE/AE ⊕⊕⊝⊝ evidence low** | | |

## Trial evidence: Intracranial Pressure and RRT

|  | **Design** | **N** | **Patient cohort** | **RRT mode** | **Outcome** | **ROB**  **US**  **Bias --** | **Notes** |
| --- | --- | --- | --- | --- | --- | --- | --- |
| Davenport 1989 (47) | RCT | 7 | hepatic encephalopathy | IHF vs CAVHF | ICP IHF: 8.4 ±1.5 to 12.6±1.8 mmHg  ICP CAVH: 15.6 ±5.2 to 11.7±2.3 mmHg | 3/6 ROB |  |
| Wu 2013 (233) | RCT | 10 | ICB | SLED vs CVVH | but no difference between method p=0.46 | 3&6 ROB | ICP increase in both methods,  Approx. 0.1% for CVVH, approx. 0.4% for SLED in the first 4 hours |
| Davenport 1993 (46) | OT | 30 | hepatic encephalopathy | IHF vs CVVH | ICP IHF: const.  ICP CAVH: 15.6 ±5.2 to 11.7±2.3 mmHg | 5/9 US | MAP decline in the first hour at IHF with a reduction in CPP 35 ±8% |
| Ronco 1999 (177) | OT | 12 | only AKI with RRT | IHD vs CVVH | IHD: *gray matter* 52.3 ±5.2 to 38.9 ±5.3 *white matter* von 36.7 ±3.5 to 24.8 ±3.2 Hounsfield units, average delta ‐26.7% | 6/9 US |  |
| Johansen 2017 (98) | OT | 12 | CKD patients | Lowflux-IHD vs  Pre-IHDF | MRI: no differences between procedures, *Grey matter*: HD: r2 = 0.83; HDF: r2 = 0.73, White matter: r2 = 0.02; HDF: r2 = 0.004 | 4/9 US | *Total brain volume*  IHD: +1.8 ± 1.7% (18.7 ± 17.4mL)  Pre-IHDF: +2.0 ± 0.9% (22.3 ± 10.7 mL) |
| Venkatasubba Rao 2018 (218) | OT | 14 | CKD in general | IHD | osmolarity mean -6.4 mOsm/L  (SD 6.6) from pre- to post-dialysis and  VIPS edema index (E-Dex) +9.7% (SD  12.9) p = 0.037). | 6/9 US | *Volumetric Integral Phase-shift*  Spectroscopy (VIPS) to evaluate fluid shifts under HD: Osmo drops about 6-7 mmosmol/l during IHD |
| **Result** | **2 RCT**  **4 OT** | **Partly CKD patients**  **Heterogeneous techniques**  **Small collectives without control** | | | **Data insufficient**  **GRADE ⊕⊝⊝⊝ evidence low** | | |

## Trial evidence: Rhabdomyolysis

|  | **Design** | **N** | **RRT mode** | **Myoglobin removal** | **Outcome** | **ROB/US**  **Bias--** | **Notes** |
| --- | --- | --- | --- | --- | --- | --- | --- |
| Peltonen 2007 (156) | RCT | 16 | HDF FX100 | HDF 28.1% Myoglobin removal, diuresis 14.2% P=0.001 | Plasma Myoglobin decrease, *HDF*; 9731 95% CI 3672-5345 µg/l | 3/6 ROB | HDF (FX100) and forced diuresis eliminated more Myoglobin than forced diuresis alone (28.1 vs 14.2%) |
| Potter 2013 (161) | RCT | 38 | FX1000HDF  Pre + postHDF vs postHDF | 70.2+-3.6 vs 42.6% Myoglobin  Reduction ratio | n.a. | 4/6 ROB | Pre + postdilution was more effective than postdilution at Myoglobin removal |
| Kirsch 2017 (108) | RCT | 39 | Medium cutoff filters vs highflux | MCO: 58.7 (3.18) or 62.7(2.39) vs  19.9 (2.39) Highflux HD  35.6(2.46) HDF | n.a. | 4/6 ROB | CKD patients  MCO filter (HD general) removed Myoglobin more efficiently than high-flux |
| Weidhase 2020 (229) | RCT | 70 | HCO CVVHD  CVVHDF highflux  with RCA | 12h after RRT start  HCO 8.3 median ml/ml  Highflux 2.3 median ml/ml | ICU mortality 62.9 vs 30.3% in HCO arm (90d *mortality* ns)  AE ns | 0/6 ROB | Better clearance at HCO CVVHD |
| Sorrentino 2011 (194) | OT | 6 | Extended single batch, highflux | 90.5 mL/min (52.4-126.3)  0.54 g/h (0.15-2.21) | n.a. | 4/9 US |  |
| Premru 2013 (162) | OT | 18 | HCO HDF | 90-94 ml/min  Myoglobin RR 80% | n.a. | 1/7 US | Half of the Myoglobin removed in 3-5 hours, then only 7%/h |
| Suzuki 2021 (204) | RT | 12 | CVVHF  AN69, PMMA, PMMA high vol  (300 ml/min QD) | AN69 9.7 ml/min Myoglobin Clr; PMMA 13.7 ml/min  PMMA high volume 29.4 ml/min | n.a. | n.a. | High flow CVVHDF with highest clearance |
| **Result** | **4 RCT**  **2 OT**  **1 RT** | **Heterogeneous cohorts, different Filters**  **Technically, myoglobin elimination is possible (high evidence)** | | | **However, data available for outcome parameters are insufficient**  **renal recovery GRADE ⊕⊝⊝⊝ evidence very low** | | |

# Continuous and Intermittent Renal Replacement Therapy

PICO Questions

| P | In critically ill patients with AKI and RRT |
| --- | --- |
| I | Does the use continuous RRT |
| C | compared to intermittent RRT and PIRT improve outcomes? |
| O | mortality, renal recovery, hemodynamic stability, SAE/AE (thrombopenia, blood loss, transfusion frequency) |

PIO Questions

| P | In critically ill patients with AKI and RRT |
| --- | --- |
| I | Do certain patient groups (fluid overload, liver failure, intracranial pressure, sepsis) benefit more or less from continuous versus intermittent or prolonged RRT? |
| O | mortality, renal recovery, hemodynamic stability, filter life, SAE/AE |

PICO Search Terms

| **Patient** | **Intervention** | **Comparison** | **Outcome** |
| --- | --- | --- | --- |
| critical care, critically ill  intensive care | continuous | intermittent  prolonged | *recovery* of renal function |
| acute kidney injury  acute renal failure |  |  | duration of RRT |
|  |  |  | *mortality* |
| renal replacement therapy  dialysis, hemofiltration, hemodiafiltration |  |  | SAE/AE  hemodynamic stability |

Database Search string

(renal replacement therapy OR dialysis OR hemofiltration OR hemodiafiltration)

AND (acute kidney injury OR acute renal failure) AND (critical care OR intensive care OR critically ill)

AND (continuous OR convection OR CVVH OR CVVHF OR CVVHD OR CVVHDF) AND (intermittent OR diffusion)

## PRISMA Chart: continuous or Intermittent Kidney Replacement in intensive Care Medicine

Records identified through PubMed searching

(n = 1215)

**Screening**

**Included**

**Eligibility**

**Identification**

Records identified through Cochrane searching

(n = 530)

Records after duplicates removed

(n = 2068)

Records screened

(n =301)

Pediatric, no critical care, only *Trial* protocols, off topics excluded

(n = 1767)

Full-text articles assessed for eligibility

(n = 150)

Retrospective (n = 66)

Observational (n=43)

Records identified through Scopus searching

(n = 640)

RCTs (n =25)

Systematic Reviews (n =16)

off-topic excluded

(n = 151)

## Systematic Reviews und AMSTAR2

## Systematic Reviews evidence table: CVVH versus IHD Renal Replacement Procedure

| **Author / Year** | **Study Type / Patients / modality** | **Outcome** | **Comment** |
| --- | --- | --- | --- |
| Rabindranath 2007 (165) (Cochrane) | 15 RCTs (1550 Pt); CRRT vs IHD | Hospital mortality (RR 1.01, 95% CI 0.92 - 1.12), ICU mortality (RR 1.06, 95% CI 0.90 - 1.26), survivors without further RRT (RR 0.99, 95% CI 0.92 - 1.07), hemodynamic instability (RR 0.48, 95% CI 0.10 - 2.28) or hypotension (RR 0.92, 95% CI 0.72 - 1.16) increasing vasopressor therapy (RR 0.53, 95% CI 0.26 - 1.08). higher MAP at CRRT (MD 5.35, 95% CI 1.41 - 9.29) higher risk of clotting (RR, 95% CI 8.50 CI 1.14 - 63.33). | no benefit for CRRT in terms of mortality, renal recovery, hemodynamics (except higher MAD, but more clotting) |
| Virgo 2008 (152) | 30 RCTs, 8 prospective cohort studies; CRRT vs IHD | all-cause mortality (relative risk [RR], 1.10; CI 95%, 0.99-1.23; I^2^=0%) or further dialysis (RR, 0.91; 95% CI, 0.56-1.49; I^2^=0%). | no benefit for CRRT in terms of mortality and further need of dialysis |
| Schneider 2013 (185) | 7 RCTs, 16 observational studies with 472 and 3,499 survivors 30 RCTs, 8 prospective cohort studies; CRRT vs IRRT (IHD + hybrid) | dialysis-dependent survivors: no difference (RR 1.15 CI 95% 0.78–1.68], I2 = 0%); observational studies suggest a higher rate of dialysis dependence in survivors who initially received IHD (RR 1.99, CI 95% 1.53–2.59), I^2^ = 42% | no benefit for CRRT regarding and further need of dialysis in RCTs |
| Schoenfelder 2017 (186) | 13 RCTs, 16 observational studies involving 1,870 and 15,689 patients; CRRT vs IRRT (IHD + hybrid) | RCTs: mortality RR 1.03; 95% CI [0.94,1.14]; renal recovery RR 1.01; 95% CI [0.95,1.07]; Hypotension RR 0.92, 95% CI [0.94,1.14] | no benefit for CRRT in terms of mortality, renal recovery, hypotension |
| Nash 2017 (143) | 21 RCTs and large observational studies, CRRT vs IHD, CRRT vs SLED | RRT modality was not correlated with mortality (CRRT vs IHD: RR 1.00 [95%CI, 0.92–1.09], CRRT vs SLED: RR 1.23 [95%CI,1.00–1.51]) or dialysis dependence (CRRT vs IHD: RR 0.90 [95%CI,0.59–1.38], CRRT vs SLED: RR1.15 [95%CI,0.67–1.99]). | no benefit for CRRT compared to IHD or SLED for mortality or dialysis dependence |
| Zhao ,Chen 2020 (253) | 22 RCTs and OTs  CRRT vs IHD vs SLED | mortality: CRRT or SLED or IHD (RR 1.02, 95% CI 0.93-1.11, I^2^ = 0.0%, P = .921; less renal recovery: in AKI patients with CRRT compared to IHD or SLED (RR 0.81, 95%CI 0.79-0.83, I^2^ = 21.9%, P = .269; In a subgroup analysis, no difference between CRRT and SLED (RR 0.96, 95% CI 0.77-1.19, I^2^ = 7.6%, P = .364 | no benefit for CRRT compared to IHD or SLED for mortality or dialysis dependence |
| Ye 2021 (240) | 30 RCTs, 3,774 Patienten; CRRT vs IHD, CRRT vs SLED, IHD vs SLED; (Peritoneal Dialysis) | mortality: CRRT vs IHD 1.04 (95% CI 0.93–1.18); CRRT vs SLED 1.06 (95% CI 0.85–1.33); IHD vs SLED 1.02 (95% CI 0.79–1.31); renal recovery: CRRT vs IHD 1.15 (95% CI 0.91–1.45); CRRT vs SLED 0.88 (95% CI 0.65–1.19); IHD vs SLED 0.77 (95% CI 0.53–1.12) | no benefit for CRRT compared to IHD or SLED for mortality or dialysis dependence |
| **Result** |  | **Mortality GRADE ⊕⊕⊝⊝ evidence low** |  |

## Systematic Reviews CVVH versus IHD - Outcome mortality

| **Study** | **Design** | **N** | **Trials** | **Mortality** | **Notes** |
| --- | --- | --- | --- | --- | --- |
| Rabindranath 2007 (165) | SR | 1550 | 15 RCTs  until 10/2006 | *in hosp. mortality* (peer-reviewed only)  RR 1.03 (95%CI 0.92-1.16), I^2^ 33%, p=0.57 | no difference |
| Bagshaw 2008 (16) | SR | 1403 | 9 RCTs  until 12/2006 | OR 0.99; (CI95% 0.78 –1.26); p = 0.93; I^2^ 11% | no difference |
| Ghahramani 2008 (76) | SR | 1635 | 9 RCTs, 18 OT, 6 RT  until 9/2007 | 33 at trials: 0.77 (95% CI 0.62–0.95)  9 RCTs alone: OR 0.89 (0.63–1.24) p=0.95 | no difference (9 RCTs)  in OT/RT/RCT: CRRT better (33 trials – all qualities) |
| Virgo 2008 (152) | SR | 3190 patients RCT  2868 patients OTs | 30 RCTs, 8 OT  until 10/2007 | RR 1.10 (95%CI 0.99-1.23) I^2^ 0%– 9 RCTs, 989 patients 28d *mortality* | no difference |
| Zhang 2015 (249) | SR | 617 patients RCT  675 patients OTs | 7 RCTs, 10 OTs  until 2014 | 7 RCTs alone: 0.90; 95% CI, 0.74-1.11; P 5 0.3). 17 at the trials: 0.86; 95% CI, 0.74-1.00; P 5 0.05, | no difference (7 RCTs)  in OT: CRRT better (17 trials – all quality) |
| Nash 2017 (143) | SR | 5015 | 21 RCTs  until 5/2015 | CRRT vs IHD RR 1.00 (95%CI 0.92-1.09) I2 23%, p=0.99 | no difference |
| Schoenfelder 2017 (186) | SR | 617 pat. RCT  7682 pat. OT | 9 RCTs, 40 OTs  to 12/2014 | RCT: RR 1.03 (95%CI 0.94-1.14), I2 29%, p=0.51  OT: RR 1.21 (95%CI 1.07-1.37), I2 88%, p=0.002 | RCT: no difference  in OT: CRRT better (40 trials – all quality) |
| Zhao ,Chen 2020 (253) | SR | 521 pat. CRRT  424 pat. SLED | 8 RCT, OT SLED  to 6/2019 | *in hospital mortality*:  CRRT vs IHD RR 1.21 (95%CI 1.19-1.23) I2 0%, p=0.54 | no difference |
| Eat 2021 (240) | SR | 3774 pat.  CRRT, IHD, SLED | 15 RCTs, 4 OT  until 5/2020 | CRRT vs IHD: RR 1.04; 95% CI, 0.93–1.18 | no difference |
| Zhou 2021 (256) | SR | 621 pat. CRRT, 1503 pat. IHD, 333 pat. PD | 23 RCTs  to 6/2020 | CRRT vs IHD: RR 0.88 (95%CI 0.73-1.07), I2 0% | no difference |
| **Result** | **10 SR** | **10/10 SR no difference (RCTs)**  **3/10 SR CRRT less mortality when observational/retrospective with high patient numbers are included**  **Mortality GRADE ⊕⊕⊝⊝ evidence low** | | | |

## Systematic Reviews CVVH vs SLED - Outcome mortality

| **Study** | **Design** | **N** | **Trials** | **Dialysis dependency** | **Notes** |
| --- | --- | --- | --- | --- | --- |
| Zhao ,Chen 2020 (253) | SR | 521 pat. CRRT  424 pat. SLED | 8 trials SLED  (RCT, OT) until 6/2019 | SLED vs CRRT in *ICU mortality*  RR 1.09 (95%CI 0.88-1.35) I^2^ 0% p=0.64 | no difference |
| Al Dalbhi 2021 (6) | SR | 1160 | 6 RCTs  5 OTs until 2017 | SLED vs IHD  RR 0.67, (95% CI 0.44–1.00) I^2^ 0% P=0.05 | no difference |
| Eat 2021 (240) | SR | 3774 pat.  CRRT, IHD, PD, SLED | 15 RCTs  4 OT  until 5/2020 | CRRT vs. SLED:  RR 1.06; 95%, CI 0.85–1.33 | SLED better |
| Zhou 2021 (256) | SR | 621 pat. CRRT  1503 pat. IHD  333 pat. PD | 23 RCTs until 6/2020 | SLED vs CRRT:  RR 0.98 (95%CI 0.88-1.08), I^2^ 22% | no difference |
| Kovacs 2017 (110) | SR | 1564 pat. | 16 RCTs  until 2/2015 | SLED vs CRRT  RR 1.21 (95%CI 1.02-1.43), I^2^ 47%, p=0.03 | SLED better |
| Nash 2017 (143) | SR | 5015 pat. | 21 RCTs  until 5/2015 | CRRT vs SLED  RR 1.23 (95%CI 1.00-1.51) I^2^ 13% p=0.05 | no difference |
| **Result** | **4/6 SR no mortality difference between CRRT and SLED**  **2/6 SR moderately lower mortality with SLED (RR 1.06 and 1.21)**  **Mortality GRADE ⊕⊕⊝⊝ evidence low** | | | |  |

## Systematic Reviews: CVVH versus IHD - Outcome renal recovery

| **Study** | **Design** | **N** | **Trials** | **Renal recovery** | **Notes** |
| --- | --- | --- | --- | --- | --- |
| Rabindranath 2007 (165) | SR  Cochrane | 1550 Pt | 15 RCTs  until 10/2006 | RRT independence  RR 0.99 (95% CI 0.92 - 1.07) I^2^ 0% p=0.83 | no difference |
| Bagshaw 2008 (16) | SR | 1403 pat. | 9 RCTs  until 12/2006 | *Recovery*/RRT independence  OR 0.76 (95%CI, 0.28 –2.07) p = 0.59 | no difference |
| Ghahramani 2008 (76) | SR | 1635 pat. | 9 RCTs, 18 OT, 6 RT  until 9/2007 | DD RCT only 1.07 0.47–2.39 CRRT n=133 IHD n=139, no difference  DD all trails OR 0.44 (CI95% 0.34–0.58 CRRT better | only RCTs with DD as defined outcome: no difference  OTs/RTs: CRRT improves *renal recovery* |
| Virgo 2008 (152) | SR | 3190 RCT  2868 OTs | 30 RCTs, 8 OT  until 10/2007 | DD: RR 0.91 (95%CI 0.56-1.49 I^2^ 0%) | no difference |
| Schneider 2013 (185) | SR | RCT 472  OT 3499 | 7 RCTs, 16 OTs  until 6/2012 | DD only RCTs: RR 1.15 (95%CI 0.78-1.68) I^2^ 0% p=0.48  DD at trials: RR 1.73 (95% CI, 1.35–2.20) I^2^ 44%, p<0.001 | RCTs only: no difference (240 p.m.)  OTs: CRRT better *renal recovery* (1476 pat.) |
| Nash 2017 (143) | SR | 5015 pat. | 21 RCTs  until 5/2015 | dialysis dependence: CRRT vs IHD: RR 0.90 (95% CI 0.59-1.38) | no difference |
| Schoenfelder 2017 (186) | SR | 617 RCT  7682 OT | 9 RCTs, 40 OTs  until 12/2014 | *renal recovery* (all): RR 1.10 95% CI 1.05, 1.16) I^2^ 87%, p<0.01  *renal recovery* (RCTs): RR 1.01 (95%CI 0.95-1.02) I^2^ 0%, p=0.79  *renal recovery* (OTs): RR 1.17 (95% CI 1.09-1.24) I^2^ 58.4%, p<0.01 | RCTs only: no difference (1,870 pats.)  OTs: CRRT better *renal recovery* (15,689 patients)  (OT >10 times the number of patients) |
| Al Dalbhi 2021 (6) | SR | 1160 pat. | 6 RCTs, 5 OTs  until 2017 | *renal recovery*: RR 1.08, (95% CI 0.83–1.42; P=.56), I^2^ 63%  dialysis dependence RR=1.03, (95% CI 0.69–1.53; P=.89). I^2^0% | fluid removal: *no difference:* Std Mean Diff -0.24 (95% CI -0.72-0.24), p=0.32 I2 79% |
| Ye 2021 (240) | SR | 3774 CRRT, IHD, SLED | 15 RCTs, 4 OT  until 5/2020 | *renal recovery*:  CRRT better than IHD RR 1.15 (95% CI, 0.91–1.45) | CRRT better than IHD |
| Zhou 2021 (256) | SR | 621 CRRT, 1503 IHD | 23 RCTs  until 6/2020 | *renal recovery*:  CRRT vs IHD RR 1.05 (95%CI 0.83-1.35), I^2^=46.6% | no difference |
| **Result** | **10/10 SR – no differences *in renal recovery* when evaluating RCRs**  **4/10 SR – CRRT more *renal recovery* in observational/retrospective studies** | | | | **no differences in *renal recovery*:**  **GRADE ⊕⊕⊝⊝ evidence low** |

## Systematic Reviews: CVVH versus SLED - Outcome Renal recovery

| **Study** | **Design** | **N** | **Trials** | **Renal recovery** | **Notes** |
| --- | --- | --- | --- | --- | --- |
| Zhao ,Chen 2020 (253) | SR  SLED | 521 pat. CRRT  424 pat. SLED | 5 trials SLED  to 6/2019 | CRRT vs SLED *renal recovery*:  RR 0.96, 95% CI 0.77-1.19, I2 = 7.6%, P = 0.36 | no difference |
| Eat 2021 (240) | SR  CRRT  SLED | 3774 pat.  CRRT, IHD, PD, SLED | 15 RCTs  4 OT  until 5/2020 | CRRT vs SLED *renal recovery*:  RR 0.88; 95% CI, 0.65–1.19 | SLED better than CRRT |
| Nash 2017 (143) | SR  SLED  CRRT | 5015 pat. | 21 RCTs  to 5/2015 | CRRT vs SLED *dialysis dependence*:  RR 1.15 (95% CI 0.67-1.99) | no difference |
| Zhou 2021 (256) | SR  SLED  CRRT | 621 pat. CRRT  1503 pat. IHD  333 pat. PD | 23 RCTs  to 6/2020 | SLED vs CRRT *renal recovery*:  CRRT vs SLED RR 1.19 (95%CI 0.90-1.59), I2=15.1% | no difference |
| Zhang 2015 (249) | SR  SLED | 617 pat. RCT  675 pat. OTs | 7 RCTs  10 OTs  until 2014 | SLED vs CRRT *renal recovery*:  in RCTs: (RR, 1.12; 95% CI, 0.83-1.76; P =0.4, I2 5 65%).  in OTs: (RR, 1.14; 95% CI, 0.90-1.46; P=0.3; I2 5 0%). | no difference |
| Kovacs 2017 Kovacs 2017 (110) | SR  SLED | 1564 pat. | 18 RCTs  to 2/2015 | SLED vs CRRT *renal recovery*:  RR 0.87, (CI95% 0.63–1.20), I2 = 66%). | no difference |
| **Result** | **6 SR** | ***Renal recovery* SLED vs. CRRT** | | | **1 SR: SLED better than CRRT with RR 0.88**  **5 SR: no difference**  **GRADE ⊕⊕⊝⊝ evidence low** |

## Trials evidence table: CVVH versus IHD - Outcome Renal recovery

| **Study** | **Design** | **N** | **Collective** | **RRT mode** | **Outcome** | **ROB**  **critical** | **US** | **Notes** |
| --- | --- | --- | --- | --- | --- | --- | --- | --- |
| Forest 2014 (226) | RT | 4008 | database Canada 1996-2009 | 2004 CRRT  2004 IHD | More dialysis dependence after IHD:  HR, 0.75 (95% CI, 0.65–0.87) p < 0.0001 | n.a. | n.a. | CRRT better  matched to kidney function |
| Schefold 2014 (182) | RCT  CONVINTED | 252 | single ICU Germany | 122 CVVH  128 IHD | days at RRT 17.2 ± 37.1 vs 13.7 ± 17.9 p=0.35  RRT at 21d: 20 (32.3%) IHD vs 20 (29.9%) CRRT p=0.97  RRT at 60d 14 (26.4%) IHD vs 13 (22.8%) CRRT p=0.90 | 1/6 | n.a. | no difference |
| Liang 2016 (121) | RT | 638 | ICU Survivors 2000-2008 | 353 IHD  285 CRRT | CRRT vs IHD 90 d: OR, 1.19; 95% CI 0.91-1.55; P=0.20  CRRT vs IHD 365d: OR, 0.93; 95% CI 0.72-1.2; P=0.55 | n.a. | n.a. | no difference (multivariate analysis) unstable Pt. Were allocated to more CRRT |
| Truche 2016 (209) | OT | 1360 | OUTCOMEREA  prospective observationale French cohort | CVVH 544 (40.0 %) IHD 816 (60.0 %) | no difference composite PO  HR 1.00, 95%CI 0.77–1.29; p = 0.97).  *dialysis dependence*  on d30 IHD: 24.9%, CRRT 21.8%  subgroup Pt with higher weight gain (FO) CRRT better (HR 0.54, 95% CI 0.29–0.99; p = 0.05). | n.a. | 8/8 | no difference  composite PO: mortality or RRT dependence 30d after RRT onset  At FO: Advantages for CVVH |
| Bell 2007 (20) | RT | 2002 | Sweden | CVVH 944  IHD 158 | IHD vs CRRT *dialysis dependence*  d90: OR 2.13 (95%CI 1.3-3.5) – CRRT Better | good | 7/8 | CRRT better  bias: Group size 6:1 |
| Bonnassieux 2018 (24) | RT | 25.750 | ICU french database | CRRT 40.905  IHD 17.730 | *renal recovery* 86.2%. IHD had less *recovery* at hospital discharge; OR 0.910 (95% CI, 0.834-0.992) p = 0.0327. | good | good | CRRT better  retrospectively large French database  better recovery under CRRT |
| Forest 2023 (225) | Posthoc | 2196 | START-AKI | CVVH 1590  IHD 606 | composite *mortality* & RRT dependent:  -OR 0.81 (95%CI 0.66-0.99) p=0.044  RRT *dependency*  d90:  -0.61 (95%CI 0.39-0.94) (no p value)  *mortality* d90:  -OR 0.90 (95%CI 0.74-1.11) (no p value) | 3/6  critical |  | better *recovery* (higher proportion of composite EP)  bias: no randomization for question  bias: modality was chosen individually.  bias: group sizes not equal to approx. 3:1 |
| Naorungroj 2022 (141) | Posthoc | 2542 | ATN *Trial* | 2175 CRRT  367 IHD | -among survivors, CRRT reduced 28-d RRT *dependence* (OR, 0.54 [95% CI 0.37–0.80]; p = 0.002)  -more RRT-free days (OR: 1.38 [95% CI 1.11–1.71]). | 4/6  critical | good | CRRT better  bias 1: ATN was randomized for standard vs. higher dose. no randomization for the question  bias 2: cont. vs int. collective size very unequal.  bias 3: Regionality. French centers more likely to be IHD and had higher mortality |
| Gaudry 2022 (72) | Posthoc | 543 | IDEAL ICU | 543 CRRT  268 IHD | -no difference: Survival without further need of RRT at d28 CRRT: 117 (43.5%) IHD: 132 (48.0%) RR 0.92 (0.75 - 1.13)  -renal recovery d28 CRRT: 123 (45.7%) IHD: 134 (48.9%) RR 1.00 (0.83-1.21) | 4/6 critical | good | no difference  bias 1: IDEAL ICU was randomized for early vs. late onset. no randomization for the question  bias 2: a large part of the patients were excluded for study according to non-systematic criteria  bias 3: cont vs int. collective size very unequal.  mortality (SOFA >10) better under CRRT  mortality (SOFA <10 IHD better  *Relaxation* equals |
| Koyner 2024 (112) | RT | 3.804 | Premier PINC AI Healthcare Database | 2740 IHD  1064 CRRT  On ICU | -with CRRT less RRT *dependency* 90d (4.9% vs. 7.4% p = 0.006) OR 0.68 (95%CI 0.47–0.97), p = 0.03.  CRRT pat.:  -younger, fewer comorbidities, less CKD  -more admissions in large clinics  -more surgery, sepsis, shock and ventilation  -Change from CRRT to IHD 34.5%  -LOS ICU CRRT Median 17d vs IHD 11d | good | 6/8 | RRT dependence at discharge (26.5% vs. 29.8%, p = 0.04) and at day 90 (4.9% vs. 7.4% p = 0.006), weighted adjusted OR (95% CI): 0.68 (0.47–0.97), p = 0.03. |
| **1 RCT: CONVINT with 252 pat. no difference**  **5 RT: cumulative 32,398 pts analyzed, of which no difference in collectives with 26,388 pts, better recovery at 6010 CRRT, new RT based on database Koyner 2024**  **1 OT: large French cohort**  **3 posthoc: inappropriate randomizations for research question, unequal collectives (more CRRT)**  **Data not uniform, but strong trend towards no effect**  **GRADE ⊕⊕⊝⊝ evidence low**  **little controlled-randomized data – one RCT no difference**  **an observational study - CRRT better**  **most RT no difference**  **posthoc studies with clear bias – only of very limited use.** | | | | | | | | |

## Systematic Reviews: CVVH versus IHD - Outcome hemodynamic stability

| **Study** | **Design** | **N** | **Collective** | **Hemodynamic Stability CRRT vs IHD** | **Notes** |
| --- | --- | --- | --- | --- | --- |
| Rabindranath 2007 (165) | SR | 1550 | 15 RCTs  to 10/2006 | -hemodynamic stability: RR 0.48 (95%CI 0.1-2.28) – only 2 studies with 110 pts  -hypotension: RR0.92 (95%CI 0.72-1.16) – 3 studies 265 patients  -MAP SMD 5.35 (95%CI 1.41-9.29), I2=0%, p=0.01 – 2 studies 56 pat. | CRRT better (little evidence) |
| Pannu 2008 (152) | SR | 3190 pat. RCT  2868 pat OTs | 30 RCTs  8 OT  to 10/2007 | Hypotension RR 0.87 (95%CI 0.68-1.12, I2=0% - 3 studies, 389 Pt | no difference (little evidence) |
| Zhang 2015 (249) | SR | 617 pat. RCT  675 pat. OTs | 7 RCTs  10 OTs  until 2014 | vasopressors equal to p=0.8, I2=0% (2 studies, 89 pat.) | no difference (little evidence) |
| Schoenfelder 2017 (186) | SR | 617 pat. RCT  7682 pat. OT | 9 RCTs  40 OTs  to 12/2014 | hypotension RR 0.71 (95% CI 0.39, 1.31), I275%, p=0.28 | no difference (little evidence)  OTs showed hypotension less frequently with CRRT, RCTs did not find an effect |
| Zhou 2021 (256) | SR | 621 pat. CRRT  1503 pat. IHD  333 pat. PD | 23 RCTs  to 6/2020 | hypotension RR 1.08 (95%CI 0.41-2.85), I2 74% – 2 studies | no difference (low evidence) |
| Russian 2022 (178) | SR |  | 12 RCTs  until | -6 studies CRRT vs. IHD, 3 studies SLED vs CRRT, 2 more  -8/12 RCT no differences, 4/12 RCT CRRT better  -Incidence of hypotensive episodes varied 5-60% | SR with *hemodynamic stability* as PO  no difference detectable (little evidence) |
| **Result** | **6SR** | **Overall, all SRs refer to a few studies that indicate the topic of hemodynamic instability, number of hypotension or MAP (see table below). The evidence is to be assessed as low, as few studies are analyzed with rather few studies.** | | | **Data insufficient**  **GRADE ⊕⊝⊝⊝ evidence very low** |

## Trials: CVVH versus IHD - Outcome hemodynamic stability

| **Study** | **Design** | **N** | **Hypotension** | **MAP mmHg** | **Hemodynamic stability** | **ROB**  **critical** | **US** | **Notes** |
| --- | --- | --- | --- | --- | --- | --- | --- | --- |
| John 2001 John 2001 (99) | RCT | 10 IHD  20 CVVH |  | after 2h:  IHD 15 ±17 vs CVVH 12 ±19, p<0.05 | Norepinephrine after 2h:  IHD: 2.16 ±1.78  CVVH 2.10 ±1.98 n.s. | 3/6 | n.a. | CVVH better MAP |
| Augustine 2004 (12) | RCT | 40 IHD  40 CVVHDF |  | IHD 75.0 ±13 vs. CVVHDF 77.4 ±8.1 n.s. | vasopressors  IHD 55%, CVVHDF 52.5% | 2/6  critical |  | no difference |
| Kielstein 2004 (105) | RCT | 20 SLED  19 CVVH |  | according to 6d:  SLED 72.9 ±3  CVVH 78.9 ±3 | after 12h μg/kg/h  SLED 0.45 ±0.12  CVVH 0.42 ±0.13 n.s. | 3/6 critical |  | no difference |
| Uehlinger 2005 (212) | RCT | 55 IHD  70 CVVHD |  | d5 IHD 74±2 vs. 76 ±2, the so-called "D-Shirt". | Norepinephrine  IHD 3300; CVVHDF 4300 µg/12h, p=0.21 | 2/6 critical |  | no difference |
| Vinsonneau 2006 (220) | RCT | 184 IHD  175 CVVHDF | IHD 39%, CVVHDF 35% n.s. |  |  | 2/6 critical |  | no difference |
| Schwenger 2012 (187) | RCT | 115 SLED  117 CVVH |  | IHD 118.3 ±17.1  CVVH 124.3 ±15.6 | vasopressors not different | 2/6 critical |  | no difference |
| Badawy 2013 (14) | RCT | 40 SLED  40 CVVHDF |  | After 24h:  IHD 127 ±10  CVVHDF 120 ±11 | vasopressors not different | 3/6 critical |  | no difference |
| Schefold 2014 (182) | RCT | 128 IHD  122 CVVH |  | d1: IHD 73.3 ±16.5 vs. CVVH 72.0 ±14.1 MAP, n.s. | Epinephrine: IHD: 0.7, CVVH 0.6g, p=0.96  Norepinephrine: IHD: 19.1, CVVH 18.5g, P=0.30 | 1/6  critical | n.a. | no difference |
| **Result** | **8 RCT** |  | **Smaller cohorts, hemodynamic stability usually secondary outcome, GRADE ⊕⊝⊝⊝ evidence very low** | | | | | |

## Trials evidence table: Outcome SAE - Thrombocytopenia and Transfusion Frequency

| **Study** | **Design** | **N** | **Cohort** | **RRT Method** | **Outcome** | **ROB** | **US** | **Notes** |
| --- | --- | --- | --- | --- | --- | --- | --- | --- |
| Vinsonneau 2006 (220) | RCT | 360 | ICU patients with multi-organ failure | CVVHDF vs IHD | Thrombocytopenia (< 50,000 L) under IHD 12%, under CVVHDF 18%, n.s. | 2/6 |  | protocol amendment after 8 months due to low recruitment, exclusion of patients with platelets < 30,000/μL and bleeding |
| Schwenger 2012 (187) | RCT | 232 | ICU patients with AKI, 54% with sepsis | CVVH versus 12 h SLEDD | Platelet count under NET:  SLEDD 157.200, CVVH 158.500; n.s.  Total amount of transfused RBC units (ml)  SLED 1.375 ml vs. CVVH 1976 (p<0.019) | 2/6 |  | only one value for platelets given under therapy |
| Pschowski 2015 (163) | posthoc | 252 | ICU patients with AKI | CVVH versus IHD | max. platelet drop up to d3:  IHD 7,2% vs. CVVH 11,6% (n.s.)  Transfusion at least 5 RBC units by day 7:  Number of patients IHD 23% vs. CVVH 26% (n.s.)  RRT Caused Blood Loss (System Clotting): IHD 30% vs. CVVH 57.4% (p<0.01)  transfused RBC units up to day 7:  IHD 7,7 vs. CVVH 7,3 (n.s.) | 3/6 |  | secondary analysis of the Covint study |
| Vives 2022 (221) | RT | 351 | cardiac surgery | CVVH/CVVHDF versus SLED (8-10 h) | Transfusion of > 5 RBC units  Number pat. CVVH/CVVHDF 45,9% vs SLED 75,9% (n.s.) |  | 5/9 | monocentric retrospective observational study from Canada |
| **Result** | **3 RCT**  **1 RT** | **1.195** | **Thrombocytopenia and transfusion frequency were not the primary outcome parameters in any study. The data density is sometimes low (e.g. Schwenger 2015). The results cannot be transferred to the current time, as no study used regional citrate anticoagulation.**  **GRADE ⊕⊝⊝⊝ evidence very low** | | | | |  |

## PRISMA Chart: Intracranial Pressure in continuous versus Intermittent Renal Replacement Procedures

Records identified through PubMed searching

(n = 117)

**Screening**

**Included**

**Eligibility**

**Identification**

Records identified through Cochrane searching

(n = 85)

Records after duplicates removed

(n = 283)

Records screened

(n = 77)

Pediatric, off topic, unfinished studies excluded (n = 206)

Full-text articles assessed for eligibility (n = 14)

observational (n = 5)

retrospective (n = 8)

RCTs

(n = 1)

Systematic Reviews (n = 0)

Cochrane Review (n = 0)

Records identified through Scopus searching

(n = 92)

off topic studies excluded

(n=63)

## Trials: Intracranial Pressure in continuous versus Intermittent Renal Replacement Procedures

| **Study** | **Design** | **N** | **Cohort** | **RRT mode** | **Outcome** | **ROB**  **critical** | **US**  **fulfilled** | **Notes** |
| --- | --- | --- | --- | --- | --- | --- | --- | --- |
| Davenport 1989 (47) | RCT | 7 | hepatic encephalopathy | IHF vs CAVHF | ICP IHF: 8.4 ±1.5 to 12.6±1.8 mmHg  ICP CAVH: 15.6 ±5.2 to 11.7±2.3 mmHg | 3/6 |  |  |
| Davenport 1993 (46) | OT | 30 | hepatic encephalopathy | IHF vs CVVH | ICP IHF: const.  ICP CAVH: 15.6 ±5.2 to 11.7±2.3 mmHg |  | 4/9 | MAP drop in the first hour at HF with a reduction in CPP 35 ±8% |
| Ronco 1999 (177) | OT | 12 | only AKI with RRT | IHD vs CVVH | IHD: gray matter 52.3 ±5.2 - 38.9 ±5.3 white matter of 36.7 ±3.5 - 24.8 ±3.2 Hounsfield units, avg. Delta ‐26.7% |  | 4/9 | CVVH const |
| Wu 2013 (233) | OT | 10 | ICB | SLED vs CVVH | but no difference between method p=0.46 |  | 4/9 | ICP increase in both methods,  Approx. 0.1% for CVVH, approx. 0.4% for SLED in the first 4 hours |
| Johansen 2017 (98) | OT | 12 | CKD patients | lowflux-IHD vs  pre-IHDF | MRI: no differences between procedures; gray matter: HD: r2 = 0.83; HDF: r2 = 0.73; white matter: r2 = 0.02; HDF: r2 = 0.004 |  | 9/9 | *total brain volume*  IHD: +1.8 ± 1.7% (18.7 ± 17.4mL)  Pre-IHDF: +2.0 ± 0.9% (22.3 ± 10.7 mL) |
| Venkatasubba Rao 2018 (218) | OT | 14 | CKD in general | IHD | Osmolarity medium -6.4 mOsm/L  (SD 6.6) from pre- to post-dialysis,  *VIPS edema index* (E-Dex) +9.7% (SD 12.9) p = 0.037). |  | 7/9 | *volumetric Integral Phase-shift*  *Spectroscopy* (VIPS) to evaluate fluid shifts under HD: Osmo drops about 6-7 mmosmol/l during IHD |
| Cardoso 2018 (31) | RT | 1186 | hepatic encephalopathy | 61 (18%) CVVH  59 (17%) IHD  220 (65%) no RRT | mortality 21d:  CVVH: OR 0.47 [95%CI 0.26–0.82] decreased  IHD: OR 1.68 [95%CI, 1.04–2.72] increased |  | 7/9 | ammonia reduction:  CVVH: -38%, IHD: -23%, no RRT:-19% |
| Fletcher 2010 (67) | RT | 4 | 3 SHT |  | one patient ICP increase under IHD, then stable under CRRT, others stable ICP under CRRT |  | 2/9 |  |
| Zhou 2011 (255) | RT | 16 | heat stroke | CVVHF with 25-30° filtrate | core temperature reduced from 41.3 ± 0.2°C to 38.7 ± 0.1°C after 2 h and 36.7 ± 0.1°C after 5 h during CVVH (p < 0.05) |  | 6/9 |  |
| Kawarazaki 2013 (104) | RT | 5 | neurological damage |  | 5/343 patients with RRT had neurological damage, 5 at RRT, of which none recovered early, but 2 died |  | 4/9 | retrospective evaluation of a poor outcome in RRT and AKI 5/343 |
| Nadkarni 2015 (139) | RT | 6.074 | ICB, stroke  More RRT |  | *cerebral infarction*: RRT had OR 1.30; 95% 95%CI 1.12–1.48; *P<0.001)*  *ICB*: RRT OR 1.95; CI95% 1.61–2.36; *P<0.01)* |  | 9/9 | database analysis, AKI with RRT significantly increases mortality in stroke and ICB |
| Osgood 2015 (146) | RT | 4 | cerebral edema | 2 CVVH  2 IHD | temporal association of cerebral entrapment immediately due to RRT |  | n.a. | 2 cases of parallel drop of the S-Na |
| Alqahtani 2019 (7) | RT | 13.642 | stroke + RRT/CKD | IHD | in-hospital *mortality* was higher in the dialysis group (7.6% versus 5.2%, P<0.001), |  | n.a. | database analysis in CKD patients with cerebral infarctions |
| Choi 2020 (39) | RT | 223 KDIGO3:  115 +RRT  108 –RRT | resuscitation + AKI/RRT with temperature  management | 111 CVVH  4 IHD | RRT group significantly lower 6-month *mortality than* non-RRT group (93/115 [81%] vs. 98/108 [91%], P = 0.04).  OR mortality 6 months 0.569 (95%CI 0.377-0.857, p=0.01) |  | n.a. | *targeted temperature* TTM (33 or 36 °C),  Duration of the TTM (24 or 48 h) |
| **Cohorts small, heterogeneous. 1 RCT with a lower degree of quality and procedures that are no longer used in Dt today. Nevertheless, studies consistently conclude that IHD leads to more increases in intracranial pressure than CRRT**  **1 Study SLED: no difference to CVVH. 1 Study compares diffusion and convection: no difference**  **GRADE ⊕⊝⊝⊝ evidence very low** | | | | | | | | |

## Systematic Reviews: Fluid Overload

| **Study** | **Design** | **Studies** | **N** | **RRT mode** | **Outcome** | **Notes** |
| --- | --- | --- | --- | --- | --- | --- |
| Al Dalbhi 2021 (6) | SR | 4 RCT | 411 | SLED 205  CVVH 206 | SMD -0.24 95%CI -0.72-0.24  Tau² = 0.18; Chi² = 14.14, I² = 79%  Total Effect: Z = 0.99 (P = 0.32) | no significant differences |
| Zhang 2015 (249) | SR | 4 RCT  3 RT | RCT 367 Pt  RT 140 Pt | SLED  CVVH | 4 RCTs: -0.1 (-0.39-0.19) L/d mean diff.  3 OTs -0.06 (-1.03-0.91) L/d mean diff. | no significant difference in fluid removal of SLED vs CVVHD |
| Zhou 2021 (256) | SR | 3 Studies  7 Studies | 1519 Pt | IHD vs CVVH  SLED vs CVVH | +70 (-64-204) ml/d difference  -151 (-328-27) ml/d difference | no significant differences |
| **Result** |  |  | **3 SR do not calculate differences**  **GRADE ⊕⊝⊝⊝ evidence very low** | | | |

## Trials evidence table: Fluid Overload

| **Study** | **Design** | **N** | **Cohort** | **RRT Method** | **Outcome** | **ROB**  **critical** | **US** | **Notes** |
| --- | --- | --- | --- | --- | --- | --- | --- | --- |
| Mehta 2001 (136) | RCT | 166 | AKI with RRT  4 Medical ICU | 82 IHD  84 CVVH | UF goals not achieved:  -IHD at 28.8%  -CVVH at 9% | 2/6 |  | Net UF not specified,  Figures reported *data not shown* |
| John 2001 (99) | RCT | 33 | AKI with RRT  1 Medical ICU | 10 IHD  22 CVVH | IHD -1520 ±440 ml  CVVH -1680 ±360 ml n.s. | 2/6 |  | technically no blinding possible |
| Augustine 2004 (12) | RCT | 80 | AKI with RRT  1 Medical ICU | 40 CVVH  40 IHD | CVVH -4.005 (-13.533 - +6.745) ml/3d  IHD +1.535 (-5.510 - +9.125) ml/3d | 3/6 |  | CRRT higher net UF after 72h, then no more difference |
| Uehlinger 2005 (213) | RCT | 125 | AKI with RRT  1 Medical ICU | 55 IHD  70 CVVHDF | Avg. Fluid balance:  -IHD 230 ±1264 ml/12h  -CVVHDF 211 ±1351ml/12h (P=0.66) | 2/6 |  | technically no blinding possible |
| Vinsonneau 2006 (220) | RCT | 360 | AKI with RRT  22 Medical ICU | 184 IHD  175 CVVHDF | IHD 2213 (2141–2285) ml/d net UF  CVVHDF 2107 (2011–2203) ml/d Net UF | 2/6 |  | technically no blinding possible |
| Bouchard 2009 (27) | OT  PICARD | 618 | AKI with RRT  5 Medical ICU | ~26% IHD only  ~22% only CVVH  ~5% first IHD, than CVVH  ~12% CVVH than IHD | still FO with end CRRT 8%  still FO with end IHD about 18% |  | 9/9 | OR 30d mortality was associated with more FO 2.52 (95% CI 1.55–4.08)  🡪 CVVH less FO at KRT end |
| Schefold 2014 (182) | RCT  COVINT | 252 | AKI with RRT  1 Medical ICU | 129 IHD  123 CVVH | IHD 20.5 ±23.2L Fluid Balance  CVVH 24.9 ±28.4L Fluid Balance p=0.19 | 2/6 |  | technically no blinding possible |
| **6 RCT**  **1 OT** | | | | **No blinding possible**  **GRADE ⊕⊝⊝⊝ evidence very low** | | | | |

## PRISMA Chart: Liver Failure

Records identified through PubMed searching

(n = 221)

**Screening**

**Included**

**Eligibility**

**Identification**

Records identified through Cochrane searching

(n = 16)

Records after duplicates removed

(n = 256)

Records screened

(n = 34)

Pediatric, off topic, unfinished studies excluded (n = 222)

Full-text articles assessed for eligibility (n = 6)

Non-controlled trials

(observational n = 3)

(retrospective = 2)

RCTs

(n = 0)

Systematic Reviews (n = 1)

Cochrane Review (n = 0)

Records identified through Scopus searching

(n = 81)

Pediatric, off topic, unfinished studies excluded (n = 28)

## Trials: Liver Failure

| **Study** | **Design** | **N** | **Cohort** | **RRT mode** | **Outcome** | **ROB**  **critical** | **US** | **Notes** |
| --- | --- | --- | --- | --- | --- | --- | --- | --- |
| Davenport 1989 (47) | OT | 7 | ALF | 4 IHF  3 CAVHF | IHF: ICP 8.4 ± 1.5 🡪 12.6 ±1.8 mmHg (p <0.05)  CAVHF 15.6 ± 5.2 🡪 11.7 ±2.3 mmHg |  | 4/9 | better ICP stability under CAVHF |
| Davenport 1993 (46) | OT | 30 | hepatic encephalopathy | IHF vs CVVH | ICP IHF: IHF: ICP of 45 +/- 5% in 1h IHF.  ICP CAVH: 15.6 ±5.2 to 11.7±2.3 mmHg |  | 4/9 | MAP waste in the first hour at IHF with a reduction in CPP 35 ±8% |
| Naka 2006 (140) | RT | 40 | 40 ALF  42 no ALF | CVVH | pH 7.40 (7.34, 7.44) vs 7.41 (7.35, 7.44) n.s.  HCO3 (mmol/L) 26.8 (22.4, 28.7) vs 23.8 (18.1, 25.8) <0.005  lactate (mmol/L) 3.1 (2.5, 4.1) vs 4.8 (3.6, 8.9) <0.0005  Chloride (mmol/L) 100 (99, 102) 96 (93, 100) <0.0001 |  | 8/9 | acidification of hyperlactaemia was reduced by hypochloremia (96 vs. 100 mmol/L; p<0.0001) with a high *strong ion difference* (SIDa)  (43.6 vs. 41.9 mEq/L; p<0.05) |
| Cardoso 2018 (31) | RT | 1186 | hepatic encephalopathy | 61 (18%) CVVH  59 (17%) IHD  220 (65%) no RRT | Death21d:  CVVH: OR 0.47 [95%CI 0.26–0.82] decreased  IHD: OR 1.68 [95%CI, 1.04–2.72] increased |  | 8/9 | ammonia reduction:  CVVH: -38%, IHD: -23%, no RRT:-19% |
| Fisher 2022 (65) | OT | 12 | hyperammonemia  4 ALF  8 AOCLF | CVVHF  CVVHD  CVVHDF | CVVH: 27 (IQR 23-32) mL/min  CVVHD: 21 (IQR 17-28) mL/min  CVVHDF: 20 (IQR 14-28) mL/min, p = 0.32. |  | 5/9 | Urea Clr 50 (47-54) mL/min  Creatinin 42 (IQR 38-46) mL/min Ammoniak 25 (IQR 18-29) mL/min,  p = 0.0001. |
| **Result** | **3 OT**  **2 RT** |  | **GRADE ⊕⊝⊝⊝ evidence very low** | | | | | |

# Anticoagulation of Renal Replacement Therapy

PICO Questions

|  | 1 – RCA vs HSA |
| --- | --- |
| P | In critically ill patients with AKI and RRT *without therapeutic anticoagulation* |
| I | Does the use of RCA improve outcomes |
| C | compared to systemic heparin anticoagulation? |
| O | *mortality*, *recovery* from RRT, filter patency, bleeding (blood loss, transfusions),  SAE (hypo- or hypercalcemia, metabolic acidosis or alkalosis) |

|  | 2 – Are LMWH better than LMWH? |
| --- | --- |
| P | In critically ill patients with AKI and RRT *without therapeutic AK* |
| I | Does the use of low molecular weight Heparins improve outcomes |
| C | compared to unfractionated heparin? |
| O | *mortality*, *recovery* from RRT, filter patency, bleeding (blood loss, transfusions),  SAE (hypo- or hypercalcemia, metabolic acidosis or alkalosis) |

|  | 3 – Can Argatroban be used as a systemic AK instead of Heparin |
| --- | --- |
| P | In critically ill patients with AKI and RRT *with therapeutic anticoagulation (Heparin, Argatroban) due to HIT2* |
| I | Does the use of Argatroban improve outcomes |
| C | compared to low molecular weight heparins |
| O | *mortality*, filter patency, bleeding (blood loss, transfusions), filter service life |

PIO Questions

|  | 4 - Liver failure |
| --- | --- |
| P | Are certain patient groups (liver failure, lactic acidosis) of critically ill patients with AKI and RRT |
| I | at risk when using RCA? |
| O | mortality, renal recovery, SAE/AE |

|  | 5 - Citrate accumulation |
| --- | --- |
| P | In critically ill patients with AKI and RRT |
| I | is citrate accumulation an indicator of increased mortality? (Surrogate parameter?) |
| O | mortality, renal recovery, SAE/AE |

PICO Search Terms

| **Patient** | **Intervention** | **Comparison** | **Outcome** |
| --- | --- | --- | --- |
| Critical care, Critically ill, intensive care | citrate | Heparin | *mortality* |
| Acute kidney injury, Acute renal failure | Anticoagulation | Nadroparin, Enoxaparin, Dalteparin, Certoparin, Tinzaparin  Low molecular weight Heparin | Duration of RRT |

Database Search string 1 (RCA)

*(renal replacement therapy OR dialysis OR hemofiltration OR hemodiafiltration)*

AND (acute kidney injury OR acute renal failure)

AND (critical care OR intensive care OR critically ill)

AND NOT[TI] pediatric OR neonates OR children

AND (citrate OR Heparin OR anticoagulation OR patency OR clotting OR clogging)

*Database Search string 2 (low molecular weight heparins)*

(renal replacement therapy OR dialysis OR hemofiltration OR hemodiafiltration)

AND (critical care OR intensive care OR critically ill)

AND (Enoxaparin OR Nadroparin OR Dalteparin OR certoparin Or (low molecular weight Heparin))

*Database Search string 3 (Argatroban)*

(renal replacement therapy OR dialysis OR hemofiltration OR hemodiafiltration)

AND (critical care OR intensive care OR critically ill)

AND (Argatroban OR HIT2 OR Thrombin inhibition)

## PRISMA Chart 1: RCA and UFH

Records identified through PubMed searching

(n = 257)

**Screening**

**Included**

**Eligibility**

**Identification**

Records identified through Cochrane searching

(n = 106)

Records after duplicates removed

(n = 652)

Records screened

(n =209)

Off topics (n = 443)

Full-text articles assessed for eligibility

(n = 140)

Retrospective (n = 34)

Observational (n= 45)

Records identified through Scopus searching

(n = 354)

RCTs (n = 48)

Systematic Reviews (n = 13)

Pediatrics, protocols, Reviews (n = 69)

## PRISMA Chart 2: LMWH

**Screening**

**Included**

**Eligibility**

**Identification**

Records identified through Cochrane searching

(n = 36)

Records after duplicates removed

(n = 124)

Records screened

(n =38)

Off topics (n =86)

Full-text articles assessed for eligibility

(n = 20)

Retrospective (n = 4)

Observational (n= 7)

Records identified through Scopus searching

(n = 95)

RCTs (n = 6)

Systematic Reviews

(n=3)

Pediatric, *Trial* protocols, other topics (n = 18)

Records identified through PubMed searching

(n = 36)

## PRISMA Chart 3: Argatroban

**Screening**

**Included**

**Eligibility**

**Identification**

Records identified through Cochrane searching (n =22)

Records after duplicates removed

(n =83)

Records screened

(n =59)

Off topics (n =24)

Full-text articles assessed for eligibility

(n =8)

Retrospective (n = 1)

Observational (n=2)

Records identified through Scopus searching

(n =21)

RCTs (n = 3)

Systematic Reviews

(n = 2)

Pediatric, *Trial* protocols, other topics (n= 51)

Records identified through PubMed searching

(n =40)

## Systematic Reviews und AMSTAR2

## Synopsis Systematic Reviews: Citrate

| **Study** | **SR**  **AMSTAR** | **Studies** | **Search by** | **Patients** | **Groups** | **Note** |
| --- | --- | --- | --- | --- | --- | --- |
| Liao 2013 (122) | SR  moderate | 4 | Nov 2011 | all | citrate vs. UFH |  |
| Bai 2015 (18) | SR  good | 11 | Apr 15 | all | citrate vs. UFH |  |
| Liu 2016 (124) | SR  good | 14 | Sep 2015 | all | citrate vs. UFH, citrate vs. UFH + Protamine |  |
| Zhang 2019 (251) | SR  moderate | 20 | Jan 2019 | VAT | citrate vs. UFH | hepatic insufficiency, no anticoagulation vs. Heparin |
| Tsujimoto 2020 (210) | Cochrane  good | 8* | Sep 2019 | all | different comparisons | Cochrane Review with studies until 12.09.2019, but for question citrate vs UFH only 8 studies. |
| Chang 2021 (33) | SR moderate | 10 | July 2020 | all | citrate vs. UFH | by Dec 2020, in the end only 10 studies included, focus only on coagulation parameters |
| Raina 2022 (166) | SR  moderate | 24 | 2020 | <18 J | citrate, Heparin, Prostacyclin | pediatric |
| Li 2022 Read 2022 (117) | SR  moderate | 13 | Sep 2021 | all | citrate vs. UFH |  |
| Qi 2023 (164) | SR  moderate | 14 | Sep 2022 | VAT | citrate vs. UFH | liver failure |
| Peng 2023 (157) | SR  good | 19 | Mar 2022 | VAT | citrate vs other AK | liver failure |
| Qi 2023 (164) | SR  moderate | 14 | Sep 2022 | VAT | citrate vs. UFH | liver failure |
| Jacobs 2023 (89) | SR  good | 12 | Feb 2022 | all | citrate vs. UFH |  |
| Christmas 2024 (144) | Cochrane  good | 113 | Nov 2023 | CKD | citrate vs. UFH | Cochrane Report, RCA in chronic intermittent dialysis |
| **Result** | **11 SR**  **2 Cochrane** |  | **Nov 2023**  **Latest** |  |  | **4 SR to RCA in liver failure**  **9 SR to RCA to Outcomes** |

## Systematic Reviews: Citrate vs Heparin mortality

| **Study** |  |  | **28-day mortality** | | | **90-day mortality** | | |  |
| --- | --- | --- | --- | --- | --- | --- | --- | --- | --- |
|  | **Groups** | **Patients** | **Studies** | **N** | **HR** | **Studies** | **N** | **HR** | **Note** |
| Bai 2015 (18) | citrate vs. UFH | all | 5 | 632 | 0.8 [95% CI 0,61; 1,04], p=0,1 |  |  |  |  |
| Liu 2016 (124) | citrate vs. UFH | all | 7 | 879 | 0.97 [95% CI 0,84; 1,13], p=0,72 |  |  |  |  |
| Tsujimoto 2020 (210) | citrate vs. UFH | all | 5 | 462 | 1,06 [95% CI 0,86; 1,30] |  |  |  |  |
| Jacobs 2023 (89) | citrate vs. UFH | all | 6 | 683 | 1,08 [95% CI 0.89–1.31], p=0,91 | 4 | 1014 | 0,9 [95% CI 0.8–1.02], p=0,36 |  |
| Qi 2023 (164) | citrate vs. UFH | VAT | 9 | 212 | RCA: 58.9% (95% CI: 39.2–77.3) Heparin: 47.4% (95% CI: 31.1–63.7), p=0.62 |  |  |  | liver only |
| **Result** |  |  | **5 SR – no difference**  **GRADE ⊕⊕⊕⊝ evidence moderate** | | | **1 SR – no difference**  **GRADE ⊕⊕⊕⊝ evidence moderate** | | |  |

## Systematic Reviews: Renal recovery from AKI

| **Study** | **Groups** | **Cohort** | **Studies** | **N** | **Renal recovery** | **Note** |
| --- | --- | --- | --- | --- | --- | --- |
| Tsujimoto 2020 (210) | citrate vs. UFH | all | 2 | 214 | 1.04 [0.89 , 1.21] |  |
| Jacobs 2023 (89) | citrate vs. UFH | all | 5 | 457 | 1.07 [0.97,1.18]; p=0.176 |  |
| **Result** |  |  | **GRADE ⊕⊕⊝⊝ evidence low** | | | |

## Systematic Reviews : Filter Runtimes

|  |  |  | **System Loss** | | | **Filter loss** | | |  |
| --- | --- | --- | --- | --- | --- | --- | --- | --- | --- |
| **Authors** | **Groups** | **Patients** | **Studies** | **N** | **System Loss** | **Studies** | **N** | **Filter Loss** | **Note** |
| Bai 2015 (18) | citrate vs. UFH | all | 8 | 953 | 1.32 [95% CI 1.02, 1.70], p=0.04 | 6 | 1475 | 1.43 [95% CI 1.02, 2.00], p=0.04 |  |
| Liu 2016 (124) | citrate vs. UFH | all | 12 | 1351 | 15.69 [95% CI 9.3, 22.08], p<0.00001 | 0 |  |  |  |
| Tsujimoto 2020 (210) | citrate vs. UFH | all | 3 | 211 | 1.44 [95% CI 1.10 , 1.87] |  |  |  | only 3 studies included |
| Jacobs 2023 (89) | citrate vs. UFH | all |  |  |  | 12 | 1924 | 14.52 [95% CI 7.22, 21.83] | only filter loss is described, it can be assumed that system losses is also meant. |
| **Result** |  |  |  |  |  |  | **GRADE ⊕⊕⊕⊕ evidence good** | | |

## Systematic Reviews: Bleeding

|  |  |  | **Bleeding** | | | **Transfusions** | | |  |
| --- | --- | --- | --- | --- | --- | --- | --- | --- | --- |
| **Authors** | **Groups** | **Cohort** | **Studies** | **N** | **Bleeding** | **Studies** | **N** | **Transfusion** | **Note** |
| Bai 2015 (18) | citrate vs. UFH | all | 9 | 760 | 0.36 [95% CI 0.21, 0.60] |  |  |  |  |
| Liu 2016 (124) | citrate vs. UFH | all | 10 | 810 | 0.31(95% CI 0.19, 0.51), p<0.0001 |  |  |  |  |
| Tsujimoto 2020 (210) | citrate vs. UFH | all | 7 | 702 | 0.22 [95% CI 0.08, 0.62] |  |  |  |  |
| Jacobs 2023 (89) | citrate vs. UFH | all | 12 | 1627 | 0.32 [95% CI 0.22, 0.47], p<0.00001 | 8 | 1409 | 1.02 [95% CI 0.93, 1.12], p=0.644 |  |
| **Result** |  |  | **GRADE ⊕⊕⊕⊝ evidence moderate** | | | **GRADE ⊕⊕⊕⊝ evidence moderate** | | |  |

## Systematic Reviews evidence table: Hypocalcemia

|  |  |  | **Hypocalcemia** | | | **Hypercalcemia** | | |  |
| --- | --- | --- | --- | --- | --- | --- | --- | --- | --- |
| **Authors** | **Groups** | **Patient groups** | **Studies** | **N** | **Statistics** | **Studies** | **N** | **Statistics** | **Note** |
| Bai 2015 (18) | citrate vs. UFH | all | 7 | 710 | 4.26 [95% CI 1.69, 10.73], p=0.002 | n.a. | n.a. |  |  |
| Liu 2016 (124) | citrate vs. UFH | all | 7 | 621 | 3.96 [95% CI 1.5, 10.43], p=0.005 | good | good |  |  |
| Tsujimoto 2020 (210) | citrate vs. UFH | all | 5 | 400 | 4.51 [95% CI 1.31, 15.55], p=0.02 | 2 | 158 | Unpredictable |  |
| Jacobs 2023 (89) | citrate vs. UFH | all | 9 | 1269 | 3.81 [95% CI 1.67, 8.66], p=0.001 | 3 | 404 | 1.8 [95% CI 0.22, 14.43], p=0.91 |  |
| **Result** |  |  | **More episodes of hypocalcemia with RCA**  **GRADE ⊕⊕⊕⊕ evidence good** | | | **No differences**  **GRADE ⊕⊕⊕⊝ evidence moderate** | | |  |

## Systematic Reviews: Acid/Base Complications

|  |  |  | **Metabolic alkalosis** | | | **Metabolic acidosis** | | |  |
| --- | --- | --- | --- | --- | --- | --- | --- | --- | --- |
| **Authors** | **Groups** | **Cohort** | **Studies** | **N** | **Statistics** | **Studies** | **N** | **Statistics** | **Note** |
| Bai 2015 (18) | citrate vs. UFH | all | 6 | 540 | 1.39 [95% CI 0.4, 4.85], p=0.6 | - |  |  |  |
| Liu 2016 (124) | citrate vs. UFH | all | 7 | 590 | 0.84 [95% CI 0.47, 1.49], p=0.55 | - |  |  |  |
| Tsujimoto 2020 (210) | citrate vs. UFH | all | 5 | 369 | 2.88 [95% CI 1.12, 7.39], p=0.03 | - |  |  | Metabolic Disorder = pH > 7.5 |
| Jacobs 2023 (89) | citrate vs. UFH | all | 8 | 1208 | 0.82 [95% CI 0.45, 1.52], p=0.16 | 7 | 1369 | 1.71 [95% CI 0.99, 2.93], p=0.76 |  |
| **Result** |  |  | **No difference**  **GRADE ⊕⊕⊕⊕ evidence good** | | | **No difference**  **GRADE ⊕⊕⊕⊕ evidence good** | | |  |

## Systematic Reviews: Thrombocytopenia

|  |  |  | **HIT2** | | | **Thrombocytopenia** | | |  |
| --- | --- | --- | --- | --- | --- | --- | --- | --- | --- |
| **Authors** | **Groups** | **Cohort** | **Studies** | **N** | **Statistics** | **Studies** | **N** | **Statistics** | **Note** |
| Bai 2015 (18) | citrate vs. UFH | all | 5 | 824 | 0.46 [95% CI 0.21, 1.01], p=0.05 | - |  |  |  |
| Liu 2016 (124) | citrate vs. UFH | all | 5 | 824 | 0.41 [95% CI 0.19, 0.87], p=0.02 | - |  |  |  |
| Tsujimoto 2020 (210) | citrate vs. UFH | all | - |  |  | 3 | 412 | 0.39 [95% CI 0.14, 1.03], p=0.06 |  |
| Jacobs 2023 (89) | citrate vs. UFH | all | 7 | 1459 | 0.62 [95% CI 0.33, 1.15], p=0.126 | - |  |  |  |
| **Result** |  |  | **Under RCA less HIT2**  **GRADE ⊕⊕⊕⊕ evidence good** | | | **Fewer thrombocytopenia under RCA**  **GRADE ⊕⊕⊕⊝ evidence moderate** | | |  |

## Systematic Reviews: RCA in liver failure

| **Authors** | **Studies** | **Groups** | **Mortality** | **Citrate accumulation** | **Bleedings** | **SAE** | **Note** |
| --- | --- | --- | --- | --- | --- | --- | --- |
| Zhang 2019 (251) | 20 | Citrate vs.  UFH, no AK | RCA: 58.9% (95% CI: 39.2–77.3)  SHA: 47.4% (95% CI: 31.1–63.7), | 12% [95% CI 3%, 22%] | 5% [95% CI 2%, 8%] | increase: pH, HCO3, BE Alkalose, iCa, tCa, totCa/ionCa | no significant increase: S-citrates (MD − 65.82 [95% CI −194.19, 62.55]), lactate (MD 0.49 [95% CI − 0.27, 1.26]) and total bilirubin (MD 0.79 [− 0.70, 2.29]) at CRRT end |
| Qi 2023 (164) | 9 | citrate |  | 5.3% (95% CI 0%–25.3%) |  | -Acidosis 26.4% (95% CI: 0–76.9),  -Alkalosis 1.8% (95% CI: 0–6.8), |  |
| Peng 2023 (157) | 19 | citrate  vs other AK | 42.2% (95% CI 0.272-0.579) p<0.001 | 6.7% [95%CI 1.5-14.4], | 4.6% [95%CI (0.7-11.0)], | totCa/ionCa  aPTT), pH, BE increased | Filter Clotting, 4.4% [95%CI (1.6-8.3)],  *Total random effects* |
| **Result** |  | **RCA in liver failure:**  **mortality – GRADE ⊕⊕⊝⊝ evidence low**  **SAE (citrate Accumulation) - GRADE ⊕⊕⊝⊝ evidence Low** | | | | |  |

## Systematic Reviews: LMWH versus UFH

| **Authors** | **AMSTAR** | **Interventions** | **Studies** | **Cohort** | **Filter runtime** | **Bleeding** | **SAE** | **Note** |
| --- | --- | --- | --- | --- | --- | --- | --- | --- |
| Tsujimoto 2020 (210) | good | LMWH vs. UFH | 5 | all | only Garces 2010  see evidence tab. 10 | no difference (n=233)  RR 0.58 [0.13-2.58], p=0.47 | thrombocytopenia no difference (n=61)  RR 0.57 [0.20-1.63], p=0.29 | Cochrane review with studies until 12.09.2019. no statement on mortality possible (only 1 RCT with 29 patients) |
| Natale 2024 (144) | Good | LMWH vs. UFH | 6 | CKD | no difference (n=91), RR 1.58[0.46-5.42], p=0.47 | - |  | Cochrane Report, RCA in Chronic Intermittent Dialysis |
| Zhou 2023 (257) | good | LMWH vs. UFH | 6 |  |  |  |  | *anticoagulation ranking SUCRA* (surface under the cumulative ranking curve), from best to worst  RCA + LMWH 85.5%,  RCA 67.2%,  NM 59.7%,  LMWH 51.9%,  no 35.5%,  UFH 23.1%, |
| **Result** |  |  | **comparison LMWH and UFH (SR):**  **filter runtime: no difference GRADE ⊕⊝⊝⊝ evidence very low**  **bleeding: no difference GRADE ⊕⊝⊝⊝ evidence very low** | | | | |  |

## Trials: Further studies LMWH versus UFH

| **Authors** | **Design** | **n** | **Intervention** | **Filter runtime** | **Bleeding** | **SAE** | **Note** |
| --- | --- | --- | --- | --- | --- | --- | --- |
| Garcés 2010 (71) | RCT | 40 | CVVHD/ICU  Enoxaparin vs. UFH: | 43±15 vs. 52 ±18h, p=0.10 | 26 vs 0% p=0.018 |  | More bleeding with enoxaprine |
| Oudemans-Van Straaten 2009 (147) | OT | 28 | FXa activity |  |  |  | Hemostasiological analysis under 2 postdilution CVVH strategies |
| Arnold 2020 (10) | RT | 71 | SLEDD with UFH, LMWH, ARG | SLEDD filter runtimes:  UFH 8.11±1.3h  LMWH 11.8±0.5  ARG 8.0±0.9h |  |  |  |
| Joannidis 2007 (97) | RCT | 40 | CVVH, UFH vs. Enoxaparin | UFH: 21.7 h ±16.9h LMWH: 30.6 h ±25.3), p = 0.017 | UFH: 1/20  LWWH: 1/20 |  | Longer filter life under enoxaparin |
| Reeves 1999 (169) | RCT | 47 | CVVH, UFH vs. dalteparin | UFH: 46.8 (5.03) h. LMWH: 51.7 (7.51) h [CI 13 to 23 h]  n.s. | RBC units Volume Applied: UFH: 309 (128) mL  LMWH: 290 (87) mL (p = .90 | Reduction in platelet count: UFH: 63 (25.8) LMWH: 41.8 (26.6) x 10(9)  (p = .57, | 46.8 (5.03) h. Für Heparin, K‐M mean (SE) time to failure war 51.7 (7.51) h. Der 95% CI mean time to failure war ‐13 to 23 h. |
| **Result** |  |  | **comparison LMWH and UFH (further trials):**  **filter runtime: longer with Enoxaparin GRADE ⊕⊝⊝⊝ evidence very low**  **bleeding: more bleeding with Enoxaparin GRADE ⊕⊝⊝⊝ evidence very low**  **SAE: more thrombocytopenia GRADE ⊕⊝⊝⊝ evidence very low** | | | |  |

## Systematic Reviews: Argatroban

| **Authors** | **Interventions** | **Studies** | **Cohort** | **Filter runtime** | **Bleeding** | **SAE: HIT2** | **Note** |
| --- | --- | --- | --- | --- | --- | --- | --- |
| Tsujimoto 2020 (210) |  |  | all | - | - | - | no comparative studies on Argatroban and UFH/citrate |
| Jacobs 2023 (89) | RCA vs UFH | 7 | all | - | - | RCA only trend better RR 0.62 [0.33-1.15] p=0.76 | HIT rate was low, 4.17% in the Heparin group, *relative risk reduction* 20%. Argatroban not treated |
| Natale 2024 (144) | Argatroban vs. UFH | 1 | CKD | - | only: Shi 2008  s. evidence Report 12 | - | Cochrane Report, anticoagulation in chronic intermittent dialysis. |
| **Result** |  |  | **SR, no sufficient data can be shown for Argatroban compared to RCA or UFH**  **Result: Argatroban can be used technically in kidney replacement, outcome data are largely lacking**  **GRADE Bleeding ⊕⊝⊝⊝ evidence very low**  **GRADE filter running times ⊕⊝⊝⊝ evidence very low** | | | | |

## Trials: Further studies Argatroban

| **Authors** | **Design** | **N** | **Intervention** | **Filter runtime** | **Bleedings** | **SAE** | **Note** |
| --- | --- | --- | --- | --- | --- | --- | --- |
| Reddy 2005 (168) | RT | 47 | ARG at HIT2 and CVVH  50 treatment courses, CVVH und IHD |  | 3/50 (6%) | none |  |
| Shi 2008 (189) | RCT | 104 | ARG vs UFH  IHD (CKD pat.) |  | RR 1.92 [95% CI 0.62-6.00] less bleeding under UHF |  |  |
| Link 2009 (123) | OT | 30 | ARG at HIT2 with CVVH | 2% in the first 24 hours | 2/40 minor bleeding |  | dose-finding study |
| Sun 2011 (203) | RCT | 101 | IHF at AKI:  ARG vs. no AK (initial filter flushed with Heparin) | 0 vs 16.9% | none | no difference |  |
| Klingele 2014 (109) | RT | 94 | CVVH cardiac surgery  41 ARG+26 UHF to ARG switch vs 27 UFH |  | RBC Units  1.2±0.3 vs 1.0±0.1 p=0.32 | HIT2 7% below ARG, 15% below UFH | ARG collective had higher SAPSII 52±2 vs43±3 |
| Arnold 2020 (10) | RT | 71 | SLEDD with UFH, LMWH, ARG | SLEDD filter runtimes:  UFH 8.11±1.3h  LMWH 11.8±0.5  ARG 8.0±0.9h |  |  |  |
| **Result** | **2 RCT**  **1 OT**  **3 RT** |  | **In further trials (RCT, OT, RT) no sufficient outcome data for Argatroban compared to RCA or UFH can be shown**  **Result: Argatroban can be used technically in kidney replacement**  **GRADE ⊕⊝⊝⊝ evidence very low** | | | | |

# Dose of Renal Replacement Therapy

PICO Questions

|  | 1 – CVVH: high vs standard dose |
| --- | --- |
| P | In critically ill patients with AKI and RRT |
| I | Does the use of HIGHdose (KONT) (>25 ml/kg/h) lead to improved outcomes |
| C | compared to STDdose (25 ml/kg/h)? |
| O | mortality, recovery from the AKI, more SAE |

|  | 2 – IHD: high vs standard dose |
| --- | --- |
| P | In critically ill patients with AKI and RRT |
| I | Does the use of HIGHdose (daily IHD) lead to improved outcomes |
| C | compared to STDdose (3/week)? |
| O | mortality, recovery from the AKI, more SAE? |

|  | 3 – CVVH: high-volume vs standard dose |
| --- | --- |
| P | In critically ill patients with AKI and RRT and sepsis |
| I | Does the use of high-volume hemofiltration lead to improved outcomes |
| C | compared to standard volume? |
| O | mortality, recovery from the AKI, more SAE |

PIO Questions

|  | 4 - Overdialysis |
| --- | --- |
| P | In critically ill patients with AKI and RRT |
| I | Is hypophosphatemia a surrogate parameter for overdialysis? |
| O | SAE |

PICO Search Terms

| **Patient** | **Intervention** | **Comparison** | **Outcome** |
| --- | --- | --- | --- |
| Critical care, critically ill  intensive care | Dose | Standard of care | *renal recovery* |
|  | High volume |  | Duration of RRT |
| Acute kidney injury  Acute renal failure | intensity  intensive |  | *mortality* |
| Renal replacement therapy | increased |  |  |
| Dialysis, Hemofiltration, hemodiafiltration |  |  |  |

Database Search string

(renal replacement therapy OR dialysis OR hemofiltration OR hemodiafiltration)

AND (acute kidney injury OR acute renal failure)

AND (critical care OR intensive care OR critically ill)

AND NOT[TI] pediatric OR neonates OR children

AND (high volume OR dose OR intensity OR intensive OR increased)

## PRISMA Chart Dose

Records identified through PubMed searching

(n =363)

**Screening**

**Included**

**Eligibility**

**Identification**

Records identified through Cochrane searching

(n = 450)

Records after duplicates removed

(n = 863)

Records screened

(n = 200)

*Trial* protocols, pediatric, comments, editorials excluded

(n = 119)

Full-text articles assessed for eligibility

(n = 81)

Retrospective (n = 6)

Observational (n= 27)

Records identified through Scopus searching

(n = 248)

RCTs (n = 30)

Systematic Reviews (n = 18)

off-topic (n = 663)

## Systematic Reviews und AMSTAR2 Dose

## Systematic Reviews synopsis: CVVH – Standard vs. high dose

| **Study** | **Studies** | **Mortality** | **Recreation** | **SAE** |
| --- | --- | --- | --- | --- |
| Zhongheng 2010 (254) | 6 RCT | RR 0.91 (95% CI, 0.77, 1.08), p=0.29 | RR 0.95 (95% CI, 0.89, 1.00), p=0.05 | more hypophosphytemia due to Bellomo 2009 and Palevsky 2008, other no data |
| Jun 2010 (100) | 8 RCT | RR 0.89 (95% CI, 0.76, 1.04), p=0.14 | RR 1.12 (95% CI, 0.95-1.31), p=0.81 | n.a. |
| Casey 2010 (32) | 7 RCT | RR 0.88 (95% CI 0.75–1.03) | RR 1.12 (95% CI 0.86–1.46) | n.a. |
| Van Wert 2010 (216) | 12 RCT | RR 0.89 (95% CI, 0.77–1.03) | RR 1.15 (95% CI 0.92–1.44) | n.a. |
| Negash 2011 (145) | 5 RCTs | RR 28d 0.88 (95% CI, 0.70, 1.11), p=0.28 | n.a. | n.a. |
| Borthwick 2013 (25) |  | RR ICU 0.59 (95% CI, 0.19, 1.59)  RR 28d 0.67 (95% CI, 0.22, 2.03) | n.a. | n.a. |
| Fayad 2016 (61) | 6 RCT | RR 30d 0.92 (95% CI 0.80 -1.06); low evidence quality | RR (free of RRT d30) 1.03 (95% CI, 0.96, 1.11), low evidence quality  RR (free of RRT d90) 0.98, 95% CI, 0.94-1.01, moderate evidence quality | Hypophosphatemia: RR 1.21, 95% CI 1.11-1.31; High quality evidence (1 study); AE: RR 1.08, 95% CI 0.73 to 1.61; moderate evidence quality |
| Li 2017 (116) | 8 RCTs | RR 90d 0.90 (95% CI, 0.73, 1.11), p=0.32 | good | good |
| Borthwick 2017 (26) | 4 RCT | RR d90 0.89 (95% CI, 0.60-1.32 low evidence quality | good | good |
| Wang 2018 (228) | 8 RCT | RR 28d 0.93 (95% CI, 0.8-1.09), p=0.4  RR 90d 0.91 (95% CI, 0.74-1.13), p=0.41 | RR (RRT dependency 28d) 1.15 (95% CI 1.0-1.33), p=0.05; RR (RRT dependency 90d) 1.24 (95% CI 0.79-1.95), p=0.35 | good |
| **Result** | **10 SR** | **no difference - GRADE**  **mortality ⊕⊕⊕⊕ evidence good** | **no difference - GRADE**  **renal recovery ⊕⊕⊝⊝ evidence Low** | **no difference - GRADE**  **SAE ⊕⊕⊝⊝ evidence low** |

## Trials: CVVH - Standard vs. high dose

| **Study** | **Design** | **N** | **Definition randomized groups (I: increased, L: low)** | **Primary outcome** | **Mortality** | **Renal recovery** | **Notes** |
| --- | --- | --- | --- | --- | --- | --- | --- |
| Bouman 2002 (28) | RCT, multi | 106 | CVVH  I1: 48.2ml/kg/h within 12h  I2: 19-20ml/kg/h within 12h  L: 19-20ml/kg/h if patients develop an absolute criterion for RRT | survival 28d  renal recovery | survival d28: 74.3% in early high-volume CVVHF, 68.8% in early low-volume CVVHF, and 75.0% in late low-volume CVVHF (p=.80) | all hospital survivors had renal recovery (1 exception) |  |
| Atn 2008 (11) | RCT, multi | 1124 | I: IHD 6x/week (Kt/V 1.2-1.4 per treatment) OR CVVHDF administered dose 35ml/kg/h  L: IHD 3x/week (Kt/V 1.2-1.4 per treatment) OR CVVHDF administered dose 20ml/kg/h | mortality 60d | 53.6% intensive vs. 51.5% less intensive (OR, 1.09; 95% CI, 0.86 to 1.40; P=0.47) | complete recovery: 15.4% intense vs. 18.4% less intense  partial recovery: 8.9% intense vs. 9.0% less intense (P=0.24). | more hypotension and electrolyte disorders in the intensive group |
| Saudan 2006 (181) | RCT, single | 206 | CVVHDF and CVVH  I: prescribed dose 42ml/kg/h  L: prescribed dose 25ml/kg/h | Survival d28 and d90 | D28: 39% vs. 59% (P=0.03) D90: 34% vs. 59% (P=0.0005) | 71 vs. 78% (P=0.62) |  |
| Tolwani 2008 (207) | RCT, single | 200 | CVVHDF  I: prescribed dose 35ml/kg/h  L: prescribed dose 25ml/kg/h | Surviving ICU discharge or d30 | 49% high-dose vs. 56% in standard-dose (OR, 0.75; 95%-CI, 0.43 to 1.32; P = 0.32) | 69% high-dose vs. 80% standard-dose (P=0.29) |  |
| Renal 2009 (170) | RCT, multi | 1465 | CVVHDF  I: prescribed dose 40ml/kg/h  L: prescribed dose 25ml/kg/h | mortality 90d | 44.7% in each group (OR, 1.00; 95%-CI, 0.81 to 1.23; P=0.99) | 6.8% higher-intensity vs. 4.4% lower-intensity, continued to receive RRT (OR, 1.59; 95% CI, 0.86 to 2.92; P=0.14) | hypophosphatemia 65% in higher intensity group vs. 54% lower intensity (P<0.001) |
| **Result** | **8** | **3101** | **Higher dose does not improve survival or renal recovery in critically ill patients with ACI requiring dialysis compared to lower dose (25ml/kg/h). CAVE: differences in modalities, CAVE: differences in the primary objective criteria**  **mortality 30 days after randomization: OR, 0.92 (95% CI, 0.80, 1-06), P=0.26**  **renal recovery (free of CRRT d30): OR, 1.03 (95% CI, 0.96, 1.11), P=0.46** | | | **GRADE**  **mortality ⊕⊕⊕⊝ moderate evidence**  **renal recovery ⊕⊕⊕⊝ moderate evidence** | |

## Trials: Standard vs. high dose in IHD

| **Study** | **Study** | **N** | **Dose** | **Mortality (Int: intensive, Std: standard)** | **Dialysis dependency in survivors** | **ROB** | **Notes** |
| --- | --- | --- | --- | --- | --- | --- | --- |
| Conger 1975 (42) | RCT | 18 | 7/wk vs 2.5/wk | Int 3/8  Standard 8/10 | Int 0/5  Hrs 0/2 | 3/6 | alternating randomization |
| Gillum 1986 (78) | RCT | 34 | 6.1/wk vs 3.1/wk | Int 10/17  Std 8/17 | Int 0/7  Hour 1/9 | 3/6 | Randomization with coin toss |
| Schiffl 2002 (183) | RCT | 160 | 6.2/wk vs 3.2/wk | Int 22/80  Std 37/80 | Int 2/58  hrs 0/43 | 3/6 | alternating randomization |
| Faulhaber-Walter 2009 (59) | RCT | 157 | SLED  Urea target <90 vs 120-150 mg/dl | Int 63/81  Std 29/75 | Int 18/45  Std 17/46 | 2/6 | no blinding possible |
| Palevsky 2009 (148) | Posthoc | 1232 | 5.4/wk vs 3.0/wk  kt/V 1.32 ±0.36 | Int 33/108  Std 39/138 | Int 56/75 (74.6%) d60  STD 75/99 (58.0%) D60 | 1/6 | Patient numbers calculated from percentages, therefore calculation without and with this work (with*) |
| **n total** |  |  |  | **Int 98/186-**  **Std 82/182**  **Int 131/310 with***  **Std 121/320** | **Int 20/115**  **Std 18/100**  **Int 76/190 with***  **Std 93/199** |  | |
| **RR total** |  |  |  | **RR 1.08 (95% CI 0.87-1.33) p= 0.677**  **RR 1.12 (95% CI 0.92-1.35) p=0.255 with*** | **RR 0.96 (95% CI 0.54-1.72) P= 0.91**  **RR 0.86 (CI 95% 8.68-1.08) p=0.18 with*** | **no difference**  **GRADE**  **mortality ⊕⊕⊕⊝ moderate evidence**  **Renal recovery ⊕⊕⊕⊝ moderate evidence** | |

## Observational Studies: Standard vs. High Dose in IHD

| **Study** | **Study** | **pat. (n)** | **Dose** | **mortality** | **Dialysis dependence in survivors** | **Notes** |
| --- | --- | --- | --- | --- | --- | --- |
| Abrao 2012 (5) | OT | 121 | kt/V > 5.16 low  kt/V < 5.16 high | Lo 41/61 (67.2%)  Hi 29/60 (48.3%), p=0.055 | good | high kt/V also in control group |
| **Result** |  |  |  |  |  | **no difference**  **GRADE ⊕⊝⊝⊝ very low evidence** |

## Systematic Reviews: Standard vs. high volume dose

| **Study** | **Design** | **Mortality** | **Studies** | **Result** | **AE** | **Notes** |
| --- | --- | --- | --- | --- | --- | --- |
| Borthwick 2013 (25) | 3 RCT | RR ICU 0.59 (95% CI, 0.19, 1.59)  RR 28d 0.67 (95% CI, 0.22, 2.03) | Boussekey 2008, Cole 2001, Ghani 2006, |  |  |  |
| Clark 2014 (40) | 4 RCT | OR d28 0.76 (95% CI, 0.45-1.29), p=0.31 | Boussekey 2008, Sanchez 2010, Zhang 2012, Joannes Boyau 2013 | D28 mortality ns | individual studies show more hypophyphahatemia, more hypothermia |  |
| Borthwick 2017 (26) | 4 RCT  HV | RR d90 0.89 (95% CI, 0.60-1.32), low quality of evidence | Boussekey 2008, Cole 2001, Ghani 2006, Joannes-Boyau 2013 | mortality ns | Individual studies do not indicate AE | High-volume  – until Dec 2015 |
| Junhai 2019 (101) | 21 RCT  HV | mort RR=0.88 (0.81-0.96) P=0.004  APACHE2 -0.93 (-3.35-1.49) p=0.45 | 12 studies China, 1 Malaysia, 6 Europe, 1 Aus/NZ, 1 USA | Better survival  APACHE ns | not reported | Funnel Plot: publication bias  mortality not defined, all mixed  HVHF eliminates more cytokines |
| Yin 2020 (241) | 5 RCT  HV | 28d mort RR 0.96 (0.67-1.38), p=0.4 | Boussekey 2008, Cole 2001, Ghani 2006, Joannes-Boyau 2013, Chung 37 | mortality ns | Individual studies do not indicate AE | until Jun 2019 |
| Huang 2021 (85) | 7 RCT  4 OT  6 RT | mort OR 1.66 (1.36-2.01) p<0.001  MODS 0.94 (0.85-1.04) p=0.25 | 15 studies from China, 1 Lithuania, 1 Japan, 2 Russia with 12-130 pts each, 9 CVVH, 8 HVHF, 1 TPE | better survival  MODS ns | OR 1.62 (1.24-2.11)  P=0.24 | until Jan 2021 |
| **Result** |  |  |  | **mortality - no difference GRADE ⊕⊝⊝⊝ very little evidence**  **SAE – inconsistent GRADE ⊕⊝⊝⊝ very low evidence** | | |

## Trials: Standard vs. high-volume dose

| **Study** | **Design** | **N** | **Definition randomized**  **groups**  **HV: high volume. LV: low volume)** | **Primary outcome** | **Mortality** | **Number of patients** | **Renal recovery** | **ROB** | **Note** |
| --- | --- | --- | --- | --- | --- | --- | --- | --- | --- |
| Ghani 2006 Ghani 2006 (77) | RCT | 33 | HV 6000 ml/h  LV 2000 ml/h | removal of inflammatory mediators | RR 0.90 (0.40-2.02) p=0.79 | HV 6/15  LV 8/18 28-d dead | n.a. | 3/6 |  |
| Boussekey 2008 (29) | RCT, single | 20 | HV: 65ml/kg/h  LV: 35ml/kg/h | vasopressors | Survival d28: P = 0.65 | HV 3/9  LV 6/10 28d Death | n.a. | 3/6 |  |
| Zhang 2012 (250) | RCT, single | 280 | EV: 85ml/kg/h  HV: 50ml/kg/h | Death (all causes) at d28, 60, 90 | Tod 28d: 57.4% vs. 58.3%  Tod 60d: 59.6% vs. 62.6%  Tod 90d: 59.6% vs. 63.3%; P=0.58 |  | Live with RRT at d90: 7% vs. 9.6% | 5/6 |  |
| Joannes-Boyau 2013 (96) (IVORY ROAD) | RCT, mutli | 137 | HV: 70ml/kg/h für 96h  LV: 35ml/kg/h für 96h | mortality 28d | 37.9% vs. 40.8%; 0.93 (0.61-1.41)  P=0.94 | HV 25/66  LV 29/71 28d mortality | n.a. | 5/6 |  |
| Combes 2015 (41) (HEROIC) | RCT, multi | 224 | HV: 80ml/kg/h for 48h, then changeover  LV: not exactly defined, actual dose achieved 18.6% | mortality 30d | 36% vs. 36%, OR, 1.00 (95% CI, 0.58 - 1.73 P=1.00 |  | Live and renal recovery d30:  59% vs. 63%, OR 0.86 (95% CI, 0.50 to 1.47), P=0.55 | 4/6 |  |
| Park 2016 (153) | RCT, multi (2) | 212 | HV HDF 80 ml/kg  LV HDF 40 ml/kg | Patient and kidney survival at d28 and d90 | 28-d mortality HR, 1.02; 95% CI, 0.73-1.43; P=0.9) | HV 69/105  LV 69/107 mortality | 28-d renal survival (HR, 0.96; 95% CI, 0.48-1.93; P=0.9 | 2/6 | mortality and renal recovery equal  Predilution CVVHDF  Il1, IL6 sig. reduced |
| **Result** | **7 RCTs** | **1012** | **high volume renal replacement therapy, defined as >50ml/kg/h, in critically ill AKI patients with sepsis does not improve survival or renal recovery**  **however, it may increase cytokine elimination** | | | **RR 0.98 (0.82-1.17)**  **P=0.79** | **GRADE**  **⊕⊝⊝⊝ very little evidence** | | |

# Pharmacology in Renal Replacement Therapy

PICO Questions

| P | In critically ill patients with AKI and RRT |
| --- | --- |
| I | Improves continuous antibiotic administration |
| C | Compared to prolonged antibiotic administrations |
| O | *mortality*, *recovery* from RRT, hemodynamic stability, SAE (thrombocytopenia, blood loss/transfusion frequency)? |

| P | In critically ill patients with AKI and RRT |
| --- | --- |
| I | Improves the application of a TDM |
| C | Compared to therapy without TDM |
| O | *mortality*, *recovery* from RRT, hemodynamic stability, SAE (thrombocytopenia, blood loss/transfusion frequency) |

PIO Questions

| P | In critically ill patients with AKI and RRT |
| --- | --- |
| I | 1. How should the starting dose of an anti-infective drug be chosen? 2. How to choose the maintenance dose of an anti-infective drug? 3. Is continuous infusion of a time-dependent antibiotic preferred over intermittent infusion? 4. Can the use of therapeutic drug monitoring be recommended? 5. When is RRT helpful for lithium intoxication? 6. When can RRT be considered for the elimination of exogenous substances (drugs, poisons)? |
| O | mortality, *clinical cure* |

PICO Search Terms

| **Patient** | **Intervention** | **Comparison** | **Outcome** |
| --- | --- | --- | --- |
| critical care  critically ill  intensive care | continuous antibiotic administration | prolonged antibiotic administration | *mortality* |
| acute kidney injury  acute renal failure | TDM | no TDM | *clinical cure* |
| renal replacement therapy  dialysis  Hemofiltration  hemodiafiltration |  |  |  |

Database Search string

(renal replacement therapy OR dialysis OR hemofiltration OR hemodiafiltration) AND (critical care OR intensive care OR critically ill) AND ((antiinfective OR antibiotic) AND (pharmacodynamic OR pharmacokinetic) OR (prolonged OR intermittent OR bolus OR continuous) OR (therapeutic drug monitoring))

## PRISMA Chart

Records identified through PubMed searching

(n = 842)

**Screening**

**Included**

**Eligibility**

**Identification**

Records identified through Cochrane searching

(n = 903)

Records after duplicates removed

(n = 944)

Pediatric, no critical care, *Trial* protocols,

off-topic excluded (n = 409)

Full-text articles assessed for eligibility

(n = 516)

SR

n = 36

Records identified through Scopus searching

(n = 289)

Continuous vs intermittend infusion n=222

PK/PD and TDM

n = 258

Records screened

(n = 535)

Non-sytematic Reviews, state of the art articles exluded (n = 19)

## Systematic Reviews synopsis PK/PD 1

**IB**: intermittent bolus, **PI**: prolonged infusion, **CI**: continuous infusion, **PK/PD**: pharmacokinetic/pharmacodynamic

| **Study** | **Anti-Infective** | **Topic** | **Comparison** | **RRT included** | **Result** | **Notes** |
| --- | --- | --- | --- | --- | --- | --- |
| Antonello 2021 (9) | Fosfomycin  17 trials | continuous infusion | Dosage | -- | Loading dose 8g  Maintenance dose 16-24g/d |  |
| Chen 2020 (37) | Meropenem  7 trials  1191 patients | continuous infusion | CI vs IB | -- | CI vs IB:  mortality RR = 0.66, 95% CI 0.46–0.98, p = 0.03)  clinical cure RR = 1.15, 95% CI 1.02–1.30, p = 0.026  microbiological eradication RR 1.20, 95% CI 1.01–1.42, p = 0.04 | CI better than IB, reduced mortality, clinical cure |
| Dhaese 2020 (50) | ß-Lactame  33 trials | extended infusion | PI vs IB | -- | adjusted effect PI vs IB auf  bacterial killing low (coefficient 0.66, 95% confidence interval − 0.78 - 2.11) | IB and PI require different PK/PD targets, no difference in bacterial killing |
| Duceppe 2021 (51) | ß-Lactame  32 trials | ECMO | Dosage | (--) | Effects of ECMO on PK/PD varied depending on the substance | evidence insufficient  PK/PD strongly dependent on other organs |
| El-Haffaf 2021 (56) | Piperacillin/ Tazobactam  10 trials | HP | PK Optimization  CI vs IB | and | lowest piperacillin clearance 3.12 L/h, highest 19.9 L/h. varied between 11.2 - 41.2 L. Tazobactam clearance 5.1-6.78 L/h, and tazobactam Vd 17.5 - 76.1 L. | CI achieved PD goals more reliably |
| Falagas 2013 (58) | Piperacillin/  Tazobactam  Carbapenem  14 trials | continuous infusion | CI vs IB | -- | CI led to mortality RR 0.59, 95%CI 0.41-0.83)  pneumonia: mortality RR 0.50; 95% CI, 0.26–0.96 | CI lower mortality |
| Fawaz 2020 (60) | Piperacillin/ Tazobactam  23 trials  3828 patients | continuous infusion | CI vs IB | -- | clinical cure OR 1.56, 95% CI 1.28-1.90, P = 0 .0001), mortality rates OR 0.68, 95% CI 0.55-0.84, P = 0 .0003) | CI less mortality, more clinical cure |
| Flannery 2020 (66) | Vancomycin  11 trials | continuous infusion | CI vs IB | -- | AKI OR, 0.47; 95% CI, 0.34-0.65  PTA OR 2.63; 95% CI, 1.52-4.57 | 53% reduction in AKI and 2.6x better PTA under CI |

## Systematic Reviews synopsis PK/PD 2

**IB**: intermittent bolus, **PI**: prolonged infusion, **CI**: continuous infusion, **PK/PD**: pharmacokinetic/pharmacodynamic

| **Study** | **Anti-Infective** | **Topic** | **Comparison** | **RRT included** | **Result** | **Notes** |
| --- | --- | --- | --- | --- | --- | --- |
| Hao 2016 (81) | Vancomycin | continuous infusion | AKI Risk | -- | AKI RR=0.61, 95% (CI) 0.47-0.80; P<0.001  treatment failure no difference | less AKI under CI |
| Heidari ,Khalili 2023 (82) | Linezolid  35 trials | PK | PK/PD | yes | dose adjustment recommended in special collectives. Under RRT dose reduction and TDM. |  |
| Hoff 2020 (84) | Review  narrative | RRT modalities | PK/PD | yes | PK and PD under different RRT modalities |  |
| Hui 2022 (86) | Linezolid | continuous infusion | PK/PD | yes | Linezolid requires dose adjustment depending on PK/PD conditions |  |
| Ishikawa 2023 (87) | ß-Lactame  neutropenia  5 trials | continuous infusion | CI vs IB | -- | Clinical failure RR (95% CI 0.74 (0.53, 1.05)  mortality RR 1.25, 95% CI 0.44, 3.54) | no difference in mortality, but shorter fever periods |
| Lee 2018 (115) | ß-Lactame  pneumonia  13 RCTs | continuous infusion | CI vs IB | -- | clinical cure RR 1.177; 95% CI 1.065–1.300) p<0.05  mortality RR 0.845; 95% CI 0.644–1.108 p=ns | CI has better clinical cure rates |
| Liu 2023 (126) | Linezolid  RRT  6 trials | RRT modalities | PK/PD | yes | High variability - Individualized dosing and TDM required under RRT |  |
| Luo 2019 (129) | miscellaneous | prolonged | PI vs IB | -- | mortality RR = 0.77, 95% CI = 0.66-0.89 clinical cure RR = 1.11, 95% CI = 1.04-1.19 | Less mortality and better clinical cure under CI |

## Systematic Reviews synopsis PK/PD 3

**IB**: intermittent bolus, **PI**: prolonged infusion, **CI**: continuous infusion, **PK/PD**: pharmacokinetic/pharmacodynamic

| **Study** | **Anti-Infective** | **Topic** | **Comparison** | **RRT included** | **Result** | **Notes** |
| --- | --- | --- | --- | --- | --- | --- |
| Perrott 2010 (158) | Meropenem  20 trials | prolonged | CI vs IB | -- | PTA under CI and IB comparable, consider smaller doses with shorter intervals |  |
| Roberts 2023 (175) | ß-Lactame  12 SR | narrative Review | Review by SR | -- | evidence for PK/PD still needed | A meta-analysis of existing SR |
| Roberts 2009 (176) | ß-Lactame  14 trials | continuous infusion | PI vs CI | -- | clinical cure (n = 755 patients; OR 1.04, 95% CI 0.74-1.46, p = 0.83  mortality (n = 541 patients; OR 1.00, 95% CI 0.48-2.06, p = 1.00 | no difference between CI and IB for mortality and CC |
| Shiu 2013 (190) | Cochrane  CI vs IB  29 trials | continuous infusion | IB vs CI | -- | all–causes mortality (n = 1241, RR 0.89, 95% CI 0.67 to 1.20, P = 0.45  clinical cure (n = 975, RR 1.00, 95% CI 0.93 to 1.08, P = 0.98 | no difference CI vs IB |
| Sime 2012 (192) | ß-Lactame  158 trials | TDM | TDM vs Std | -- | Clinical trials were still lacking in 2012 | Theoretically sensible, but data are still missing (2012) |
| Steffens 2021 (200) | Meropenem  35 trials | TDM | TDM vs Std | -- | TDM optimized PK/PD from Meropenem | TDM Recommended |
| Thabit 2019 (205) | ß-Lactame  39 trials | Prolonged | PI vs IB | -- | CI can produce better fT > MIC, IB usually a sufficient PTA | TDM had advantages |
| Vardakas 2018 (217) | Antipseudom  ß-Lactame  22 trials | Prolonged | PI vs IB | -- | mortality RR 0·70, 95% CI 0·56-0·87 | PI had less mortality |
| Waineo 2015 (223) | Vancomycin  6 trials | continuous infusion | CI vs IB | -- | CI leads to less AKI | AUC24 suitable for vancomycin dosing |
| Yusuf 2014 (244) | Piperacillin  Tazobactam  5 trials | Prolonged | CI vs IB | -- | PI with less nephrotoxicity  PI or CI for time-dependent antibiotics, esp. for patients with severe infections | PI and CI superior |
| Zhu ,Zhou 2018 (258) | Different  53 trials | Prolonged | CI vs IB | -- | CI superior |  |
| Jamal 2014 (92) | Piperacillin Vancomycin Meropenem  30 trials | RRT and PK/PD |  | + | correlation Effluent Rate and Clear.:  Meropenem (rs = 0.43; p = 0.12), piperacillin (rs = 0.77; p = 0.10)  Vancomycin (rs = 0.90; p = 0.08). Target attainment: Meropenem (89%), Piperacillin (83%), Vancomycin (60%) | Effluent rate was best predictor of antibiotic clearance |
| Abdul-Aziz 2024 (2) | ß-Lactame  18 RCTs | Prolonged | CI vs IB | - | 90d mortality: risk ratio 0.86 (95% CI, 0.72-0.98; I2 = 21.5%  Clinical Cure: risk ratio 1.16; 95% CI, 1.07-1.31; | BLINGIII included: CI better survival and more clinical cure |

## Systematic Reviews continuous vs prolonged antibiotic application: AMSTAR2

## Systematic Reviews: Continuous vs prolonged anti-infective doses

| **Study** | **Trials** | **Anti-Infective** | **Mortality** | **Clinical cure** | **Notes** |
| --- | --- | --- | --- | --- | --- |
| Falagas 2013 (58) | 3 RCT  3 OT  8 RT | Carbapenem  Piperacillin | 0.59 (95% CI 0.41–0.83)  P=0.003 | RR = 1.13 (95% CI 0.99- 1.28)  P=0.08 | CI reduced mortality from carbapenems and piperacillin |
| Roberts 2016 (174) | 3 RCT | ß-Lactame | 30d M: 19.6 vs. 26.3%  RR 0.74 (95% CI 0.56-1.00)  P=0.045 | 55.4 vs 46.3%  RR 1.20 (95% CI 1.03-1.40)  p= 0.021 | Clinical cure = disappearance of all signs and symptoms at 7-14d after end of treatment  CI reduces mortality in ß-lactams |
| Lee 2018 (115) | 13 RCT | ß-Lactame | RR 0.845 (95% CI 0.644-1.108)  P=0.819 | RR 1,177 (95% CI 1.07-1.30)  P=0.121 | Subgroup Sepsis more clinical cure for Meropenem RR 1.194 CI95% 0.46-0.98) 0.072 |
| Yu 2018 (243) | 6 RCT  4 OT | Meropenem | RR 0.66 (95% CI 0.50-0.88)  P=0.004 | OR 2.10 (95% CI 1.31±3.38)  P=0.002 | PI or CI had lower mortality and better clinical cure with meropenem |
| Vardakas 2018 (217) | 14 RCT | ß-anti pseudom.  (Carb, ß-LAct) | RR 0.70 (95% CI 0.56-0.87)  P=0.001 | RR 1.06, 95%CI 0.96–1.17, I2=39%) | CI reduced mortality, no difference in clinical cure |
| Luo 2019 (129) | 30 RCT  5 OT  8RT | antibiotics | RR = 0.77, (95% CI 0.66–0.89) | RR = 1.11, (95% CI 1.04–1.19), | CI or PI reduced mortality and improved clinical cure, if only RCTs were evaluated, then only clinical cure was better. |
| Chen 2020 (37) | 1 RCT  6 OT | Meropenem | RR = 0.66, (95% CI 0.46–0.98)  p = 0.03 | RR = 1.15, (95% CI 1.02–1.30) P=0.026 | CI improved outcome at Meropenem - Mainly OTs evaluated |
| Fawaz 2020 (60) | 10 RCT  2 OT  10 RT | Piperacillin  Tazobactam | OR 0.68 (95% CI 0.55–0.84)  P = 0 .0003 | OR 1.56, (95% CI 1.28–1.90)  P = 0 .0001) | CI or PI reduced mortality and improved clinical cure for piperacillin/tazobactam |
| Wu 2021 Wu 2021 (232) | 18 RCT | ß-Lactame | RR 0.82 (95% CI 0.72-0.94) | RR 1.31 (95% CI 1.15-1.49) | CI or PI reduced mortality and improved clinical cure for ß-lactams |
| Ishikawa 2023 (87) | 3 RCT  2 RT | ß-Lactame | RR 1.25 (95% CI 0.44, 3.54) | RR 0.74 (95% CI 0.53, 1.05)  “Clinical failure” | CI or PI reduced mortality and clinical failure |
| Abdul-Aziz 2024 (2) | 18 RCT | ß-Lactame | RR 0.86 (95% CI, 0.72-0.98; I2 = 21.5%; high certainty | RR 1.16; 95% CI 1.07-1.31; moderate certainty | CI or PI reduced mortality and improves clinical cure for ß-lactams |
| **Result** | **9 SR** |  | **Mortality ⊕⊕⊝⊝** | **Clinical Cure ⊕⊕⊝⊝** | **PK/PD attainment ⊕⊕⊕⊝** |

## Trials: New studies (without RRT) - continuous vs prolonged anti-infective drugs

|  | **Trial** | **Anti-Infective** | **n** | **Mortality** | **Clinical cure** | **ROB**  **critical** | **Notes** |
| --- | --- | --- | --- | --- | --- | --- | --- |
| Dulhunty 2013 (53) (BLING1) | RCT | ß-Lactam | 60 | 90% vs 80%, p=0.47 | 70% vs 43%; P = .037 | 0/6 | CI: MIC achieved in 82% of patients (18 of 22)  IB: MIC achieved in 29% (6 of 21) (P = .001). |
| Dulhunty 2015 (54) (BLING2) | RCT | ß-Lactam | 432 | 74.3% vs 72.5%  HR 0.91 (95% CI 0.63-1.31)  P = 0.61). | 52.4% vs 49.5%  OR 1.12 (95% CI 0.77-1.63)  P = 0.56 | 0/6 | 59/220 (26.8%) had RRT  no difference |
| Abdul-Aziz 2016 (3) (BLISS) | RCT | ß-Lactam | 140 | 14d: HR 0.63 (95%CI 0.32-1.20), p=0.166  30d: HR 0.77 (95%CI 0.43-1.34), p=0.360 | 56 vs 34%, p = 0.011 | 1/6 | RRT was an exclusion criterion  fT/MIC better with CI  CI improved clinical cure, no difference for mortality |
| Monti 2023 (138) (MERCY) | RCT | Meropenem | 607 | 47 vs 49% RR 0.96 (95% CI 0.81-1.13), p 0.60 Composite PO:  mortality + resistance | d28, median (IQR, 0 – 15d) antibiotic-free d:  CI: 3d vs IB: d2, mean difference, 0.4 d 95% CI −0.9 - 1.7d, P = .57 | 0/6 | no difference mortality+resistance endpoint  no difference antibiotics days off |
| Dulhunts 2024 (52) (BLING3) | RCT | ß-Lactam | 7202 | odds ratio, 0.91 (95%CI 0.81 to 1.01), P = .08 | OR 1.26 (1.15 - 1.38) <.001 | 0/6 | no difference in mortality, better clinical cure |
| **Result** | **6 RCT** | | **6221** | **mortality: no difference**  **GRADE ⊕⊕⊕⊝**  **moderate highlight** | **clinical cure:**  **GRADE ⊕⊕⊕⊝**  **Moderate highlight** | **new RCTs with good study quality** | |

## Trials: New studies (with RRT) - continuous vs prolonged anti-infective doses

| **Study** | **Trial** | **Anti-Infective** | **n** | **RRT** | **Clinical cure**  **Cont. vs Bolus Gifts** | **HP/PD**  **target attainment (TA)**  **Cont. vs Bolus Gifts** | **ROB**  **Critical** | **US**  **reached** | **Notes** |
| --- | --- | --- | --- | --- | --- | --- | --- | --- | --- |
| Jamal 2015 (90) | RCT | Meropenem | 16 | CVVH | na. | TA (2 mg/l) >4x 100%t:  100% for CI and IB | 2/6 | -- | CI better steady state  IB higher peak concentrations |
| Jamal 2015 (91) | RCT | Pip | 16 | CVVH | na. | TA (16 mg/l) >4x 100%t  87.5 vs 62.5% | 2/6 | -- | CI higher TA |
| Langgartner 2008 (113) | RCT | Meropenem | 6 | CVVHDF | na. | 227 vs 233 mg/l/h AUC | 2/6 | -- | AUC comparable |
| Dulhunty  2015 | RCT | ß-Lactam | 432 | not defined | 74.3% vs 72.5%  HR 0.91 (0.63-1.31) P = 0.61). | 52.4% vs 49.5%  OR 1.12 (0.77-1.63)  P = 0.56 | 0/6 | -- | 59/220 (26.8%) had RRT  no RRT subgroup analysis |
| Shotwell 2016 (191) | OT | Pip/Taz | 68 | CVVH, CVVHDF |  | At 6g/d <45% TA At 9g/d >95% TA  (TA: 4x 50%t) | -- | 4/9 | CI was associated with better PTA, Effluent rates influence PTA |
| Philpott 2019 (159) | OT | Cefepime | 10 | CVVH, CVVHD |  | All achieved MIC8x 100%t | -- | 6/9 | no comparison group |
| Awissi 2015 (13) | OT | Pip/Taz | 20 | HDF | -- | 90% reached 64 mg/L with prolonged administration | -- | 5/9 | 4h Pip/Taz Infusion  SC = 0.809, Clr 65.8 ml/min |
| Judges 2019 (172) | RT | Pip/Taz | 484 | CVVHD, IHD |  | Clearance fraud:  -no AKI 6.8 L/h  -CVVHD 4.3 l/h  -IHD 2.6 L/h (median) | -- | 7/9 | RRT Increased Pip/Taz Clearance  TDM required |
| **Result** |  |  |  |  | **data available under RRT low – GRADE:**  **clinical cure ⊕⊝⊝⊝ evidence very low**  **target attainment ⊕⊝⊝⊝ evidence very low** | | **Under CI, a better target attainment was achieved**  **RRT procedures change clearance**  **Outcomes not significant** | | |

## Systematic Reviews AMSTAR: TDM

|  | 1. PICO components | 2. Protocol before evaluation | 3. Study design explanation | 4. Comprehensive search strategy | 5. Duplicate study selection | 6. Duplicate data extraction | 7. Details of excluded studies | 8. Description of included studies | 9a. Risk of Bias (RCTs) | 10. Funding sources of RCTs | 11. MA: statistical methods | 12. MA: ROB impact assessed | 13. ROB impact evaluated | 14. Heterogeneity | 15. Publication bias | 16. Reports conflicts of interest | **Overall rating of quality** |
| --- | --- | --- | --- | --- | --- | --- | --- | --- | --- | --- | --- | --- | --- | --- | --- | --- | --- |
| Sime 2012 |  |  |  |  |  |  |  |  |  |  |  |  |  |  |  |  | poor |
| Steffens 2021 |  |  |  |  |  |  |  |  |  |  |  |  |  |  |  |  | poor |
| Abdulla 2022 |  |  |  |  |  |  |  |  |  |  |  |  |  |  |  |  | poor |
| Sanz-Codina 2023 |  |  |  |  |  |  |  |  |  |  |  |  |  |  |  |  | good |
| Matusik 2022 |  |  |  |  |  |  |  |  |  |  |  |  |  |  |  |  | good |
| Luxton 2022 |  |  |  |  |  |  |  |  |  |  |  |  |  |  |  |  | good |

## Systematic Reviews: TDM in AKI and RRT

| **Study** | **until** | **Anti-Infective** | **Number of studies** | **Pharmacokinetic target attainment** | **Clinical cure** | **Notes** |
| --- | --- | --- | --- | --- | --- | --- |
| Sime 2012 (192) | 12/2020 | ß-Lactam | 158 trials | na. | na. | no validated PK/PD targets |
| Steffens 2021 (200) | 1/2021 | Meropenem | 35 trials | na. | na. | TDM particularly helpful for AKI |
| Abdulla 2022 (4) | 4/2021 | ß-Lactame  TDM | 6 RCT | na. | na. | TDM often not available  PK/PD goals different |
| Sanz-Codina 2023 (180) | 11/2022 | different | 10 RCT | PTA: RR = 1.41 (95% CI 1.13-1.76) | mortality RR=0.86; 95% CI, 0.71e1.05)  Therapy Failure: RR = 0.70 (95% CI 0.54-0.92) | reduced nephrotoxicity (RR = 0.55 (95% CI, 0.31-0.97) |
| Matusik 2022b (133) | 2021 | different | 139 trials | na. | na. | highly variable TAs |
| Luxton 2022 (130) | 6/2020 | Penicilline | 3 RCT  16 OT  9 case reports | insufficient data | insufficient data | no meta-analysis possible |
| **Result** |  |  |  | **Insufficient data**  **GRADE Target Attainment ⊕⊝⊝⊝ evidence very low**  **GRADE Clinical Cure ⊕⊝⊝⊝ evidence very low** | | |

## Trials: TDM in AKI and RRT

| **Study** |  | **Anti-Infective** | **N** | **Number of samples** | **Procedure** | **Dose adjustment**  **PK Target attainment (PTA)** | **Other outcomes** |  |
| --- | --- | --- | --- | --- | --- | --- | --- | --- |
| Connor 2011 (43) | OT | Pip | 19 | good | TDM in CVVH Effluat |  | Piperacillin levels in dialysate correlated with serum levels |  |
| Saved 2017 (55) | RT | div | 76 | 111 | TDM in CVVH | 35% Changes  24% too high, 11% too low levels |  | most Pt. had greatly increased levels |
| Hail 2022 (79) (TARGET) | RCT | Pip/Taz | 254 | 1179 | cont and bolus ±TDM | Target attainment better with TDM (37.3% vs. 14.6%, OR 4.5, CI 95%, 2.9–6.9, p < 0.001). | 1- SOFA with TDM (7.9 points; 95% CI 7.1–8.7) vs without TDM (8.2 points; 95% CI 7.5–9.0) (p = 0.39).  2-TDM 28d mortality (21.6% vs. 25.8%, RR 0.8, 95% CI 0.5–1.3, p = 0.44)  3-clinical cure (OR 1.9; 95% CI 0.5–6.2, p = 0.30  4- microbiological cure (OR 2.4; 95% CI 0.7–7.4, p= 0.12), ns | SOFA=PO, no difference between TDM and non-TDM group  approx. 1/4 RRT |
| Judges 2019 (172) | RT | Pip/Taz | 484 | 933 | TDM | Initial dose: PTA in 34.3%, no PTA 10.1% -minimum PK target of ≥ 33 mg/L reaches in 89.9% (contains 30.2% harmful levels of SC ≥ 100 mg/L). | no AKI/no RRT CL(PIP) 6.8/6.3 L/h (median/IQR)  CVVHD CL(PIP) 4.3/2.6 L/h  IHD CL(PIP) 2.6/2.3 L/h | TDM improves TA, especially in AKI and RRT |
| Roberts 2012 (173) | OT | Cipro  Mero  Pip/Taz | 24 | na. | TDM am CVVH | 5% min PTA not reached; 40% higher PTA not achieved; 10% too high PTA | no difference for intensity of RRT |  |
| **Result** |  |  |  |  |  |  | **TDM on RRT**  **PK/PD attainment GRADE ⊕⊝⊝⊝ evidence very low** | |

## Trials: Lithium

|  | **Study** | **Procedure** | **Clearance** | **Distance mmol/h** | **HWZ** | **Notes** |
| --- | --- | --- | --- | --- | --- | --- |
| Van Bommel 2000 (214) | CR | HV-CVVH | 54 ml/min HV-CVVH | 4.45 mmol/h | n.a. | 4.45 HV-CVVH (vs. 2.47 CVVHDF 2.42 CVVHD |
| Fish, Pobes 2001 (155) | CR | CVVHD  High flux | n.a. | 0.36-0.51 mmol/h,  Dialysat Lithium 2.48 mmol/L | n.a. |  |
| Eyer 2006 (57) | RT  n=15 | IHD vs.  diuretics | 160 +/-15 mL/min with IHD  15 +/-9 mL/min by diuretics | n.a. | 3.5 +/-0.8h 1. HD  29 +/-14h Diuretics | 22 lithium cases, 15 with IHD |
| Oct. 2009 (135) | CR | IHD  CVVHDF | IHD 173 ml/min  CVVHDF 61 ml/min | n.a. | n.a. | first IHD, then CHHHDF to suppress rebound |
| Lopez 2012 (127) | RT  N=14 | IHD | n.a. | n.a. | n.a. | clinical cases described |
| Schmidt 2014 (184) | CR | IHD  SLED | IHD 177.4 ml/min (start)  SLED 129.3 (3h value) | 71.5% | n.a. | first IHD, then SLED to suppress rebound |
| Decker 2015 (48) | SR  N=228 | IHD  CVVH | mean IHD 106.9 (40-180) ml/min CVVH mean 43.1 (19-64) ml/min | n.a. | n.a. | SR and international guideline |
| Lavonas 2015 (114) | Cochrane | -- | n.a. | n.a. | n.a. | no RCTs found, therefore no analysis performed; however, bibliography lists up to 2014 |
| King 2019 (107) | narrative Review | -- | n.a. | n.a. | n.a. | narrative review on RRT in intoxication |
| Spatula 2023 (195) | narrative Review | IHD  CVVH | endogenous GFR 30-40 ml/min  IHD GFR 40-180 ml/min  CVVH GFR 28-62 ml/min | n.a. | good | start HD >4 mmol/l  end HD <1 mmol/l |
| Arnold 2020 (10) | RT  n=12 | 9 IHD, 2 CVVHD  1 IHD+CVVHD | good | good | good | 91 pts with lithium intox., 72 with Li 1.5-2.5,  19 Pat with Li >2.5 mmol/l  literature |
| **Result** |  |  | **GRADE ⊕⊝⊝⊝ evidence very low** | | |  |

# Stopping Renal Replacement Therapy

PICO Questions

| P | In critically patients with AKI and RRT |
| --- | --- |
| I | Do loop diuretics lead to more successful weaning and improved outcome |
| C | compared to no diuretics |
| O | mortality, Weaning Success |

| P | In critically patients with AKI and RRT |
| --- | --- |
| I | Do protocol-based procedures lead to more successful weaning and an improved outcome |
| C | compared to standard therapy |
| O | mortality, Weaning Success |

| P | In critically ill patients with AKI and RRT |
| --- | --- |
| I | Can the *amount of diuresis* (I) indicate *the end of RRT* |
| C | *better than a)* creatinine iS *and b) crea in the urine as a concentration marker and c) the new biomarkers (NGAL, TIMP2/IGFB2 and others)*? |
| O | mortality, Weaning Success |

PIO questions

| P | In critically ill patients with AKI and RRT |
| --- | --- |
| I | Which individualized RRT is supportive for a successful RRT liberation?  What parameters should indicate the continuation of RRT (fluid overload, hyperkalemia, acidosis)? |
| O | mortality, Weaning Success |

PICO Search Terms

| **Patient** | **Intervention** | **Comparison** | **Outcome** |
| --- | --- | --- | --- |
| critical care, critically ill  intensive care | discontinuation  weaning  liberation  stop, stopping  cessation  resolution  *Recreation*  termination | standard of care | mortality  weaning success |
| acute kidney injury  acute renal failure |
| renal replacement therapy  dialysis  Hemofiltration  hemodiafiltration |

Database Search string

(renal replacement therapy OR dialysis OR hemofiltration OR hemodiafiltration)

AND (acute kidney injury OR acute renal failure)

AND (critical care OR intensive care OR critically ill)

AND NOT[TI] pediatric OR neonates OR children

AND (discontinuation OR weaning OR liberation OR stop OR cessation OR termination OR resolution OR stopping OR *recovery*)

## PRISMA Chart

Records identified through PubMed searching

(n = 481)

**Screening**

**Included**

**Eligibility**

**Identification**

Records identified through Cochrane searching (n = 174)

Records after duplicates removed

(n = 852)

Records screened

(n = 120)

Pediatric, no critical care, *Trial* protocols, off-topic excluded

(n=732)

Full-text articles assessed for eligibility

(n = 61)

Retrospective (n = 25)

Observational (n= 23)

Non-systematic Reviews, state of the art articles excluded (n = 59)

Records identified through Scopus searching (n = 324)

RCTs (n = 5

Posthoc (n= 5)

Systematic Reviews (n = 3)

## Systematic Reviews und AMSTAR2

## Evidence table: Definition for successful weaning

| **Studies** | **Definition of Successful Weaning - Weaning Success (WS)** | **Notes** |
| --- | --- | --- |
| Anmiort 2016 | 7d |  |
| Baeg 2020 | 7d |  |
| Cantarovic 2004 | Spontaneous decrease in urea and creatinine for 2 consecutive days without RRT |  |
| Chen 2020 | Hospital discharge |  |
| Chen 2019 | 7d |  |
| Fröhlich 2012 | 7d |  |
| Heise 2012 | 12 p.m. |  |
| Ihara 2019 | Hospital discharge |  |
| Itenov 2018 | 5d & Crea max. 1.5x to baseline |  |
| Jeon 2018 | 3d |  |
| Katayama 2016 | 7d |  |
| Kim 2018a | 14d |  |
| Liu 2021 | 3d |  |
| Ohara 2022 | 10d |  |
| Pan 2022 | 90d | long term analysis |
| Pattharanitima 2021 | no RRT 7d for hospital discharge |  |
| Raurich 2018 | 12 p.m. | based on *Heise 2012* |
| Shawwa 2022 | 90d | long term analysis |
| City 2019 | 2d |  |
| Thomsen 2020 | 3d |  |
| Uchino 2009 | 7d |  |
| Viallet 2106 | 15d |  |
| Groote 2022 | 2d | ELAIN posthoc |
| Wu 2007 | 5d, 30d |  |
| Yoshida 2018 | 7d |  |
| Zhang 2012 | 7d |  |
| **20 studies** | **MW = 5.95 d, 7d: 8/20** |  |

## Trials: Effects of diuretics

Furosemide: Furosemide; W: Weaning

| **Furosemide**  **at RRT end** | **Design** | **N** | **Renal recovery** | **Diuresis** | **Mortality** | **ROB**  **critical** | **US**  **reached** | **Notes** |
| --- | --- | --- | --- | --- | --- | --- | --- | --- |
| Cantarovich 2004 (30) | RCT | 338 | Furosemide 82/107  ctrl. 87/114 | renal recovery  diuresis in 3d postdialysis Period  significantly greater with furosemide (11.5 vs 6.7 L/3 d; P=0.001). | 59/166 with furosemide  50/164 without furosemide p= 0.36 | 0/6 | good | +/-furosemide under RRT.  With furosemide 57%, without furosemide 33% 2L diuresis/d achieved |
| 2009 (215) | RCT | 71 | Furosemide 23/31  ctrl. 24/29  P=0.42 | More Diuresis  247 vs. 117 ml/h (Median) | 36% vs 32% p=0.8 | 1/6 | good | Renal recovery: no difference  mortality no difference  more diuresis under furosemide |
| Uchino 2009 (211) | OT  Posthoc-analysis | 529 | with furosemide more W-Success 44.9 vs 24.9%, p=<0.001 | if 436 ml/d without diuretics or 2330 ml/d with diuretics then 80% chance of a W-success | mortality 28.5% vs. 42.7%, p < 0.0001 |  |  | RO as an indicator of W-success: 0.845 (0.799–0.883) without diuretics, worse with diuretics: 0.671(0.585– 0.750) |
| Heise 2012 (83) | RT | 222 | good | W-Success 66 mL/h vs. 10 mL/h RO in W-Failure | good | good | good | association described:  in WS 50 mg vs 0 mg in weaning failure group furosemide in the first 8h |
| Jeon 2018 (95) | RT | 1176 | with furosemide. more W-Success  OR 5,529 (95%CI 4,120–7,410)  p < 0.001 | more Diuresis  191 ml/d  125 ml/d (oliguric) |  | good | good | in multivariate analysis, diuretics were associated with more WS RO at d1 cutoff: >125 mL/d correlated with W success |
| Raurich 2018 (167) | RT | 86 | W-Success 56.7%  W-Failure.  61.8%, p=0.67 | more diuresis with diuretics 178 ml/6h | In case of successful weaning: less hospital mortality 35.8% vs 57.9%, p=0.08 | good | good | higher AUC 0.94 for Pt with furosemide vs without furosemide AUC 0.85 Implies positive effect for WS with furosemide |
| Yoshida 2019 (242) | RT | 52 | W-Success 66% Diuretics, W-Failure 36% | 1810 ml/d without diuretics  1720 ml/d with diuretics |  | good | good | rather association described:  in WS group, 66% vs 5% in weaning failure group had furosemide |
| Baeg 2021 (15) | RT | 1158 | On furosemide. d1, W-Success: 139 (23.4)  W-Failure 89 (15.8%), p=0.001 | more diuresis with furosemide  581 vs. 275 ml/d | good | good | good | multivariate: RO ≥300 mL d1, and MAP 50∼78 mm Hg, K <4.1 mmol/L, and BUN <35 mg/dL (12.5 mmol/L) d0 predictive of W success  in univariate analysis, diuretics were predictive of W-success |
| Stads 2019 (198) | OT | 92 | Under Diuretics d0–2 W-Success 44 (76) W-Failure 25 (81) p=0.607 |  |  | good | 5/9 | association described:  in W success more furosemide |
| **Result** | **2 RCT**  **2 OT**  **5 RT** |  |  |  | **Renal recovery: GRADE ⊕⊝⊝⊝ evidence very low** | | | |

## Trials: Weaning protocols and scoring systems

| **Study** | **Design** | **Methodology** | **N** | **Variables** | **Renal recovery** | **Mortality** | **Results** | **NOS**  **reached** | **Notes** |
| --- | --- | --- | --- | --- | --- | --- | --- | --- | --- |
| Baeg 2021 (15) | OT | 4 variables with 0-8 points | 1158 | UO  MAP  BUN  K | n.a.  only observation | n.a. | >5 points ROC 0.731  57% sensitivity  81% specificity | n.a. | UO 300 ml on d1  MAP 78 mmHg on d0  BUN 35 mg/dl on d0  K 4.1 mmol/l on d0 |
| Itenov 2018 (88) | OT  posthoc | creatinine  UO  Sex  age | 1328 | predictive value of a score with 4 variables and prediction recovery <25% or >75% likely | n.a.  only observation | n.a. | pat. with calculated <25% recovery chance recovered only 21.5%  Pat with calculated chance >75% recovery: recovered to 81.7% | 7/9 | Prediction model vs. Validation cohort  AUC 73.1  four-field table grouped |
| Mendu 2017 (137) | OT | flow Chart  >500 ml diuresis/d | 176 | standardized clinical assessment algorithms | + | n.a. | RO not sufficient  mostly continued due to FO 69%, uremia 42% | 7/9 | recommended continue 97% followed  followed by 67% on recommendation discontinue |
| Pan 2022a (151) | OT | consensus cluster algorithm | 124 | uNGAL/Crea  SOFA  eGFR | n.a.  only observation | n.a. | 3 clusters calculated via AI with different weaning and mortality outcomes | 7/9 | Artificial intelligence clustering showed clinical parameters in 3 patient clusters with different outcomes |
| Tournament 2019 (208) | RT | protocol based algorithm | 30 | Ziel UO > 0.5 ml/kg/h  MAP >65, SVO2 >70%, Alb >30 g/l | + | n.a. | UO (1.66 vs 1.21 ml/kg/h, p0.02) and SVO2 (93 vs 41%) was better with protocol | 8/9 | loop diuretics + 500 crystalloids  (+ Thiazides)  catecholamines, RBC, Albumin |
| **Result** | **4 OT**  **1 RT** | **N = 2816** |  |  |  |  | **renal recovery GRADE ⊕⊝⊝⊝ evidence very low** | | |

## Trials: Diuresis, creatinine and urea in urine as biomarkers

| **Create i.U**  **at RRT end** | **Design** | **N** | **RO in case of W success or W failure** | **Higher RO**  **Before Weaning**  **W-Success vs W-Failure** | **Creatinine urine**  **Successful vs. not successful** | **Urea in the urine**  **threshold for weaning** | **US**  **reached** | **Notes** |
| --- | --- | --- | --- | --- | --- | --- | --- | --- |
| Aniort 2016 (8) | RT | 67 | + | >8.5 ml/kg/24h threshold | n.a. | > 1.35 mmol/kg/24h | 5/9 |  |
| Frohlich 2012 (69) | RT | 85 | + | 916 +/- 708, vs.  572 +/- 617 | 2h-Cr-Clr:  36 +/- 21, vs. 16 +/- 9 | n.a. | 6/9 |  |
| City 2019 (198) | OT | 92 | + | 2424 ml/24h vs. 1640 ml/24h | Cr-Clr:  29 ml/min vs. 7 ml/min | n.a. | 6/9 |  |
| Thomsen 2020 (206) | OT | 69 | + | 500 ml/24h vs.  100 ml/24h | Cr-Clr:  62 mL/min vs. 43 mL/min | n.a. | 6/9 |  |
| Viallet 2016 (219) | OT | 54 | + | 2400 ml/24h vs. 1900 ml/24h | Cr-Clr:  31 ml/min vs. 18 ml/min | U-your:  187 vs. 86 mmol/24h | 6/9 |  |
| **Result** |  |  |  | **higher urine volume and higher creatinine concentration correlated with weaning success**  **renal recovery GRADE ⊕⊝⊝⊝ evidence Very Low** | | | | |

## Trials: Diuresis minimum quantity

| **Furosemide**  **At RRT outlet** | **UO ml/d**  **Success** | **UO ml/d**  **Failure** | **Cut-off**  **Ml/d** |
| --- | --- | --- | --- |
| Uchino | 1500 +/- 1388 | 180 +/-552 | 400 |
| Heise | 1584 +/-1654 | 240 +/-515 |  |
| Wu | 1893 +/-967 | 190 +/-628 |  |
| Katayama | 1392 +/-1214 | 190 +/-628 | 100 ml/d increase |
| Viallet | 2300 +/-1482 | 1900 +/-1111 | 2575 |
| Happy | 916 +/-708 | 572 +/-617 | 1810 |
| Urban | 2424 +/-1232 | 1640 +/-1217 |  |
| Yoshida | 1990 +/-1008 | 1080 +/-779 | 1720 |
| Jeon |  |  | 191/d |
| Urban | 2424 +/-1232 | 1640 +/-1217 |  |
| Raurich | 1420 +/-1467 | 168 +/-234 | 178/6h (~534ml/24h) |
| Aniort |  |  | 8.6 ml/kg/d |
| Chen |  |  | 715 |
| Romero-Gonzales |  |  | 720 |
| Wu 2007 | 1893 +/-967 | 928 +/-915 | 300 |
| **Range** |  |  | **191 – 2575 (1083 MW)** |

## Trials: Blood biomarkers and successful weaning

|  | **Marker** | **Design** | **N** | **Cut-off** | **Sensitivity** | **Specificity** | **OR** | **AUC** | **Mortality** | **Time** | **P-value** | **US (*)** | **Note.** |
| --- | --- | --- | --- | --- | --- | --- | --- | --- | --- | --- | --- | --- | --- |
| Zhang 2012 (252) | SCys C | RT | 232 | 2.98 mg/L | .81 | .84 | 4.76 (2.83-8.01) | .87 | 39/145 WS  49/87 WF | admission to ICU | <.01 | 9/9 |  |
| Kim 2018 (106) | SCys C | OT | 110 | 1.85 mg/L | .76 | .63 | 0.29 (.12-.71) | .74 | 13/89 14.6%  13/21 61.9% | last RRT | .001 | 9/9 |  |
| Yang 2018 (238) | sCysC | OT | 102 | Cutoff 2,47 mg/L | .95 | .54 | = | .74 | na. | d1 after last RRT-session | .002 | 9/9 | S-creatinine better for prediction |
| Yang 2017 (237) | sCysC | OT | 302 | Cutoff 2.97 mg/L | .8 | .585 | HR 1.52 (1.08-2.2) | .708 | na. | last RRT | .016 | 9/9 | Auch IHD |
| Kim 2016. Abstract In MA Katulka | Cys C-GFR | OT | 112 | 32.9 mL/min/1.73m2 | .65 | .76 | 1.25 | .75 | na. | ? | ? | 9/9 |  |
| Kim 2018 (106) | NGAL | OT | 110 | > 554 μg/L | .91 | .45 |  | .65 | so | last RRT | .029 | 9/9 | NGAL not associated with WS after risk adjustment |
| Chen 2019 (38) | NGAL | OT | 110 | 403 μg/L | .91 | .61 | .995 | .81 | 9/95,  9,5% WS  101/134 75.4% WF p0.001 | start of RRT and last RRT | <.001 | 9/9 | Combination NGAL + RO (≥695 ml/d) in non-septic patients better than NGAL alone. NGAL not suitable for sepsis. |
| Han 2016 (80) | NGAL | RT | 160 | < 456.3 ng/mL | ? | ? | .95 | ? | n.a. | start of RRT | .871 | 9/9 |  |
| Han 2016 (80) | NTproBNP | RT | 160 | < 15,767 pg/mL | = | ? | 0.37 (.178-.787) | .579 | n.a. | start of RRT | .01 | 9/9 | AUC max. for NT-proBNP+CR+UO+ APACHE combination |
| Srisawat 2011a (197) | pNGAL (plasma) | RT | 181 | 257 ng/ml | 125 (ng/ml): .91  257 (ng/ml): .68  393 (ng/ml): .47 | .33  .75  .9 | 2.02 (1.03-3.31) | .74 | 0/93 WS  43/88 48.9%  p0.001 | 1d (RIFLE-F) | .015 | 9/9 | Combination of age, SCr D1, PSI (pneumonia sev. Index), non-renal SOFA score + NGAL = best AUC (.8) |
| Yang 2018 (238) | pNGAL (plasma) | OT | 102 | ? | ? | ? | ? | .661 | so | d1 after last RRT-session | .04 | 9/9 | S-creatinine predicted better than any other biomarker |
| Daniels 2021 (45) | CXCL11 | Post-hoc RCT | 72 | 1st Tertile  2nd Tertile  3rd Tertile | 1  11.51 (2.2-59.6)  11.31 (2.1-60.8) | na. | CRRT | d8 | .004  .005 | 8/9 | * | Daniels 2021 | CXCL11 |
| Daniels 2021 (45) | n.a. 9 | Post-hoc RCT | 72 | 1st Tertile  2nd Tertile  3rd Tertile | 1  14.6 (2.3-92.5)  10.4 (1.8-58.7) | na. | CRRT | d8 after start | .004  .008 | 8/9 | proteonomic Single Factors ** | Daniels 2021 | n.a. 9 |
| By Groote 2022 (222) | PEK A 119-159 | Post-hoc RT | 210 | ≤89 vs. >89 pmol/L | HR 1.83 (1.26-2.67) | 14/  55 WS  18/49 WF, p=0.8 | CRRT | ≤ 48 h before CRRT | .002 | 9/9 | estimated 28D cumul. incidence function of WS from RRT 61% vs. 45%, p = 0.022) | By Groote 2022 | PEK A 119-159 |
| Daniels 2021 (45) | CXCL11 | Post-hoc RCT | 72 | 1st Tertile  2nd Tertile  3rd Tertile | 1  11.51 (2.2-59.6)  11.31 (2.1-60.8) | na. | CRRT | d8 after start | .004  .005 | 8/9 | * | Daniels 2021 | CXCL11 |
| **Result** |  | **4 posthoc, 7 OT, 4 RT** | | |  |  |  |  |  | **GRADE ⊕⊝⊝⊝ evidence very low** | | | |

## Trials: Successful weaning and urine biomarkers

|  | **Biomarker** | **Design** | **N** | **Limits** | **Sensitivity** | **Specificity** | **OR** | **AUC** | **Mortality** | **Time** | **P-value** | **US** | **Notes** |
| --- | --- | --- | --- | --- | --- | --- | --- | --- | --- | --- | --- | --- | --- |
| Thomsen 2020 (206) | uNGAL | OT | 54 | > 1650 ng/mL | 0.86 | 0.73 |  | 0.81 | n.a. | 6 h after completion |  | 9/9 | combination with 24-h RO (<210 ml) better (Sen. 0.79, Spec. 0.91) |
| City 2019a (199) | uNGAL | OT | 90 | 277 vs. 776 ng/mL mean |  |  | 1.00 (95% CI 1.00-1.00) |  | so | d2 after last RRT | 0.724 | 9/9 |  |
| Pan 2022 (150) | uL-FABP /Cr (log) | OT | 140 | ≥ 2.2 mg/g Cr |  |  | HR (Cox): 0.35 uL-FABP ≥ 2,2 mg/g Cr | 0.79 | n.a. | last RRT | 0.01 | 9/9 | composite uL-FABP + SOFA score |
| **Result** |  | **3 OT** |  |  |  |  |  |  |  |  | **GRADE**  **⊕⊝⊝⊝ evidence very low** | | |

## Trials: Successful weaning and fluid overload

|  | **Design** | **N** | **Balance**  **success** | **Balance**  **failure** | **P-value** | **Mortality** | **US**  **reached** | **ROB**  **critical** | **Notes** |
| --- | --- | --- | --- | --- | --- | --- | --- | --- | --- |
| 2009 (215) | RCT | 71 | -169 (958) | -75 (871) ml | 0.25 | 14/55 W success  18/49 W failure, p=0.8 | n.a. | 1/6 | Fluid Balance during the study |
| Dewitte 2015 (49) | OT | 57 | 1910 (1300-2700)  at inclusion | 2670 (1860-3270) | 0.04 | 2/29 7%W success  17/28 50% W failure p=0.001 | 9/9 | n.a. |  |
| Gaião 2016 (70) | OT | 41 | 3600 (1175-8025) | 12,000 (6625-17975 ml) | 0.004 | n.a. | 7/9 | n.a. | very small cohort, selected |
| Chen 2019 (38) | OT | 110 | 11,937.5―14,341.8 | 11,604.8―14,179.7 | 0.95 | 9/95 9.5% W success  101/134 75.4% W failure p=0.001 | 9/9 | n.a. |  |
| City 2019 (198) | OT | 92 | -1.284(2.884) | -1.250 (2942) | <0.001 | 40/161 24.8% W success  26/ 77 33.8% W failure | 6/9 | n.a. | OR 0.734 (95% CI 0.618-0.876) p 0.001 |
| Wu 2017 (231) | RT | 52 | -350 (-528-125) | 850 (220-990) | 0.001 |  | 5/9 | n.a. | only 9 vs 9 patients compared |
| Raurich 2018 (167) | RT | 86 | 100(-3.300-3000) | 4.800(-1000-9.300) | 0.002 | 24/67, 35.8% W success  11/19 57.9% W-Failure p=0.08 | 6/9 | n.a. |  |
| Shawwa 2022 (188) | RT | 854 |  | cumulative fluid balance associated with CRRT termination d7 per L increase 1.03(1.01-106) 0.001 as a predictor of prolonged RRT (>7d) |  | n.a. | 9/9 | n.a. |  |
| Bouchard 2009 (27) | Posthoc | 618 |  | mortality with less FO to RRT end lower (35 vs 56%; P=0.0002). |  | n.a. | 9/9 | n.a. |  |
| **Result** |  |  |  |  |  | **GRADE**  **Renal recovery ⊕⊝⊝⊝ evidence very low** | | | |

# References

1. Abdelsalam M, Demerdash TM, Assem M, Awais M, Shaheen M, Sabri A, Alanany H, Kashgary A, Alsuwaida A (2021): Improvement of clinical outcomes in dialysis: no convincing superiority in dialysis efficacy using hemodiafiltration vs high-flux hemodialysis. Therapeutic apheresis and dialysis 25; 483‐489.

2. Abdul-Aziz MH, Hammond NE, Brett SJ, Cotta MO, De Waele JJ, Devaux A, Di Tanna GL, Dulhunty JM, Elkady H, Eriksson L et al. (2024): Prolonged vs Intermittent Infusions of beta-Lactam Antibiotics in Adults With Sepsis or Septic Shock: A Systematic Review and Meta-Analysis. JAMA 332; 638-648.

3. Abdul-Aziz MH, Sulaiman H, Mat-Nor MB, Rai V, Wong KK, Hasan MS, Abd Rahman AN, Jamal JA, Wallis SC, Lipman J et al. (2016): Beta-Lactam Infusion in Severe Sepsis (BLISS): a prospective, two-centre, open-labelled randomised controlled trial of continuous versus intermittent beta-lactam infusion in critically ill patients with severe sepsis. intensive care medicine 42; 1535‐1545.

4. Abdulla A, Van Den Broek P, Ewoldt TMJ, Muller AE, Endeman H, Koch BCP (2022): Barriers and Facilitators in the Clinical Implementation of Beta-Lactam Therapeutic Drug Monitoring in Critically Ill Patients: A Critical Review. 44; 112-120.

5. Abrao JM, Ponce D, De Brito GA, Balbi AL (2012): Can delivery dialysis dose affect survival of acute kidney injury patients? Ren Fail 34; 964-969.

6. Al Dalbhi S, Alorf R, Alotaibi M, Altheaby A, Alghamdi Y, Ghazal H, Almuzaini H, Negm H (2021): Sustained low efficiency dialysis is non-inferior to continuous renal replacement therapy in critically ill patients with acute kidney injury A comparative meta-analysis. Medicine 100; 1-10.

7. Alqahtani F, Ziada K, Rihal CS, Alkhouli M (2019): Incidence and outcomes of early percutaneous coronary intervention after isolated valve surgery. Catheter Cardiovasc Interv 93; 583-589.

8. Aniort J, Ait Hssain A, Pereira B, Coupez E, Pioche PA, Leroy C, Heng AE, Souweine B, Lautrette A (2016): Daily urinary urea excretion to guide intermittent hemodialysis weaning in critically ill patients. Crit Care 20; 43.

9. Antonello RM, Di Bella S, Maraolo AE, Luzzati R (2021): Fosfomycin in continuous or prolonged infusion for systemic bacterial infections: a systematic review of its dosing regimen proposal from in vitro, in vivo and clinical studies. 40; 1117-1126.

10. Arnold F, Westermann L, Rieg S, Neumann-Haefelin E, Biever PM, Walz G, Kalbhenn J, Tanriver Y (2020): Comparison of different anticoagulation strategies for renal replacement therapy in critically ill patients with COVID-19: a cohort study. BMC Nephrol 21; 486.

11. Atn, Network VNaRFT, Palevsky PM, Zhang JH, O'connor TZ, Chertow GM, Crowley ST, Choudhury D, Finkel K, Kellum JA et al. (2008): Intensity of renal support in critically ill patients with acute kidney injury. N Engl J Med 359; 7-20.

12. Augustine JJ, Sandy D, Seifert TH, Paganini EP (2004): A randomized controlled trial comparing intermittent with continuous dialysis in patients with ARF. Am J Kidney Dis 44; 1000-1007.

13. Awissi DK, Beauchamp A, Hébert E, Lavigne V, Munoz DL, Lebrun G, Savoie M, Fagnan M, Amyot J, Tétreault N et al. (2015): Pharmacokinetics of an extended 4-hour infusion of piperacillin-tazobactam in critically ill patients undergoing continuous renal replacement therapy. Pharmacotherapy 35; 600-607.

14. Badawy SH, Amira R.; Samir, Enas M. (2013): A prospective randomized comparative pilot trial on extended daily dialysis versus continuous venovenous hemodiafiltration in acute kidney injury after cardiac surgery. The Egyptian Journal of Cardiothoracic Anesthesia 7; 69-73.

15. Baeg SI, Jeon J, Yoo H, Na SJ, Kim K, Chung CR, Yang JH, Jeon K, Lee JE, Huh W et al. (2021): A Scoring Model with Simple Clinical Parameters to Predict Successful Discontinuation of continuous Renal Replacement Therapy. Blood Purif 50; 779-789.

16. Bagshaw SM, Berthiaume LR, Delaney A, Bellomo R (2008): continuous versus intermittent renal replacement therapy for critically ill patients with acute kidney injury: a meta-analysis. Crit Care Med 36; 610-617.

17. Bagshaw SM, Wald R, Adhikari NKJ, Bellomo R, Da Costa BR, Dreyfuss D, Du B, Gallagher MP, Gaudry S, Hoste EA et al. (2020): Timing of Initiation of Renal-Replacement Therapy in Acute Kidney Injury. N Engl J Med 383; 240-251.

18. Bai M, Zhou M, He L, Ma F, Li Y, Yu Y, Wang P, Li L, Jing R, Zhao L et al. (2015): citrate versus Heparin anticoagulation for continuous renal replacement therapy: an updated meta-analysis of RCTs. intensive Care Med 41; 2098-2110.

19. Barbar SD, Clere-Jehl R, Bourredjem A, Hernu R, Montini F, Bruyère R, Lebert C, Bohé J, Badie J, Eraldi JP et al. (2018): Timing of Renal-Replacement Therapy in Patients with Acute Kidney Injury and Sepsis. N Engl J Med 379; 1431-1442.

20. Bell M, Swing, Granath F, Schon S, Ekbom A, Martling CR (2007): continuous renal replacement therapy is associated with less chronic renal failure than intermittent haemodialysis after acute renal failure. intensive Care Med 33; 773-780.

21. Besen BaMP, Romano TG, Mendes PV, Gallo CA, Zampieri FG, Nassar AP, Jr., Park M (2019): Early Versus Late Initiation of Renal Replacement Therapy in Critically Ill Patients: Systematic Review and Meta-Analysis. Journal of intensive Care Medicine 34; 714-722.

22. Bhatt GC, Das RR, Satapathy A (2021): Early versus Late Initiation of Renal Replacement Therapy: Have We Reached the Consensus? An Updated Meta-Analysis. 145; 371-385.

23. Bolgiaghi L, Umbrello M, Formenti P, Coppola S, Sabbatini G, Massaro C, Damiani M, Chiumello D (2021): The furosemide stress test, electrolyte response and Renal Index in critically ill patients. Minerva Anestesiol 87; 448-457.

24. Bonnassieux M, Duclos A, Schneider AG, Schmidt A, Bénard S, Cancalon C, Joannes-Boyau O, Ichai C, Constantin JM, Lefrant JY et al. (2018): Renal Replacement Therapy modality in the ICU and renal recovery at Hospital Discharge. Crit Care Med 46; e102-e110.

25. Borthwick EM, Hill CJ, Rabindranath KS, Maxwell AP, Mcauley DF, Blackwood B (2013): High-volume haemofiltration for sepsis. Cochrane Database Syst Rev; Cd008075.

26. Borthwick EM, Hill CJ, Rabindranath KS, Maxwell AP, Mcauley DF, Blackwood B (2017): High-volume haemofiltration for sepsis in adults. Cochrane Database Syst Rev 1; Cd008075.

27. Bouchard J, Soroko SB, Chertow GM, Himmelfarb J, Ikizler TA, Paganini EP, Mehta RL (2009): Fluid accumulation, survival and recovery of kidney function in critically ill patients with acute kidney injury. Kidney Int 76; 422-427.

28. Bouman CS, Oudemans-Van Straaten HM, Tijssen JG, Zandstra DF, Kesecioglu J (2002): Effects of early high-volume continuous venovenous hemofiltration on survival and recovery of renal function in intensive care patients with acute renal failure: a prospective, randomized trial. Critical care medicine 30; 2205‐2211.

29. Boussekey N, Chiche A, Faure K, Devos P, Guery B, D'escrivan T, Georges H, Leroy O (2008): A pilot randomized study comparing high and low volume hemofiltration on vasopressor use in septic shock. intensive care medicine 34; 1646‐1653.

30. Cantarovich F, Rangoonwala B, Lorenz H, Verho M, Esnault VL (2004): High-dose furosemide for established ARF: a prospective, randomized, double-blind, placebo-controlled, multicenter trial. Am J Kidney Dis 44; 402-409.

31. Cardoso FS, Gottfried M, Tujios S, Olson JC, Karvellas CJ (2018): continuous renal replacement therapy is associated with reduced serum ammonia levels and mortality in acute liver failure. Hepatology 67; 711-720.

32. Casey ET, Gupta BP, Erwin PJ, Montori VM, Murad MH (2010): The dose of continuous renal replacement therapy for acute renal failure: A systematic review and meta-analysis. Renal Failure 32; 555-561.

33. Chang H, Gong Y, Li C, Ma Z (2021): Clinical efficacy of regional citrate anticoagulation in continuous renal replacement therapy: systematic review and meta-analysis. Ann Palliat Med 10; 8939-8951.

34. Chaudhuri D, Herritt B, Heyland D, Gagnon LP, Thavorn K, Kobewka D, Kyeremanteng K (2019): Early Renal Replacement Therapy Versus Standard Care in the ICU: A Systematic Review, Meta-Analysis, and Cost Analysis. Journal of intensive Care Medicine 34; 323-329.

35. Chawla LS, Davison DL, Brasha-Mitchell E, Koyner JL, Arthur JM, Shaw AD, Tumlin JA, Trevino SA, Kimmel PL, Seneff MG (2013): Development and standardization of a furosemide stress test to predict the severity of acute kidney injury. Crit Care 17; R207.

36. Chen JJ, Chang CH, Huang YT, Kuo G (2020): Furosemide stress test as a predictive marker of acute kidney injury progression or renal replacement therapy: a systemic review and meta-analysis. Crit Care 24; 202.

37. Chen P, Chen F, Lei J, Zhou B (2020): Clinical outcomes of continuous vs intermittent meropenem infusion for the treatment of sepsis: A systematic review and meta-analysis. 29; 993-1000.

38. Chen X, Chen Z, Wei T, Li P, Zhang L, Fu P (2019): The Effect of Serum Neutrophil Gelatinase-Associated Lipocalin on the Discontinuation of continuous Renal Replacement Therapy in Critically Ill Patients with Acute Kidney Injury. Blood Purif 48; 10-17.

39. Choi YH, Lee DH, Oh JH, Wee JH, Jang TC, Choi SP, Park KN, Korean Hypothermia Network I (2020): Renal replacement therapy is independently associated with a lower risk of death in patients with severe acute kidney injury treated with targeted temperature management after out-of-hospital cardiac arrest. Crit Care 24; 115.

40. Clark E, Molnar AO, Joannes-Boyau O, Honore PM, Sikora L, Bagshaw SM (2014): High-volume hemofiltration for septic acute kidney injury: a systematic review and meta-analysis. Crit Care 18; R7.

41. Combes A, Bréchot N, Amour J, Cozic N, Lebreton G, Guidon C, Zogheib E, Thiranos JC, Rigal JC, Bastien O et al. (2015): Early High-Volume Hemofiltration versus Standard Care for Post-Cardiac Surgery Shock. The HEROICS Study. Am J Respir Crit Care Med 192; 1179-1190.

42. Conger JD (1975): A controlled evaluation of prophylactic dialysis in post-traumatic acute renal failure. J Trauma 15; 1056-1063.

43. Connor MJ, Jr., Salem C, Bauer SR, Hofmann CL, Groszek J, Butler R, Rehm SJ, Fissell WH (2011): Therapeutic drug monitoring of piperacillin-tazobactam using spent dialysate effluent in patients receiving continuous venovenous hemodialysis. Antimicrob Agents Chemother 55; 557-560.

44. Côté JM, Pinard L, Cailhier JF, Lévesque R, Murray PT, Beaubien-Souligny W (2022): Intermittent Convective Therapies in Patients with Acute Kidney Injury: A Systematic Review with Meta-Analysis. Blood Purif 51; 75-86.

45. Daniels JR, Ma JZ, Cao Z, Beger RD, Sun J, Schnackenberg L, Pence L, Choudhury D, Palevsky PM, Portilla D et al. (2021): Discovery of Novel Proteomic Biomarkers for the Prediction of Kidney Recovery from Dialysis-Dependent AKI Patients. Kidney360 2; 1716-1727.

46. Davenport A, Will EJ, Davison AM (1993): Effect of renal replacement therapy on patients with combined acute renal and fulminant hepatic failure. Kidney Int Suppl 41; S245-251.

47. Davenport A, Will EJ, Davison AM, Swindells S, Cohen AT, Miloszewski KJ, Losowsky MS (1989): Changes in intracranial pressure during haemofiltration in oliguric patients with grade IV hepatic encephalopathy. Nephron 53; 142-146.

48. Decker BS, Goldfarb DS, Dargan PI, Friesen M, Gosselin S, Hoffman RS, Lavergne V, Nolin TD, Ghannoum M (2015): Extracorporeal Treatment for Lithium Poisoning: Systematic Review and Recommendations from the EXTRIP Workgroup. Clin J Am Soc Nephrol 10; 875-887.

49. Dewitte A, Joannès-Boyau O, Sidobre C, Fleureau C, Bats ML, Derache P, Leuillet S, Ripoche J, Combe C, Ouattara A (2015): Kinetic eGFR and Novel AKI Biomarkers to Predict renal recovery. Clin J Am Soc Nephrol 10; 1900-1910.

50. Dhaese S, Heffernan A, Liu D, Abdul-Aziz MH, Stove V, Tam VH, Lipman J, Roberts JA, De Waele JJ (2020): Prolonged Versus Intermittent Infusion of β-Lactam Antibiotics: A Systematic Review and Meta-Regression of Bacterial Killing in Preclinical Infection Models. 59; 1237-1250.

51. Duceppe MA, Kanji S, Do AT, Ruo N, Cavayas YA, Albert M, Robert-Halabi M, Zavalkoff S, Dupont P, Samoukovic G et al. (2021): Pharmacokinetics of Commonly Used Antimicrobials in Critically Ill Adults During Extracorporeal Membrane Oxygenation: A Systematic Review. 81; 1307-1329.

52. Dulhunty JM, Brett SJ, De Waele JJ, Rajbhandari D, Billot L, Cotta MO, Davis JS, Finfer S, Hammond NE, Knowles S et al. (2024): continuous vs Intermittent beta-Lactam Antibiotic Infusions in Critically Ill Patients With Sepsis: The BLING III Randomized Clinical Trial. JAMA 332; 629-637.

53. Dulhunty JM, Roberts JA, Davis JS, Webb SA, Bellomo R, Gomersall C, Shirwadkar C, Eastwood GM, Myburgh J, Paterson DL et al. (2013): continuous infusion of beta-lactam antibiotics in severe sepsis: a multicenter double-blind, randomized controlled trial. Clin Infect Dis 56; 236-244.

54. Dulhunty JM, Roberts JA, Davis JS, Webb SA, Bellomo R, Gomersall C, Shirwadkar C, Eastwood GM, Myburgh J, Paterson DL et al. (2015): A Multicenter Randomized Trial of continuous versus Intermittent β-Lactam Infusion in Severe Sepsis. Am J Respir Crit Care Med 192; 1298-1305.

55. Economou CJP, Wong G, Mcwhinney B, Ungerer JPJ, Lipman J, Roberts JA (2017): Impact of β-lactam antibiotic therapeutic drug monitoring on dose adjustments in critically ill patients undergoing continuous renal replacement therapy. Int J Antimicrob Agents 49; 589-594.

56. El-Haffaf I, Caissy JA, Marsot A (2021): Piperacillin-Tazobactam in intensive Care Units: A Review of Population Pharmacokinetic Analyses. 60; 855-875.

57. Eyer F, Pfab R, Felgenhauer N, Lutz J, Heemann U, Steimer W, Zondler S, Fichtl B, Zilker T (2006): Lithium poisoning: pharmacokinetics and clearance during different therapeutic measures. J Clin Psychopharmacol 26; 325-330.

58. Falagas ME, Tansarli GS, Ikawa K, Vardakas KZ (2013): Clinical outcomes with extended or continuous versus short-term intravenous infusion of carbapenems and piperacillin/tazobactam: A systematic review and meta-analysis. 56; 272-282.

59. Faulhaber-Walter R, Hafer C, Jahr N, Vahlbruch J, Hoy L, Haller H, Fliser D, Kielstein JT (2009): The Hannover Dialysis Outcome study: comparison of standard versus intensified extended dialysis for treatment of patients with acute kidney injury in the intensive care unit. Nephrology, dialysis, transplantation 24; 2179‐2186.

60. Fawaz S, Barton S, Nabhani-Gebara S (2020): Comparing clinical outcomes of piperacillin-tazobactam administration and dosage strategies in critically ill adult patients: a systematic review and meta-analysis. BMC Infect Dis 20; 430.

61. Fayad AI, Buamscha DG, Ciapponi A (2016): Intensity of continuous renal replacement therapy for acute kidney injury. Cochrane Database of Systematic Reviews; na..

62. Fayad AI, Buamscha DG, Ciapponi A (2022): Timing of kidney replacement therapy initiation for acute kidney injury. Cochrane Database Syst Rev 11; CD010612.

63. Fayad AII, Buamscha DG, Ciapponi A (2018): Timing of renal replacement therapy initiation for acute kidney injury. Cochrane Database Syst Rev 12; CD010612.

64. Feng YM, Yang Y, Han XL, Zhang F, Wan D, Guo R (2017): The effect of early versus late initiation of renal replacement therapy in patients with acute kidney injury: A meta-analysis with trial sequential analysis of randomized controlled trials. PLoS One 12; e0174158.

65. Fisher C, Baldwin I, Fealy N, Naorungroj T, Bellomo R (2022): Ammonia Clearance with Different continuous Renal Replacement Therapy Techniques in Patients with Liver Failure. Blood Purif 51; 840-846.

66. Flannery AH, Bissell BD, Bastin MT, Morris PE, Neyra JA (2020): continuous Versus Intermittent Infusion of Vancomycin and the Risk of Acute Kidney Injury in Critically Ill Adults: A Systematic Review and Meta-Analysis. 48; 912-918.

67. Fletcher JJ, Bergman K, Carlson G, Feucht EC, Blostein PA (2010): continuous renal replacement therapy for refractory intracranial hypertension? J Trauma 68; 1506-1509.

68. Fox HM, Decleene JH (2023): Relationship Between Mean Arterial Pressure and Furosemide Stress Test Success Rates: A Retrospective Cohort Study. Ann Pharmacother 57; 44-50.

69. Frohlich S, Donnelly A, Solymos O, Conlon N (2012): Use of 2-hour creatinine clearance to guide cessation of continuous renal replacement therapy. J Crit Care 27; 744 e741-745.

70. Gaião SM, Gomes AA, Paiva JA (2016): Prognostics factors for mortality and renal recovery in critically ill patients with acute kidney injury and renal replacement therapy. Rev Bras Ter Intensiva 28; 70-77.

71. Garcés EO, Victorino JA, Thomé FS, Röhsig LM, Dornelles E, Louzada M, Stifft J, De Holanda F, Veronese FV (2010): Enoxaparin versus unfractioned Heparin as anticoagulant for continuous venovenous hemodialysis: a randomized open-label trial. Renal failure 32; 320‐327.

72. Gaudry S, Grolleau F, Barbar S, Martin-Lefevre L, Pons B, Boulet E, Boyer A, Chevrel G, Montini F, Bohe J et al. (2022): continuous renal replacement therapy versus intermittent hemodialysis as first modality for renal replacement therapy in severe acute kidney injury: a secondary analysis of AKIKI and IDEAL-ICU studies. Crit Care 26; 93.

73. Gaudry S, Hajage D, Benichou N, Chaïbi K, Barbar S, Zarbock A, Lumlertgul N, Wald R, Bagshaw SM, Srisawat N et al. (2020): Delayed versus early initiation of renal replacement therapy for severe acute kidney injury: a systematic review and individual patient data meta-analysis of randomised clinical trials. Lancet (London, England) 395; 1506-1515.

74. Gaudry S, Hajage D, Martin-Lefevre L, Lebbah S, Louis G, Moschietto S, Titeca-Beauport D, Combe B, Pons B, De Prost N et al. (2021): Comparison of two delayed strategies for renal replacement therapy initiation for severe acute kidney injury (AKIKI 2): a multicentre, open-label, randomised, controlled trial. Lancet 397; 1293-1300.

75. Gaudry S, Hajage D, Schortgen F, Martin-Lefevre L, Pons B, Boulet E, Boyer A, Chevrel G, Lerolle N, Carpentier D et al. (2016): Initiation Strategies for Renal-Replacement Therapy in the intensive Care Unit. New England journal of medicine 375; 122‐133.

76. Ghahramani N, Shadrou S, Hollenbeak C (2008): A systematic review of continuous renal replacement therapy and intermittent haemodialysis in management of patients with acute renal failure. Nephrology (Carlton) 13; 570-578.

77. Ghani RA, Zainudin S, Ctkong N, Rahman AF, Wafa SR, Mohamad M, Manaf MR, Ismail R (2006): Serum IL-6 and IL-1-ra with sequential organ failure assessment scores in septic patients receiving high-volume haemofiltration and continuous venovenous haemofiltration. Nephrology (Carlton) 11; 386-393.

78. Gillum DM, Dixon BS, Yanover MJ, Kelleher SP, Shapiro MD, Benedetti RG, Dillingham MA, Paller MS, Goldberg JP, Tomford RC et al. (1986): The role of intensive dialysis in acute renal failure. Clin Nephrol 25; 249-255.

79. Hagel S, Bach F, Brenner T, Bracht H, Brinkmann A, Annecke T, Hohn A, Weigand M, Michels G, Kluge S et al. (2022): Effect of therapeutic drug monitoring-based dose optimization of piperacillin/tazobactam on sepsis-related organ dysfunction in patients with sepsis: a randomized controlled trial. intensive care medicine 48; 311‐321.

80. Han SS, Bae E, Song SH, Kim DK, Kim YS, Han JS, Joo KW, Bagshaw SM, Bellomo R, Devarajan P et al. (2016): NT-proBNP Is Predictive of the Weaning from continuous Renal Replacement Therapy. Tohoku J Exp Med 239; 1-8.

81. Hao JJ, Chen H, Zhou JX (2016): continuous versus intermittent infusion of vancomycin in adult patients: A systematic review and meta-analysis. Int J Antimicrob Agents 47; 28-35.

82. Heidari S, Khalili H (2023): Linezolid pharmacokinetics: a systematic review for the best clinical practice. Eur J Clin Pharmacol 79; 195-206.

83. Heise D, Gries D, Moerer O, Bleckmann A, Quintel M (2012): Predicting restoration of kidney function during CRRT-free intervals. J Cardiothorac Surg 7; 6.

84. Hoff BM, Maker JH, Dager WE, Heintz BH (2020): Antibiotic Dosing for Critically Ill Adult Patients Receiving Intermittent Hemodialysis, Prolonged Intermittent Renal Replacement Therapy, and continuous Renal Replacement Therapy: An Update. Annals of Pharmacotherapy 54; 43-55.

85. Huang H, Zhou Q, Chen MH (2021): High-volume hemofiltration reduces short-term mortality with no influence on the incidence of MODS, hospital stay, and hospitalization cost in patients with severe-acute pancreatitis: A meta-analysis. Artif Organs 45; 1456-1465.

86. Hui LA, Bodolea C, Vlase L, Hiriscau EI, Popa A (2022): Linezolid Administration to Critically Ill Patients: Intermittent or continuous Infusion? A Systematic Literature Search and Review. 11; 1-15.

87. Ishikawa K, Shibutani K, Kawai F, Ota E, Takahashi O, Mori N (2023): Effectiveness of Extended or continuous vs. Bolus Infusion of Broad-Spectrum Beta-Lactam Antibiotics for Febrile Neutropenia: A Systematic Review and Meta-Analysis. 12; 1024-1030.

88. Itenov TS, Berthelsen RE, Jensen JU, Gerds TA, Pedersen LM, Strange D, Thormar K, Loken J, Andersen MH, Tousi H et al. (2018): Predicting recovery from acute kidney injury in critically ill patients: development and validation of a prediction model. Crit Care Resusc 20; 54-60.

89. Jacobs R, Verbrugghe W, Dams K, Roelant E, Couttenye MM, Devroey D, Jorens P (2023): Regional citrate Anticoagulation in continuous Renal Replacement Therapy: Is Metabolic Fear the Enemy of Logic? A Systematic Review and Meta-Analysis of Randomised Controlled trials. Life (Basel) 13; 1198.

90. Jamal JA, Mat-Nor MB, Mohamad-Nor FS, Udy AA, Wallis SC, Lipman J, Roberts JA (2015): Pharmacokinetics of meropenem in critically ill patients receiving continuous venovenous haemofiltration: a randomised controlled trial of continuous infusion versus intermittent bolus administration. Int J Antimicrob Agents 45; 41-45.

91. Jamal JA, Roberts DM, Udy AA, Mat-Nor MB, Mohamad-Nor FS, Wallis SC, Lipman J, Roberts JA (2015): Pharmacokinetics of piperacillin in critically ill patients receiving continuous venovenous haemofiltration: A randomised controlled trial of continuous infusion versus intermittent bolus administration. Int J Antimicrob Agents 46; 39-44.

92. Jamal JA, Udy AA, Lipman J, Roberts JA (2014): The impact of variation in renal replacement therapy settings on piperacillin, meropenem, and vancomycin drug clearance in the critically ill: an analysis of published literature and dosing regimens*. Crit Care Med 42; 1640-1650.

93. Jang M (2021): Survival comparison between continuous venovenous hemodiafiltration (CVVHDF) and continuous venovenous hemofiltration (CVVH) for septic AKI. Journal of the American Society of Nephrology : JASN 32; 131.

94. Jang M, Lee SW (2022): SURVIVAL COMPARISON BETWEEN CVVHDF AND CVVH FOR SEPTIC ACUTE KIDNEY INJURY. Nephrology dialysis transplantation 37; i462.

95. Jeon J, Kim DH, Baeg SI, Lee EJ, Chung CR, Jeon K, Lee JE, Huh W, Suh GY, Kim YG et al. (2018): Association between diuretics and successful discontinuation of continuous renal replacement therapy in critically ill patients with acute kidney injury. Crit Care 22; 255.

96. Joannes-Boyau O, Honoré PM, Perez P, Bagshaw SM, Grand H, Canivet JL, Dewitte A, Flamens C, Pujol W, Grandoulier AS et al. (2013): High-volume versus standard-volume haemofiltration for septic shock patients with acute kidney injury (IVOIRE study): a multicentre randomized controlled trial. intensive Care Med 39; 1535-1546.

97. Joannidis M, Kountchev J, Rauchenzauner M, Schusterschitz N, Ulmer H, Mayr A, Bellmann R (2007): Enoxaparin vs. unfractionated Heparin for anticoagulation during continuous veno-venous hemofiltration: a randomized controlled crossover study. intensive care medicine 33; 1571‐1579.

98. Johansen N, Kjaergaard KD, Peters CD, Pedersen M, Jespersen B, Jensen JD (2017): Brain swelling during dialysis: A randomized trial comparing low-flux hemodialysis with pre-dilution hemodiafiltration Clin Nephrol 87 (2017); 221-230.

99. John S, Griesbach D, Baumgartel M, Weihprecht H, Schmieder RE, Geiger H (2001): Effects of continuous haemofiltration vs intermittent haemodialysis on systemic haemodynamics and splanchnic regional perfusion in septic shock patients: a prospective, randomized clinical trial. Nephrol Dial Transplant 16; 320-327.

100. Jun M, Heerspink HJ, Ninomiya T, Gallagher M, Bellomo R, Myburgh J, Finfer S, Palevsky PM, Kellum JA, Perkovic V et al. (2010): Intensities of renal replacement therapy in acute kidney injury: a systematic review and meta-analysis. Clin J Am Soc Nephrol 5; 956-963.

101. Junhai Z, Beibei C, Jing Y, Li L (2019): Effect of High-Volume Hemofiltration in Critically Ill Patients: A Systematic Review and Meta-Analysis. Med Sci Monit 25; 3964-3975.

102. Karvellas C, Farhat M, Sajjad I, Mogensen S, Bagshaw S (2010): Timing of initiation of renal replacement therapy in acute kidney injury: a meta-analysis. Critical care medicine 38; A105‐.

103. Karvellas CJ, Farhat MR, Sajjad I, Mogensen SS, Leung AA, Wald R, Bagshaw SM (2011): A comparison of early versus late initiation of renal replacement therapy in critically ill patients with acute kidney injury: A systematic review and meta-analysis. Critical Care 15; R72.

104. Kawarazaki H, Uchino S, Tokuhira N, Ohnuma T, Namba Y, Katayama S, Toki N, Takeda K, Yasuda H, Izawa J et al. (2013): Who may not benefit from continuous renal replacement therapy in acute kidney injury? Hemodial Int 17; 624-632.

105. Kielstein JT, Kretschmer U, Ernst T, Hafer C, Bahr MJ, Haller H, Fliser D (2004): Efficacy and cardiovascular tolerability of extended dialysis in critically ill patients: a randomized controlled study. Am J Kidney Dis 43; 342-349.

106. Kim CS, Bae EH, Ma SK, Kim SW, Pajewski R, Gipson P, Heung M, Tourneur JM, Weissbrich C, Putensen C et al. (2018): A Prospective Observational Study on the Predictive Value of Serum Cystatin C for Successful Weaning from continuous Renal Replacement Therapy. Kidney Blood Press Res 43; 872-881.

107. King JD, Kern MH, Jaar BG (2019): Extracorporeal Removal of Poisons and Toxins. Clin J Am Soc Nephrol 14; 1408-1415.

108. Kirsch AH, Lyko R, Nilsson LG, Beck W, Amdahl M, Lechner P, Schneider A, Wanner C, Rosenkranz AR, Krieter DH (2017): Performance of hemodialysis with novel medium cut-off dialyzers. Nephrol Dial Transplant 32; 165-172.

109. Klingele M, Bomberg H, Lerner-Gräber A, Fliser D, Poppleton A, Schäfers HJ, Groesdonk HV (2014): Use of Argatroban: experiences in continuous renal replacement therapy in critically ill patients after cardiac surgery. J Thorac Cardiovasc Surg 147; 1918-1924.

110. Kovacs B, Sullivan KJ, Hiremath S, Patel RV (2017): Effect of sustained low efficient dialysis versus continuous renal replacement therapy on renal recovery after acute kidney injury in the intensive care unit: A systematic review and meta-analysis. Nephrology 22; 343-353.

111. Koyner JL, Davison DL, Brasha-Mitchell E, Chalikonda DM, Arthur JM, Shaw AD, Tumlin JA, Trevino SA, Bennett MR, Kimmel PL et al. (2015): Furosemide Stress Test and Biomarkers for the Prediction of AKI Severity. J Am Soc Nephrol 26; 2023-2031.

112. Koyner JL, Mackey RH, Echeverri J, Rosenthal NA, Carabuena LA, Bronson-Lowe D, Harenski K, Neyra JA (2024): Initial renal replacement therapy (RRT) modality associates with 90-day postdischarge RRT dependence in critically ill AKI survivors. J Crit Care 82; 154764.

113. Langgartner J, Vasold A, Glück T, Reng M, Kees F (2008): Pharmacokinetics of meropenem during intermittent and continuous intravenous application in patients treated by continuous renal replacement therapy. intensive care medicine 34; 1091‐1096.

114. Lavonas EJ, Buchanan, J. (2015): Hemodialysis for lithium poisoning. Cochrane Database Syst Rev 2015; Cd007951.

115. Lee YR, Miller PD, Alzghari SK, Blanco DD, Hager JD, Kuntz KS (2018): continuous Infusion Versus Intermittent Bolus of Beta-Lactams in Critically Ill Patients with Respiratory Infections: A Systematic Review and Meta-analysis. 43; 155-170.

116. Li P, Qu LP, Qi D, Shen B, Wang YM, Xu JR, Jiang WH, Zhang H, Ding XQ, Teng J (2017): High-dose versus low-dose haemofiltration for the treatment of critically ill patients with acute kidney injury: An updated systematic review and meta-analysis. BMJ Open 7; e014171.

117. Li R, Gao X, Zhou T, Li Y, Wang J, Zhang P (2022): Regional citrate versus Heparin anticoagulation for continuous renal replacement therapy in critically ill patients: A meta-analysis of randomized controlled trials. Ther Apher Dial 26; 1086-1097.

118. Li X, Liu C, Mao Z, Li Q, Zhou F (2021): Timing of renal replacement therapy initiation for acute kidney injury in critically ill patients: a systematic review of randomized clinical trials with meta-analysis and trial sequential analysis. Crit Care 25; 1-15.

119. Li Y, Li H, Zhang D (2019): Timing of continuous renal replacement therapy in patients with septic AKI: A systematic review and meta-analysis. Medicine (Baltimore) 98; e16800.

120. Li Y, Zhang Y, Li R, Zhang M, Gao X (2022): Timing of initiation of renal replacement therapy for patients with acute kidney injury: A meta-analysis of RCTs. Ther Apher Dial;

121. Liang KV, Sileanu FE, Clermont G, Murugan R, Pike F, Palevsky PM, Kellum JA (2016): modality of RRT and Recovery of Kidney Function after AKI in Patients Surviving to Hospital Discharge. Clin J Am Soc Nephrol 11; 30-38.

122. Liao YJ, Zhang L, Zeng XX, Fu P (2013): citrate versus unfractionated Heparin for anticoagulation in continuous renal replacement therapy. Chin Med J (Engl) 126; 1344-1349.

123. Link A, Girndt M, Selejan S, Mathes A, Böhm M, Rensing H (2009): Argatroban for anticoagulation in continuous renal replacement therapy. Crit Care Med 37; 105-110.

124. Liu C, Mao Z, Kang H, Hu J, Zhou F (2016): Regional citrate versus Heparin anticoagulation for continuous renal replacement therapy in critically ill patients: a meta-analysis with trial sequential analysis of randomized controlled trials. Crit Care 20; 144.

125. Liu Y, Davari-Farid S, Arora P, Porhomayon J, Nader ND (2014): Early versus late initiation of renal replacement therapy in critically ill patients with acute kidney injury after cardiac surgery: A systematic review and meta-analysis. Journal of Cardiothoracic and Vascular Anesthesia 28; 557-563.

126. Liu Y, Ge XH, Guo HL, Chen F, Zhang Y, Xu J, Ji X, Miao HJ (2023): A Systematic Review of Linezolid Pharmacokinetics/Pharmacodynamics in Patients Undergoing continuous Renal Replacement Therapy: Does One Size Fit All? Curr Drug Metab 24; 70-77.

127. Lopez JC, Perez X, Labad J, Esteve F, Manez R, Javierre C (2012): Higher requirements of dialysis in severe lithium intoxication. Hemodial Int 16; 407-413.

128. Lumlertgul N, Peerapornratana S, Trakarnvanich T, Pongsittisak W, Surasit K, Chuasuwan A, Tankee P, Tiranathanagul K, Praditpornsilpa K, Tungsanga K et al. (2018): Early versus standard initiation of renal replacement therapy in furosemide stress test non-responsive acute kidney injury patients (the FST trial). Crit Care 22; 101.

129. Luo J, Liao J, Cai R, Liu J, Huang Z, Cheng Y, Yang Z, Liu Z (2019): Prolonged versus Intermittent Infusion of Antibiotics in Acute and Severe Infections: A Meta-analysis. Arch Iran Med 22; 612-626.

130. Luxton T, King N, Walti C, Jeuken L, Sandoe J (2022): A systematic review of the effect of therapeutic drug monitoring on patient health outcomes during treatment with Penicillin s. J Antimicrob Chemother 77; 1532-1541.

131. Mann L, Ten Eyck P, Wu C, Story M, Jenigiri S, Patel J, Honkanen I, O'connor K, Tener J, Sambharia M et al. (2023): CVVHD results in longer filter life than pre-filter CVVH: Results of a quasi-randomized clinical trial. PLoS One 18; e0278550.

132. Matsuura R, Komaru Y, Miyamoto Y, Yoshida T, Yoshimoto K, Isshiki R, Mayumi K, Yamashita T, Hamasaki Y, Nangaku M et al. (2018): Response to different furosemide doses predicts AKI progression in ICU patients with elevated plasma NGAL levels. Ann intensive Care 8; 8.

133. Matusik E, Boidin C, Friggeri A, Richard JC, Bitker L, Roberts JA, Goutelle S (2022b): Therapeutic Drug Monitoring of Antibiotic Drugs in Patients Receiving continuous Renal Replacement Therapy or Intermittent Hemodialysis: A Critical Review. Ther Drug Monit 44; 86-102.

134. Meersch M, Weiss R, Gerss J, Albert F, Gruber J, Kellum JA, Chawla L, Forni LG, Koyner JL, Von Groote T et al. (2023): Predicting the Development of Renal Replacement Therapy Indications by Combining the Furosemide Stress Test and Chemokine (C-C Motif) Ligand 14 in a Cohort of Postsurgical Patients. Crit Care Med 51; 1033-1042.

135. Meertens JH, Jagernath DR, Eleveld DJ, Zijlstra JG, Franssen CF (2009): Haemodialysis followed by continuous veno-venous haemodiafiltration in lithium intoxication; a model and a case. Eur J Intern Med 20; e70-73.

136. Mehta RL, Mcdonald B, Gabbai FB, Pahl M, Pascual MT, Farkas A, Kaplan RM, Collaborative Group for Treatment Of ARFITICU (2001): A randomized clinical trial of continuous versus intermittent dialysis for acute renal failure. Kidney Int 60; 1154-1163.

137. Mendu ML, Ciociolo GR, Jr., Mclaughlin SR, Graham DA, Ghazinouri R, Parmar S, Grossier A, Rosen R, Laskowski KR, Riella LV et al. (2017): A Decision-Making Algorithm for Initiation and Discontinuation of RRT in Severe AKI. Clin J Am Soc Nephrol 12; 228-236.

138. Monti G, Bradic N, Marzaroli M, Konkayev A, Fominskiy E, Kotani Y, Likhvantsev VV, Momesso E, Nogtev P, Lobreglio R et al. (2023): continuous vs Intermittent Meropenem Administration in Critically Ill Patients With Sepsis: the MERCY Randomized Clinical Trial. JAMA 330; 141-151.

139. Nadkarni GN, Patel AA, Konstantinidis I, Mahajan A, Agarwal SK, Kamat S, Annapureddy N, Benjo A, Thakar CV (2015): Dialysis Requiring Acute Kidney Injury in Acute Cerebrovascular Accident Hospitalizations. Stroke 46; 3226-3231.

140. Naka T, Bellomo R, Morimatsu H, Rocktaschel J, Wan L, Gow P, Angus P (2006): Acid-base balance during continuous veno-venous hemofiltration: the impact of severe hepatic failure. Int J Artif Organs 29; 668-674.

141. Naorungroj T, Neto AS, Wang A, Gallagher M, Bellomo R (2022): Renal outcomes according to renal replacement therapy modality and treatment protocol in the ATN and RENAL trials. Crit Care 26; 269.

142. Naorungroj T, Neto AS, Yanase F, Eastwood G, Wald R, Bagshaw SM, Bellomo R (2021): Time to Initiation of Renal Replacement Therapy Among Critically Ill Patients With Acute Kidney Injury: A Current Systematic Review and Meta-Analysis. 49; E781-E792.

143. Nash DM, Przech S, Wald R, O'reilly D (2017): Systematic review and meta-analysis of renal replacement therapy modalities for acute kidney injury in the intensive care unit. J Crit Care 41; 138-144.

144. Natale P, Palmer SC, Ruospo M, Longmuir H, Dodds B, Prasad R, Batt TJ, Jose MD, Strippoli GF (2024): Anticoagulation for people receiving long-term haemodialysis. Cochrane Database Syst Rev 1; CD011858.

145. Negash DT, Dhingra VK, Copland M, Griesdale D, Henderson W (2011): Intensity of continuous renal replacement therapy in acute kidney injury in the intensive care unit: A systematic review and meta-analysis. Vascular and Endovascular Surgery 45; 504-510.

146. Osgood M, Compton R, Carandang R, Hall W, Kershaw G, Muehlschlegel S (2015): Rapid unexpected brain herniation in association with renal replacement therapy in acute brain injury: caution in the neurocritical care unit. Neurocrit Care 22; 176-183.

147. Oudemans-Van Straaten HM, Bosman RJ, Koopmans M, Van Der Voort PH, Wester JP, Van Der Spoel JI, Dijksman LM, Zandstra DF (2009): citrate anticoagulation for continuous venovenous hemofiltration. Crit Care Med 37; 545-552.

148. Palevsky PM, O'connor TZ, Chertow GM, Crowley ST, Zhang JH, Kellum JA, Network USDOVaNIOHaRFT (2009): Intensity of renal replacement therapy in acute kidney injury: perspective from within the Acute Renal Failure Trial Network Study. Crit Care 13; 310.

149. Pan HC, Chen YY, Tsai IJ, Shiao CC, Huang TM, Chan CK, Liao HW, Lai TS, Chueh Y, Wu VC et al. (2021): Accelerated versus standard initiation of renal replacement therapy for critically ill patients with acute kidney injury: a systematic review and meta-analysis of RCT studies. 25; 5.

150. Pan HC, Huang TTM, Huang CT, Sun CY, Chen YM, Wu VC (2022): Urinary Biomarkers Can Predict Weaning From Acute Dialysis Therapy in Critically Ill Patients. Archives of pathology & laboratory medicine 146; 1353-1363.

151. Pan HC, Sun CY, Huang TTM, Huang CT, Tsao CH, Lai CH, Chen YM, Wu VC (2022a): Distinct Subtyping of Successful Weaning from Acute Kidney Injury Requiring Renal Replacement Therapy by Consensus Clustering in Critically Ill Patients. Biomedicines 10; 1-16.

152. Pannu N, Klarenbach S, Wiebe N, Manns B, Tonelli M, Alberta Kidney Disease N (2008): Renal replacement therapy in patients with acute renal failure: a systematic review. JAMA 299; 793-805.

153. Park JT, Lee H, Kee YK, Park S, Oh HJ, Han SH, Joo KW, Lim CS, Kim YS, Kang SW et al. (2016): High-Dose Versus Conventional-Dose continuous Venovenous Hemodiafiltration and Patient and Kidney Survival and Cytokine Removal in Sepsis-Associated Acute Kidney Injury: A Randomized Controlled Trial. Am J Kidney Dis 68; 599-608.

154. Pasin L, Boraso S, Tiberio I (2019): Early initiation of renal replacement therapy in critically ill patients: A meta-analysis of randomized clinical trials. BMC Anaesth 19; 62-70.

155. Peces R, Pobes A (2001): Effectiveness of haemodialysis with high-flux membranes in the extracorporeal therapy of life-threatening acute lithium intoxication. Nephrol Dial Transplant 16; 1301-1303.

156. Peltonen S, Ahlstrom A, Kylavainio V, Honkanen E, Pettila V (2007): The effect of combining intermittent hemodiafiltration with forced alkaline diuresis on plasma Myoglobin in rhabdomyolysis. Acta Anaesthesiol Scand 51; 553-558.

157. Peng B, Lu J, Guo H, Liu J, Li A (2023): Regional citrate anticoagulation for replacement therapy in patients with liver failure: A systematic review and meta-analysis. Front Nutr 10; 1031796.

158. Perrott J, Mabasa VH, Ensom MH (2010): Comparing outcomes of meropenem administration strategies based on pharmacokinetic and pharmacodynamic principles: a qualitative systematic review. Ann Pharmacother 44; 557-564.

159. Philpott CD, Droege CA, Droege ME, Healy DP, Courter JD, Ernst NE, Harger NJ, Foertsch MJ, Winter JB, Carter KE et al. (2019): Pharmacokinetics and Pharmacodynamics of Extended-Infusion Cefepime in Critically Ill Patients Receiving continuous Renal Replacement Therapy: A Prospective, Open-Label Study. Pharmacotherapy 39; 1066-1076.

160. Pon AG, Vairakkani R, Mervin EF, Srinivasaprasad ND, Kaliaperumal T (2021): Clinical significance of frusemide stress test in predicting the severity of acute kidney injury. J Bras Nefrol 43; 470-477.

161. Potier J, Le Roy F, Faucon JP, Besselievre T, Renaudineau E, Farquet C, Soihan P, Touzard D, Djema A, Ilinca T (2013): Elevated removal of middle molecules without significant albumin loss with mixed-dilution hemodiafiltration for patients unable to provide sufficient blood flow rates. Blood Purif 36; 78-83.

162. Premru V, Kovac J, Buturovic-Ponikvar J, Ponikvar R (2013): Some kinetic considerations in high cut-off hemodiafiltration for acute Myoglobin uric renal failure. Ther Apher Dial 17; 396-401.

163. Pschowski R, Briegel S, Von Haehling S, Doehner W, Bender TO, Pape UF, Hasper D, Jörress A, Schefold JC (2015): Effects of dialysis modality on blood loss, bleeding complications and transfusion requirements in critically ill patients with dialysis-dependent acute renal failure. Anaesthesia and intensive care 43; 764‐770.

164. Qi W, Liu J, Li A (2023): Regional citrate Anticoagulation or Heparin Anticoagulation for Renal Replacement Therapy in Patients With Liver Failure: A Systematic Review and Meta-Analysis. Clin Appl Thromb Hemost 29; 10760296231174001.

165. Rabindranath K, Adams J, Macleod AM, Muirhead N (2007): Intermittent versus continuous renal replacement therapy for acute renal failure in adults. Cochrane Database Syst Rev; CD003773.

166. Raina R, Agrawal N, Kusumi K, Pandey A, Tibrewal A, Botsch A (2022): A Meta-Analysis of Extracorporeal Anticoagulants in Pediatric continuous Kidney Replacement Therapy. J intensive Care Med 37; 577-594.

167. Raurich JM, Llompart-Pou JA, Novo MA, Talavera C, Ferreruela M, Ayestaran I (2018): Successful weaning from continuous renal replacement therapy. Associated risk factors. J Crit Care 45; 144-148.

168. Reddy BV, Grossman EJ, Trevino SA, Hursting MJ, Murray PT (2005): Argatroban anticoagulation in patients with Heparin-induced thrombocytopenia requiring renal replacement therapy. Ann Pharmacother 39; 1601-1605.

169. Reeves JH, Cumming AR, Gallagher L, O'brien JL, Santamaria JD (1999): A controlled trial of low-molecular-weight Heparin (dalteparin) versus unfractionated Heparin as anticoagulant during continuous venovenous hemodialysis with filtration. Critical care medicine 27; 2224‐2228.

170. Renal, Investigators RRTS, Bellomo R, Cass A, Cole L, Finfer S, Gallagher M, Lo S, Mcarthur C, Mcguinness S et al. (2009): Intensity of continuous renal-replacement therapy in critically ill patients. N Engl J Med 361; 1627-1638.

171. Rewa OG, Bagshaw SM, Wang X, Wald R, Smith O, Shapiro J, Mcmahon B, Liu KD, Trevino SA, Chawla LS et al. (2019): The furosemide stress test for prediction of worsening acute kidney injury in critically ill patients: A multicenter, prospective, observational study. J Crit Care 52; 109-114.

172. Richter DC, Frey O, Röhr A, Roberts JA, Köberer A, Fuchs T, Papadimas N, Heinzel-Gutenbrunner M, Brenner T, Lichtenstern C et al. (2019): Therapeutic drug monitoring-guided continuous infusion of piperacillin/tazobactam significantly improves pharmacokinetic target attainment in critically ill patients: a retrospective analysis of four years of clinical experience. Infection 47; 1001-1011.

173. Roberts DM, Roberts JA, Roberts MS, Liu X, Nair P, Cole L, Lipman J, Bellomo R (2012): Variability of antibiotic concentrations in critically ill patients receiving continuous renal replacement therapy: a multicentre pharmacokinetic study. Crit Care Med 40; 1523-1528.

174. Roberts JA, Abdul-Aziz MH, Davis JS, Dulhunty JM, Cotta MO, Myburgh J, Bellomo R, Lipman J (2016): continuous versus Intermittent β-Lactam Infusion in Severe Sepsis. A Meta-analysis of Individual Patient Data from Randomized trials. Am J Respir Crit Care Med 194; 681-691.

175. Roberts JA, Croom K, Adomakoh N (2023): continuous infusion of beta-lactam antibiotics: narrative review of systematic reviews, and implications for outpatient parenteral antibiotic therapy. 21; 375-385.

176. Roberts JA, Webb S, Paterson D, Ho KM, Lipman J (2009): A systematic review on clinical benefits of continuous administration of β-lactam antibiotics. 37; 2071-2078.

177. Ronco C, Bellomo R, Brendolan A, Pinna V, La Greca G (1999): Brain density changes during renal replacement in critically ill patients with acute renal failure. continuous hemofiltration versus intermittent hemodialysis. J Nephrol 12; 173-178.

178. Russo DS, Eugenio CS, Balestrin IG, Rodrigues CG, Rosa RG, Teixeira C, Kelly YP, Vieira SRR (2022): Comparison of hemodynamic instability among continuous, intermittent and hybrid renal replacement therapy in acute kidney injury: A systematic review of randomized clinical trials. Journal of Critical Care 69; 153998.

179. Sakhuja A, Bandak G, Barreto EF, Vallabhajosyula S, Jentzer J, Albright R, Kashani KB (2019): Role of Loop Diuretic Challenge in Stage 3 Acute Kidney Injury. Mayo Clin Proc 94; 1509-1515.

180. Sanz-Codina M, Bozkir HO, Jorda A, Zeitlinger M (2023): Individualized antimicrobial dose optimization: a systematic review and meta-analysis of randomized controlled trials. Clin Microbiol Infect 29; 845-857.

181. Saudan P, Niederberger M, De Seigneux S, Romand J, Pugin J, Perneger T, Martin PY (2006): Adding a dialysis dose to continuous hemofiltration increases survival in patients with acute renal failure. Kidney international 70; 1312‐1317.

182. Schefold JC, Von Haehling S, Pschowski R, Bender T, Berkmann C, Briegel S, Hasper D, Jörres A (2014): The effect of continuous versus intermittent renal replacement therapy on the outcome of critically ill patients with acute renal failure (CONVINT): a prospective randomized controlled trial. Crit Care 18; R11.

183. Schiffl H, Lang SM, Fischer R (2002): Daily hemodialysis and the outcome of acute renal failure. N Engl J Med 346; 305-310.

184. Schmidt JJ, Lorenzen J, Chatzikyrkou C, Lichtinghagen R, Kielstein JT (2014): Total collected dialysate lithium concentration after successful dialysis treatment in case of intoxication. BMC Pharmacol Toxicol 15; 49.

185. Schneider AG, Bellomo R, Bagshaw SM, Glassford NJ, Lo S, Jun M, Cass A, Gallagher M (2013): Choice of renal replacement therapy modality and dialysis dependence after acute kidney injury: a systematic review and meta-analysis. intensive Care Med 39; 987-997.

186. Schoenfelder T, Chen X, Bless HH (2017): Effects of continuous and intermittent renal replacement therapies among adult patients with acute kidney injury. GMS Health Technol Assess 13; Doc01.

187. Schwenger V, Weigand MA, Hoffmann O, Dikow R, Kihm LP, Seckinger J, Miftari N, Schaier M, Hofer S, Haar C et al. (2012): Sustained low efficiency dialysis using a single-pass batch system in acute kidney injury - a randomized interventional trial: the REnal Replacement Therapy Study in intensive Care Unit PatiEnts. Crit Care 16; R140.

188. Shawwa K, Kompotiatis P, Sakhuja A, Mccarthy P, Kashani KB (2022): Prolonged exposure to continuous renal replacement therapy in patients with acute kidney injury. J Nephrol 35; 585-595.

189. Shi M, Chen, H, Liu H, Qiu C, Jia R, Ding G (2008): Anticoagulant elect of Argatroban in ESRD patients during induction period of hemodialysis with central venous catheter access. Journal of the American Society of Nephrology 19;

190. Shiu J, Wang E, Tejani AM, Wasdell M (2013): continuous versus intermittent infusions of antibiotics for the treatment of severe acute infections. Cochrane Database Syst Rev 2013; Cd008481.

191. Shotwell MS, Nesbitt R, Madonia PN, Gould ER, Connor MJ, Salem C, Aduroja OA, Amde M, Groszek JJ, Wei P et al. (2016): Pharmacokinetics and pharmacodynamics of extended infusion versus short infusion piperacillin-tazobactam in critically Ill patients undergoing CRRT. Clinical journal of the American Society of Nephrology 11; 1377‐1383.

192. Sime FB, Roberts MS, Peake SL, Lipman J, Roberts JA (2012): Does beta-lactam pharmacokinetic variability in critically III patients justify therapeutic drug monitoring? A systematic review. 2; 2-11.

193. Snow TaC, Littlewood S, Corredor C, Singer M, Arulkumaran N (2021): Effect of Extracorporeal Blood Purification on mortality in Sepsis: A Meta-Analysis and Trial Sequential Analysis. Blood Purification 50; 462-472.

194. Sorrentino SA, Kielstein JT, Lukasz A, Sorrentino JN, Gohrbandt B, Haller H, Schmidt BM (2011): High permeability dialysis membrane allows effective removal of Myoglobin in acute kidney injury resulting from rhabdomyolysis. Crit Care Med 39; 184-186.

195. Spatola L, Maringhini S, Canale C, Granata A, D'amico M (2023): Lithium poisoning and renal replacement therapy: pathophysiology and current clinical recommendations. Int Urol Nephrol; 1-6.

196. Srisawat N, Laoveeravat P, Limphunudom P, Lumlertgul N, Peerapornratana S, Tiranathanagul K, Susantitaphong P, Praditpornsilpa K, Tungsanga K, Eiam-Ong S (2018): The effect of early renal replacement therapy guided by plasma neutrophil gelatinase associated lipocalin on outcome of acute kidney injury: A feasibility study. J Crit Care 43; 36-41.

197. Srisawat N, Murugan R, Lee M, Kong L, Carter M, Angus DC, Kellum JA (2011a): Plasma neutrophil gelatinase-associated lipocalin predicts recovery from acute kidney injury following community-acquired pneumonia. Kidney Int 80; 545-552.

198. Stads S, Kant KM, De Jong MFC, De Ruijter W, Cobbaert CM, Betjes MGH, Gommers D, Oudemans-Van Straaten HM (2019): Predictors of short-term successful discontinuation of continuous renal replacement therapy: results from a prospective multicentre study. BMC Nephrol 20; 129.

199. Stads S, Kant KM, De Jong MFC, De Ruijter W, Cobbaert CM, Betjes MGH, Gommers D, Oudemans-Van Straaten HM (2019a): Predictors of 90-Day Restart of Renal Replacement Therapy after Discontinuation of continuous Renal Replacement Therapy, a Prospective Multicenter Study. Blood Purif 48; 243-252.

200. Steffens NA, Zimmermann ES, Nichelle SM, Brucker N (2021): Meropenem use and therapeutic drug monitoring in clinical practice: a literature review. 46; 610-621.

201. Su Y, Zhang YJ, Tu GW, Hou JY, Ma GG, Hao GW, Xu RH, Luo Z (2024): Furosemide Responsiveness Predicts Acute Kidney Injury Progression After Cardiac Surgery. Ann Thorac Surg 117; 432-438.

202. Sugahara S, Suzuki, H. (2004): Early start on continuous hemodialysis therapy improves survival rate in patients with acute renal failure following coronary bypass surgery. Hemodial Int 8; 320-325.

203. Sun X, Chen Y, Xiao Q, Wang Y, Zhou J, Ma Z, Xiang J, Chen X (2011): Effects of Argatroban as an anticoagulant for intermittent veno-venous hemofiltration (IVVH) in patients at high risk of bleeding. Nephrology, dialysis, transplantation 26; 2954‐2959.

204. Suzuki S, Moriyama K, Hara Y, Hinoue T, Kato Y, Hasegawa D, Kuriyama N, Nakamura T, Komatsu S, Yamashita C et al. (2021): Comparison of Myoglobin clearance in three types of blood purification modalities. Ther Apher Dial 25; 401-406.

205. Thabit AK, Hobbs ALV, Guzman OE, Shea KM (2019): The Pharmacodynamics of Prolonged Infusion β-Lactams for the Treatment of Pseudomonas aeruginosa Infections: A Systematic Review. 41; 2397-2415.e2398.

206. Thomsen J, Sprogøe U, Toft P (2020): Urine neutrophil gelatinase-associated lipocalin and urine output as predictors of the successful discontinuation of continuous renal replacement therapy in critically ill patients with acute kidney injury. BMC Nephrol 21; 375.

207. Tolwani AJ, Campbell RC, Stofan BS, Lai KR, Oster RA, Wille KM (2008): Standard versus high-dose CVVHDF for ICU-related acute renal failure. Journal of the American Society of Nephrology : JASN 19; 1233‐1238.

208. Tourneur JM, Weissbrich C, Putensen C, Hilbert T (2019): Feasibility of a protocol to wean patients from continuous renal replacement therapy: A retrospective pilot observation. J Crit Care 53; 236-243.

209. Truche AS, Darmon M, Bailly S, Clec'h C, Dupuis C, Misset B, Azoulay E, Schwebel C, Bouadma L, Kallel H et al. (2016): continuous renal replacement therapy versus intermittent hemodialysis in intensive care patients: impact on mortality and renal recovery. intensive Care Med 42; 1408-1417.

210. Tsujimoto H, Tsujimoto Y, Nakata Y, Fujii T, Takahashi S, Akazawa M, Kataoka Y (2020): Pharmacological interventions for preventing clotting of extracorporeal circuits during continuous renal replacement therapy. Cochrane Database Syst Rev 12; Cd012467.

211. Uchino S, Bellomo R, Morimatsu H, Morgera S, Schetz M, Tan I, Bouman C, Macedo E, Gibney N, Tolwani A et al. (2009): Discontinuation of continuous renal replacement therapy: a post hoc analysis of a prospective multicenter observational study. Crit Care Med 37; 2576-2582.

212. Uehlinger DE, Jakob SM, Ferrari P, Eichelberger M, Huynh-Do U, Marti HP, Mohaupt MG, Vogt B, Rothen HU, Regli B et al. (2005): Comparison of continuous and intermittent renal replacement therapy for acute renal failure. Nephrology, dialysis, transplantation 20; 1630‐1637.

213. Uehlinger DE, Jakob SM, Ferrari P, Eichelberger M, Huynh-Do U, Marti HP, Mohaupt MG, Vogt B, Rothen HU, Regli B et al. (2005): Comparison of continuous and intermittent renal replacement therapy for acute renal failure. Nephrol Dial Transplant 20; 1630-1637.

214. Van Bommel EF, Kalmeijer MD, Ponssen HH (2000): Treatment of life-threatening lithium toxicity with high-volume continuous venovenous hemofiltration. Am J Nephrol 20; 408-411.

215. Van Der Voort PH, Boerma EC, Koopmans M, Zandberg M, De Ruiter J, Gerritsen RT, Egbers PH, Kingma WP, Kuiper MA (2009): Furosemide does not improve renal recovery after hemofiltration for acute renal failure in critically ill patients: a double blind randomized controlled trial. Critical care medicine 37; 533‐538.

216. Van Wert R, Friedrich JO, Scales DC, Wald R, Adhikari NKJ (2010): High-dose renal replacement therapy for acute kidney injury: Systematic review and meta-analysis. Critical Care Medicine 38; 1360-1369.

217. Vardakas KZ, Voulgaris GL, Maliaros A, Samonis G, Falagas ME (2018): Prolonged versus short-term intravenous infusion of antipseudomonal β-lactams for patients with sepsis: a systematic review and meta-analysis of randomised trials. Lancet Infect Dis 18; 108-120.

218. Venkatasubba Rao CP, Bershad EM, Calvillo E, Maldonado N, Damani R, Mandayam S, Suarez JI (2018): Real-time Noninvasive Monitoring of Intracranial Fluid Shifts During Dialysis Using Volumetric Integral Phase-Shift Spectroscopy (VIPS): A Proof-of-Concept Study. Neurocrit Care 28; 117-126.

219. Viallet N, Brunot V, Kuster N, Daubin D, Besnard N, Platon L, Buzançais A, Larcher R, Jonquet O, Klouche K (2016): Daily urinary creatinine predicts the weaning of renal replacement therapy in ICU acute kidney injury patients. Annals of intensive Care 6; 71.

220. Vinsonneau C, Camus C, Combes A, Costa De Beauregard MA, Klouche K, Boulain T, Pallot JL, Chiche JD, Taupin P, Landais P et al. (2006): continuous venovenous haemodiafiltration versus intermittent haemodialysis for acute renal failure in patients with multiple-organ dysfunction syndrome: a multicentre randomised trial. Lancet 368; 379-385.

221. Vives M, Karkouti K, Rao V, Chan CT, Wijeysundera DN (2022): Acute kidney injury after cardiac surgery: Sustained low efficiency compared to continuous renal replacement therapy. J Clin Anesth 77; 110642.

222. Von Groote T, Albert F, Meersch M, Koch R, Porschen C, Hartmann O, Bergmann D, Pickkers P, Zarbock A (2022): Proenkephalin A 119-159 predicts early and successful liberation from renal replacement therapy in critically ill patients with acute kidney injury: a post hoc analysis of the ELAIN trial. Crit Care 26; 333.

223. Waineo MF, Kuhn TC, Brown DL (2015): The pharmacokinetic/pharmacodynamic rationale for administering vancomycin via continuous infusion. 40; 259-265.

224. Wald R, Adhikari NK, Smith OM, Weir MA, Pope K, Cohen A, Thorpe K, Mcintyre L, Lamontagne F, Soth M et al. (2015): Comparison of standard and accelerated initiation of renal replacement therapy in acute kidney injury. Kidney Int 88; 897-904.

225. Wald R, Gaudry S, Da Costa BR, Adhikari NKJ, Bellomo R, Du B, Gallagher MP, Hoste EA, Lamontagne F, Joannidis M et al. (2023): Initiation of continuous renal replacement therapy versus intermittent hemodialysis in critically ill patients with severe acute kidney injury: a secondary analysis of STARRT-AKI trial. intensive care medicine; 1305-1316.

226. Wald R, Shariff SZ, Adhikari NK, Bagshaw SM, Burns KE, Friedrich JO, Garg AX, Harel Z, Kitchlu A, Ray JG (2014): The association between renal replacement therapy modality and long-term outcomes among critically ill adults with acute kidney injury: a retrospective cohort study*. Crit Care Med 42; 868-877.

227. Wang H, Li L, Chu Q, Wang Y, Li Z, Zhang W, Li L, He L, Ai Y (2016): Early initiation of renal replacement treatment in patients with acute kidney injury A systematic review and meta-analysis. 95; e5434.

228. Wang Y, Gallagher M, Li Q, Lo S, Cass A, Finfer S, Myburgh J, Bouman C, Faulhaber-Walter R, Kellum JA et al. (2018): Renal replacement therapy intensity for acute kidney injury and recovery to dialysis independence: a systematic review and individual patient data meta-analysis. Nephrol Dial Transplant 33; 1017-1024.

229. Weidhase L, De Fallois J, Haußig E, Kaiser T, Mende M, Petros S (2020): Myoglobin clearance with continuous veno-venous hemodialysis using high cutoff dialyzer versus continuous veno-venous hemodiafiltration using high-flux dialyzer: a prospective randomized controlled trial. Crit Care 24; 644.

230. Wierstra BT, Kadri S, Alomar S, Burbano X, Barrisford GW, Kao RLC (2016): The impact of "early" versus "late" initiation of renal replacement therapy in critical care patients with acute kidney injury: A systematic review and evidence synthesis. 20; 1-13.

231. Wu B, Yan W, Li X, Kong X, Yu X, Zhu Y, Xing C, Mao H (2017): Initiation and Cessation Timing of Renal Replacement Therapy in Patients with Type 1 Cardiorenal Syndrome: An Observational Study. Cardiorenal Med 7; 118-127.

232. Wu CC, Su YC, Wu KS, Wu TH, Yang CS (2021): Loading dose and efficacy of continuous or extended infusion of beta-lactams compared with intermittent administration in patients with critical illnesses: A subgroup meta-analysis and meta-regression analysis. J Clin Pharm Ther 46; 424-432.

233. Wu VC, Huang TM, Shiao CC, Lai CF, Tsai PR, Wang WJ, Huang HY, Wang KC, Ko WJ, Wu KD et al. (2013): The hemodynamic effects during sustained low-efficiency dialysis versus continuous veno-venous hemofiltration for uremic patients with brain hemorrhage: a crossover study Clinical article. J Neurosurg 119; 1288-1295.

234. Xiao L, Jia L, Li R, Zhang Y, Ji H, Faramand A (2019): Early versus late initiation of renal replacement therapy for acute kidney injury in critically ill patients: A systematic review and meta-analysis. 14;

235. Xu Q, Jiang B, Li J, Lu W, Li J (2022): Comparison of filter life span and solute removal during continuous renal replacement therapy: convection versus diffusion - A randomized controlled trial. Ther Apher Dial 26; 1030-1039.

236. Xu Y, Gao J, Zheng X, Zhong B, Na Y, Wei J (2017): Timing of initiation of renal replacement therapy for acute kidney injury: a systematic review and meta-analysis of randomized-controlled trials. Clin Exp Nephrol 21; 552-562.

237. Yang T, Sun S, Lin L, Han M, Liu Q, Zeng X, Zhao Y, Li Y, Su B, Huang S et al. (2017): Predictive Factors Upon Discontinuation of Renal Replacement Therapy for Long-Term Chronic Dialysis and Death in Acute Kidney Injury Patients. Artif Organs 41; 1127-1134.

238. Yang T, Sun S, Zhao Y, Liu Q, Han M, Lin L, Su B, Huang S, Yang L (2018): Biomarkers upon discontinuation of renal replacement therapy predict 60-day survival and renal recovery in critically ill patients with acute kidney injury. Hemodialysis International 22; 56-65.

239. Yang XM, Tu GW, Zheng JL, Shen B, Ma GG, Hao GW, Gao J, Luo Z (2017): A comparison of early versus late initiation of renal replacement therapy for acute kidney injury in critically ill patients: An updated systematic review and meta-analysis of randomized controlled trials. BMC Nephrology 18; 264.

240. Ye Z, Wang Y, Ge L, Guyatt GH, Collister D, Alhazzani W, Bagshaw SM, Belley-Cote EP, Fang F, Hou L et al. (2021): Comparing Renal Replacement Therapy Modalities in Critically Ill Patients With Acute Kidney Injury: A Systematic Review and Network Meta-Analysis. Crit Care Explor 3; e0399.

241. Yin F, Zhang F, Liu S, Ning B (2020): The therapeutic effect of high-volume hemofiltration on sepsis: a systematic review and meta-analysis. Ann Transl Med 8; 488.

242. Yoshida T, Matsuura R, Komaru Y, Miyamoto Y, Yoshimoto K, Hamasaki Y, Noiri E, Morimura N, Nangaku M, Doi K (2019): Kinetic estimated glomerular filtration rate as a predictor of successful continuous renal replacement therapy discontinuation. Nephrology (Carlton) 24; 287-293.

243. Yu Z, Pang X, Wu X, Shan C, Jiang S (2018): Clinical outcomes of prolonged infusion (extended infusion or continuous infusion) versus intermittent bolus of meropenem in severe infection: A meta-analysis. PLoS One 13; e0201667.

244. Yusuf E, Spapen H, Piérard D (2014): Prolonged vs intermittent infusion of piperacillin/tazobactam in critically ill patients: a narrative and systematic review. J Crit Care 29; 1089-1095.

245. Zarbock A, Kellum JA, Schmidt C, Van Aken H, Wempe C, Pavenstadt H, Boanta A, Gerss J, Meersch M (2016): Effect of early vs delayed initiation of renal replacement therapy on mortality in critically ill patients with acute kidney injury: the elain randomized clinical trial. JAMA - journal of the american medical association 315; 2190‐2199.

246. Zha J, Li C, Cheng G, Huang L, Bai Z, Fang C (2019): The efficacy of renal replacement therapy strategies for septic-acute kidney injury: A PRISMA-compliant network meta-analysis. Medicine (Baltimore) 98; e15257.

247. Zhang K, Zhang H, Zhao C, Hu Z, Shang J, Chen Y, Huo Y, Zhao C, Li B, Guo S (2023): The furosemide stress test predicts the timing of continuous renal replacement therapy initiation in critically ill patients with acute kidney injury: a double-blind prospective intervention cohort study. European journal of medical research 28; 149.

248. Zhang L, Chen D, Tang X, Li P, Zhang Y, Tao Y (2020): Timing of initiation of renal replacement therapy in acute kidney injury: an updated meta-analysis of randomized controlled trials. Ren Fail 42; 77-88.

249. Zhang L, Yang J, Eastwood GM, Zhu G, Tanaka A, Bellomo R (2015): Extended Daily Dialysis Versus continuous Renal Replacement Therapy for Acute Kidney Injury: A Meta-analysis. Am J Kidney Dis 66; 322-330.

250. Zhang P, Yang Y, Lv R, Zhang Y, Xie W, Chen J (2012): Effect of the intensity of continuous renal replacement therapy in patients with sepsis and acute kidney injury: a single-center randomized clinical trial. Nephrol Dial Transplant 27; 967-973.

251. Zhang W, Bai M, Yu Y, Li L, Zhao L, Sun S, Chen X (2019): Safety and efficacy of regional citrate anticoagulation for continuous renal replacement therapy in liver failure patients: a systematic review and meta-analysis. Crit Care 23; 22.

252. Zhang Z, Xu X, Ni H, Jin N (2012): Serum cystatin C is associated with renal function recovery in critically ill patients undergoing continuous renal replacement therapy. Nephron Clin Pract 122; 86-92.

253. Zhao YY, Chen YF (2020): Effect of renal replacement therapy modalities on renal recovery and mortality for acute kidney injury: A PRISMA-compliant systematic review and meta-analysis. Semin Dialysis 33; 127-132.

254. Zhongheng Z, Xiao X, Hongyang Z (2010): intensive- vs less-intensive-dose continuous renal replacement therapy for the intensive care unit-related acute kidney injury: A meta-analysis and systematic review. Journal of Critical Care 25; 595-600.

255. Zhou F, Song Q, Peng Z, Pan L, Kang H, Tang S, Yue H, Liu H, Xie F (2011): Effects of continuous venous-venous hemofiltration on heat stroke patients: a retrospective study. J Trauma 71; 1562-1568.

256. Zhou X, Dong P, Pan J, Wang H, Xu Z, Chen B (2021): Renal replacement therapy modality in critically ill patients with acute kidney injury - A network meta-analysis of randomized controlled trials. J Crit Care 64; 82-90.

257. Zhou Z, Liu C, Yang Y, Wang F, Zhang L, Fu P (2023): Anticoagulation options for continuous renal replacement therapy in critically ill patients: a systematic review and network meta-analysis of randomized controlled trials. Crit Care 27; 222.

258. Zhu LL, Zhou Q (2018): Optimal infusion rate in antimicrobial therapy: Explosion of evidence in the last five years. 11; 1105-1117.
