# Supplementary material for: Multidisciplinary guidelines on renal replacement therapy in intensive care medicine
Source: Crit Care. 2026 Jan 14;30:46. doi: 10.1186/s13054-025-05817-6 (PMC12849416; doi:10.1186/s13054-025-05817-6)
Supplement: Supplementary file 2 — Supplementary Material 2. [file 13054_2025_5817_MOESM2_ESM.docx]

Supplementary Material

Multidisciplinary Guidelines on Renal Replacement Therapy in Intensive Care Medicine

TABLE OF CONTENTS PAGE

[Further recommendations/statements 2](#_Toc215084045)

[1. Start of Renal Replacement Therapy 2](#_Toc215084046)

[2. Modality (diffusion / convection) of Renal Replacement Therapy 2](#_Toc215084047)

[3. Continuous and intermittent Renal Replacement Therapy 3](#_Toc215084048)

[4. Anticoagulation for Renal Replacement Therapy 5](#_Toc215084049)

[5. Dose of Renal Replacement Therapy 6](#_Toc215084050)

[6. Pharmacology in Renal Replacement Therapy 7](#_Toc215084051)

[Elimination of toxins 10](#_Toc215084052)

[Lithium Intoxication 12](#_Toc215084053)

[7. Stopping Renal Replacement Therapy 13](#_Toc215084054)

[References 16](#_Toc215084055)

# Further recommendations/statements

## Start of Renal Replacement Therapy

| e1.1- Adverse effects of RRT should be closely monitored both clinically and biochemically for all modalities of RRT. | Expert consensus |
| --- | --- |

In principle, the use of RRT can be associated with side effects such as hemodynamic instability, vasopressor requirements, organ hypoperfusion, and cardiac arrhythmia. This is particularly true given that the patients involved are often in a clinically unstable situation. Starting RRT treatment early with a longer treatment period can potentially aggravate complications, such as hypophosphatemia, coagulation disorders, thrombocytopenia, hypothermia, and reduced drug levels.

The rate of complications after early or late initiation of RRT was reported in 7 studies [1-7]. In the Cochrane analysis by Fayad 2022 *[8]*, a RR of 1.23 (95% CI 0.90-1.68; I^2^=91%; 5 studies, 3864 patients) was calculated. The evidence was categorized as very low due to considerable imprecision and serious inconsistencies.

Early initiation of extracorporeal RRT showed a statistically higher risk of hypophosphatemia (RR 1.80, 95% CI 1.33-2.44; 1 study, 2927 patients), hypotension (RR 1.54, 95% CI 1.29-1.85; 5 studies, 3864 patients), for cardiac arrhythmias (RR 1.35, 95% CI 1.04-1.75; 6 studies, 4483 patients), and for infections (RR 1.33, 95% CI 1.00-1.77; 5 studies, 4252 patients). There was also evidence that *myocardial stunning* can occur regardless of early or late RRT onset *[9]*.

The effects on bleeding complications (RR 0.91; 95% CI 0.73-1.18; 6 studies, 4358 patients) and the risk of thrombocytopenia (RR 1.03; 95% CI 0.20-5.35; 1 study, 106 patients) due to early use of RRT remained unclear due to imprecision and a small number of cases.

## Modality (diffusion / convection) of Renal Replacement Therapy

| e2.1- No recommendation can be provided on whether diffusion or convection should be preferred in relation to the risk of elevated intracranial pressure. | 0  ⊕⊝⊝⊝ |
| --- | --- |

The high efficiency of diffusive procedures with a rapid displacement of osmolytes such as sodium, glucose or urea holds the potential risk of disequilibrium in the event of blood-brain barrier disorders. Johannson 2017*[10]* investigated the effect of IHD and IHDF and could not observe any differences in the cerebral water shift on MRI. The total brain volume changed by 1.8% (±1.7%) or 18.7 ml (±17.4 ml) under IHD and by +2.0% (±0.9%) or 22.3 ml (±10.7 ml) under predilution IHDF. Okada 2013 *[11]* compared short hemodialysis and hemofiltration sessions in 13 CKD patients with acute brain injury. They used additional glycerol infusions to better adjust changes in osmolality during RRT. A recommendation in favor of one of the two procedures cannot be derived from present data.

## Continuous and intermittent Renal Replacement Therapy

| e3.1-Given the current evidence, no recommendation can be provided in favor of continuous versus intermittent RRT with regard to renal recovery as an outcome. | 0 ⊕⊕⊝⊝ |
| --- | --- |

The question of differences in renal recovery from AKI after IHD or CRRT was analyzed in 10 systematic reviews from 2002 to 2021. When only data from RCTs were used, no systematic review was able to calculate a difference in renal recovery or dialysis requirement. 4/10 systematic reviews found a moderate benefit for CRRT when observational and retrospective data were included in the analysis. Here, the underlying number of patients included in the calculations was significantly higher than in the RCTs. Schoenfelder 2017 [12] analyzed 1,870 patients in RCT analyses (no effect) and 15,689 patients in the non-controlled study analyses (advantages of CRRT).

To date, the large posthoc analyses of the ATN, RENAL and STARRT-AKI studies have not been included in these systematic reviews. In the secondary analyses of the ATN and RENAL trials [13] with 2,542 patients, no difference in mortality was found. However, the ATN study cohort showed better renal recovery after CRRT. This finding is limited since an increased cardiovascular SOFA score automatically led to allocation to the CRRT group. Ultimately, 85.5% of patients were in the CRRT group because there was no randomization for continuous or intermittent procedures.

Wald 2023 *[2]* conducted a posthoc analysis of the large STARRT-AKI trial. In this study, 2,196 patients were randomized to an early or later start of RRT. It showed a significantly higher rate of renal recovery with CRRT (RRT dependency at day 90: OR 0.61 (95% CI 0.39-0.94)). However, mortality alone was not significantly different, so that renal recovery was the driving signal. This study was not designed for the endpoint IHD vs. CRRT and therefore was not randomized for effects of different RRT modalities. Instead, the allocation to a continuous or intermittent procedure was carried out unblinded according to the preference of the centers. Furthermore, there was a clear group imbalance in the cohorts (1,590 patients with CRRT, 606 patients with IHD).

In addition, regional differences may be difficult to define influencing factors, as IHD was comparatively more common in French centers *[14]*. Bonnassieux 2018 *[15]* analyzed in a large retrospective database analysis with 25,750 data sets from France and calculated a better renal recovery after CRRT with an OR of 0.91 (95% CI 0.83-0.99; p = 0.03). In contrast, the observational OUTCOMEREA study *[16]*, also from France, was unable to calculate any differences in a composite endpoint of mortality and renal recovery.

A recent large retrospective database analysis of an insurance collective by *[17]* calculated a significantly lower 90-day dialysis dependency under CRRT (4.9% vs. 7.4%; p = 0.006; OR 0.68 (95% CI 0.47-0.97), p = 0.03). The intermittent and continuous group were heterogeneous since they differed in terms of age, comorbidities, disease severity, socioeconomic status and ICU length of stay, in some cases significantly.

The current data situation is not conclusive. Controlled studies showed no differences. In contrast, large retrospective analyses revealed advantages regarding renal recovery under continuous procedures.

| e3.2- For safety reasons, institutions should establish a procedural protocol for nursing and physiotherapeutic management of patients receiving RRT, consistent with DIVI Quality Indicator IX (Early Mobilization). | Expert consensus |
| --- | --- |
| e3.3- Multiprofessional education and training programs on the specialized nursing care of critically ill patients undergoing RRT should be implemented. | Expert consensus |

Abbreviation: DIVI= German Interdisciplinary Society of Intensive Care Medicine

Early mobilization is an essential part of intensive care and treatment, even for patients with AKI who require RRT. However, the presence of large-french blood lines, as well as being tethered to the dialysis machine, can theoretically hinder mobilization. Fear of dislocation of the dialysis catheters can hinder the regular implementation of early mobilization in intensive care *[18]*. According to the S3 guideline *Positioning therapy and mobilization of critically ill patients in intensive care units [19]*, a femoral dialysis catheter should not prevent early mobilization. Unlike continuous procedures, intermittent procedures allow mobilization during dialysis-free intervals. However, it can also be performed safely during CRRT and is not contraindicated *[20-22]*. It should be emphasized that the effort and feasibility of mobilization are perceived differently by different professional groups *[23]*. A protocol-based approach and internal hospital safety guidelines can be helpful in establishing a pragmatic and safe procedure [24, 25]. Interprofessional training and practical training according to a standardized curriculum are recommended *[26, Chen, 2023 #317]*.

The guideline group recommends that clinics and facilities create, implement and consistently adhere to clinic-specific and interdisciplinary standard operating procedures (SOPs) for the performance of RRT. These SOPs define the requirements for the qualifications, competences and training of the nursing staff involved as well as the specific procedures, competences and responsibilities for continuous and intermittent procedures. In a study by Chen 2023 *[27]*, the effect of a specialized nursing quality control team in an intensive care unit on CRRT was analyzed. Compared to the control group, the intervention group showed a reduction in unplanned events. This included improved adherence to continuous treatment (>24 hours) and planned downtime (72 hours). Furthermore, the intervention group showed lower daily hemodialysis costs per patient, a longer useful life of individual filters, lower rates of unplanned extubations and bloodstream infections. Patients also reported higher satisfaction with their care.

## Anticoagulation for Renal Replacement Therapy

| e4.1-Regional citrate anticoagulation should be used only with regular monitoring of electrolyte levels (especially serum sodium and total and ionized calcium) and acid–base status (including blood pH, bicarbonate, and lactate). | Expert consensus |
| --- | --- |

Adverse events differ significantly between RCA and systemic heparin anticoagulation. The following pharmacochemical effects have been described when citrate is used:

- the chelation of divalent cations (calcium, magnesium and others) with a critical deficiency of these cations
- metabolization to bicarbonate and the associated alkalosis
- the intake of sodium with clinically significant hypernatremia, as it is a trisodium salt
- Citrate accumulation, which can lead to metabolic acidosis with an increased anion gap and hypercalcemia, or an increased calcium ratio (total calcium/ionized calcium), resulting from a citrate intake that exceeds its hepatic metabolism.

Therefore, during RCA, it is necessary to regularly check electrolytes and acid-base balance parameters. Failing to adequately respond to any deviations can lead to severe disturbances in these parameters, particularly serum calcium (hypo- or hypercalcemia) and in acid-base disorders.

Four systematic reviews [28-31] investigated the occurrence of hypocalcemia (<0.7 to 1.0 mmol/L) under RCA. The reviews consistently concluded that hypocalcemia occurred significantly more frequently with RCA.

Four previous systematic reviews [28-31] did not show an increase in metabolic alkalosis or acidosis with RCA compared to systemic heparin anticoagulation. On the other hand, the Cochrane analysis by Tsujimoto 2020 *[30]*, found more alkalosis with RCA. However, this systematic review did not consider the RICH study *[32]*, the largest RCT in this area to date. No data are available on other anticoagulation strategies.

The question of whether RCA reduces the rate of thrombocytopenia compared to systemic heparin anticoagulation has led to inconsistent results. The systematic reviews by Bai 2015 *[28]* and Liu 2016 *[31]* showed a lower rate of thrombocytopenia. However, Tsujimoto 2020 *[30]* and Jacobs 2023 *[29]* saw no difference.

RCA can be considered a relatively safe procedure thanks to the automated control systems of modern RRT systems. However, the high complexity and risk of metabolic imbalance require adequate training and use of published treatment protocols in order to use these systems safely. Therefore, we recommend that users receive adequate training to safely operate these systems with patients (high consensus, no level of evidence).

## Dose of Renal Replacement Therapy

| e5.1- The delivered mean dose of RRT should be recorded at least once daily. Clinical effectiveness should be evaluated and therapy adjusted on a daily basis according to clinical and laboratory variables, including electrolytes, acid–base balance, and urea. | Expert consensus |
| --- | --- |

The recommended dose is based on best available evidence. Nevertheless, it is not possible to determine a "correct" dose with absolute certainty, partly because the potential benefits of lower or optimized doses have not been investigated in RCTs. Therefore, it cannot be ruled out that a different, possibly lower, dose could improve clinical efficacy and the spectrum of side effects.

The prescribed dose usually differs from the actual delivered dose, as the dose depends on effective running time and is influenced by interruptions (e.g. transport, clotting, set-up times). In clinical routine, the prescribed dose is therefore selected at a slightly higher level (e.g. 25–30 ml/h/kg) to ensure the target dose of 20–25 ml/h/kg is achieved.

Our recommendations are primarily based on systematic reviews and the two large RCTsATN *[33]* and RENAL trial *[34]*. The ATN study showed an average of 21 hours of RRT per day. The remaining hours without treatment were the result of downtime (e.g. due to transport, filter changes due to clotting, etc.).

Higher-doses procedures have been shown to be associated with side effects like hypophosphatemia (*[33, 34]*). To this end, we recommend to continuously test for clinical effectiveness and to adjust therapy on a daily basis according to clinical and laboratory variables, including electrolytes, acid–base balance, and urea.

The same is valid for intermittent procedures, since its high efficiency and a greater risk for the development of a disequilibrium syndrome. Dialysis disequilibrium syndrome (DDS) refers to a range of symptoms that can occur due to the rapid elimination of osmotically active substances (e.g., urea). In the dynamic clinical picture of a critically ill patient, we recommend setting a dose target (dialysate flow, blood flow, duration) for each individual dialysis treatment (expert opinion).

## Pharmacology in Renal Replacement Therapy

| e6.1- To optimize anti-infective therapy, patient-related factors that influence pharmacokinetics (including body size, weight, residual renal function, protein status, and fluid overload) should be evaluated when adjusting drug dosing in patients undergoing RRT. | Expert consensus |
| --- | --- |
| e6.2- To optimize anti-infective therapy, pharmacokinetic and pharmacodynamic characteristics of the drug—such as hydrophilicity or lipophilicity, volume of distribution, renal elimination fraction, half-life, and protein binding—should be considered when adjusting dosing in patients receiving RRT. | Expert consensus |

The starting dose is defined as the initial dose administered. It should be based on generally accepted pharmacokinetic rules according to the patient's apparent volume of distribution. In general, the volume of distribution is a variable that indicates how widely a drug is distributed outside the central compartment of the body. Therefore, it correlates with the drug's concentration in the plasma. However, this is an apparent volume of distribution because its calculation is based on the assumption of uniform drug distribution, whereas individual compartments have different distributions. Therefore, the plasma concentration of a drug is equal to the drug's amount in the body/volume of distribution. Consequently, a change in the volume of distribution (e.g., increased hypoproteinemia or edema) affects the plasma concentration, resulting in a redistribution of the dose according to the existing volume of distribution. A high volume of distribution usually correlates with a lipophilic drug's ability to leave the blood compartment, resulting in a low blood level (fluoroquinolones, metronidazole, rifampicin, linezolid, and chloramphenicol). In contrast, hydrophilic antibiotics generally achieve higher blood levels and have a lower volume of distribution β-lactams (penicillins, cephalosporins, carbapenems, and monobactams), aminoglycosides, and vancomycin).

In contrast to the initial dose of an anti-infective agent, which is based on volume of distribution, the maintenance dose is determined according to a drug's clearance. After therapy begins, once the drug has been effectively saturated, a balance between re-dosing and clearance is required to maintain an effective level of activity.

| e6.3- Owing to the high interindividual pharmacokinetic variability of commonly used anti-infective agents, maintenance dosing should be guided by evidence-based, standardized reference tables, and therapeutic drug monitoring (TDM) should be performed whenever feasible. | Expert consensus |
| --- | --- |
| e6.4- The timing of TDM measurements should be adapted to the mode of drug administration (e.g., short infusion, prolonged, or continuous infusion) and should follow standardized, reproducible sampling protocols. | Expert consensus |
| e6.5- The results of TDM should be evaluated against predefined pharmacokinetic/pharmacodynamic target attainment goals with clearly defined target ranges. | Expert consensus |

Determining PK/PD target attainment for antibiotic therapy requires that the measurement time point be defined and the blood level to be achieved. In studies, the TDM sampling time typically corresponds to the middle or end of a dosing interval. In clinical practice, measurement at the end of the dosing interval (i.e., before the next dose) is usually preferred for reasons of practicality. After 3-5 half-lives, we can assume that a steady state has been reached in the blood and tissue, as long as dosage and organ function have not changed significantly. Once the TDM sampling time has been determined, the minimum concentration of the antibiotic in the blood must be defined.

In the context of short infusion administration, a target trough level (free concentration, fc(min)) > 1 x MIC is considered appropriate. In the context of continuous administration, a target level of 2-6 x MIC is considered appropriate. This target level depends on the severity of the disease and the pathogen identified [35-38].

The evaluation of the effects of TDM for intensive care patients undergoing RRT is complicated by the fact that many studies used AKI or RRT as exclusion criteria. These studies are probably not appropriate for patients with AKI. One exception is the large retrospective study by Richter 2019 [39] that also included patients undergoing RRT. Richter 2019 found that 10.1% of patients in the entire cohort (with and without RRT) were underdosed with piperacillin/tazobactam at the initial empirical dose.

The TARGET RCT [40] investigated the effects of TDM-based individual dosing of piperacillin under continuous administration in 254 patients. Approximately one quarter of the included patients received RRT. The SOFA score as the primary endpoint (7.9 with TDM vs. 8.2 without TDM) and 28-day mortality (21.6% vs. 25.8%, RR 0.8 (95% CI, 0.5–1.3), p = 0.44) and clinical cure (OR 1.9 (95% CI, 0.5–6.2); p = 0.30) were not significantly different. On the other hand, PK/PD targets were significantly better achieved with TDM (37.3% vs. 14.6%; OR 4.5 (95% CI, 2.9–6.9), p < 0.001).

Another finding was that the 28-day mortality rate was significantly higher for patients with piperacillin concentrations greater than 96 mg/L compared to those with concentrations between 32 and 64 mg/L (4/48) (33.7% vs. 8.3%, OR 4.21 (95% CI, 1.4–12.5), p = 0.01) or 64–96 mg/L (12/61) (33.7% vs. 19.7%, OR 2.5 (95% CI, 1.1–5.8), p = 0.03). According to Hagel 2022 [40], this was most likely due to reduced renal drug elimination and subsequent accumulation in the most severely ill patients.

In the multicenter Dolphin study [41], the authors investigated the effects of a dosing algorithm that used TDM together with pharmacometric modeling of β-lactams. They found that model-based, individualized dosing did not significantly affect the outcomes of critically ill patients compared to standard dosing. Approximately 15% of the patients studied required RRT. The study was criticized for its methodology due to wide PK/PD targets chosen (>MIC<10xMIC), the highly biased pharmacokinetic model used, and the small sample size [42].

In the systematic review by Sanz-Codina (2023 #1001), the results from 10 studies evaluating the effects of TDM were evaluated. Mortality and clinical cure were not significantly improved with TDM, with RR of 0.86 (95% CI 0.71-1.05) and 1.33 (95% CI 0.94-1.33), respectively. Conversely, PK/PD target attainment using TDM was significantly better, with an RR of 1.41 (95% CI, 1.13–1.76), as was reduced nephrotoxicity, with an RR of 0.55 (95% CI, 0.31–0.97). It should be noted, however, that the Dolphin trial was weighted at 32.1% in the systematic review, thus contributing significantly to the negative signal of the review.

Luxton 2022 [43] cited 8 studies with positive effects and 7 studies in a further systematic review without positive effects of TDM on PK/PD target attainment. Therefore, Luxton 2022 [43] concluded that, based on the available, partly contradictory evidence, it would not be valid to perform a meta-analysis.

The question of the effect of TDM in patients undergoing CRRT and intermittent RRT was investigated in the systematic review by Matusik 2022 [38]. Matusik, 2022 concluded that the very high variability of antibiotic levels justifies recommending TDM for aminoglycosides, ß-lactams, glycopeptides, linezolid, and colistin, and that it can be considered for tigecycline, daptomycin, and fluoroquinolones.

Recent studies have investigated the role of TDM in linezolid, as standard dosing also led to very different target levels and increased toxicity was observed, particularly in patients undergoing RRT. Thrombocytopenia occurred significantly more frequently in patients with AKI (42.9% versus 16.8%; P < 0.001), adjusted HR 2.37; 95% CI 1.52 - 3.68) [44]. In this study, age, body surface area, and eGFR were identified as covariates of linezolid clearance. A systematic review by Rao 2020 [45] concluded that TDM for linezolid is a helpful tool for treating specific populations more safely, such as children, patients with kidney damage and RRT, and patients with co-medications that interact with linezolid.

Although there is insufficient data on the effects of TDM in patients undergoing RRT, the high interindividual variability of antibiotic concentrations in the blood suggests the use of TDM. Due to the significant probability of incorrect dosing in patients with multiple organ failure and renal replacement therapy, we recommend, based on a consensus and limited evidence, that TDM be performed whenever possible for critical antibiotics in life-threatening infections.

### Elimination of toxins

| e6.6- For effective drug clearance, both endogenous and extracorporeal clearance should be evaluated. Initiation of RRT may be appropriate when endogenous clearance is insufficient (e.g., AKI, CKD) and clinically relevant extracorporeal clearance can be achieved. | Expert consensus |
| --- | --- |
| e6.7- To achieve effective extracorporeal clearance, the molecule should (A) be sufficiently small in size, (B) have low to moderate protein binding to allow passage through the filter membrane, and (C) possess a sufficiently low volume of distribution to ensure adequate availability within the plasma compartment. | Expert consensus |

In order to eliminate a toxin, it must first be clarified to what extent a toxin can be effectively eliminated endogenously or by an extracorporeal procedure. To ensure sufficient treatment efficiency, more than 30% of a dialyzable substance should be removed (10–30% indicating moderate efficiency), or the ratio of extracorporeal clearance to total clearance should exceed 75% (50–75% indicating moderate efficiency). In order to dialyze a toxin, it must have a low molecular weight and low protein binding. Protein binding may not reflect binding under overdose conditions since saturation of the protein-binding capacity can result in decreased protein binding and increased unbound fractions.

The key criteria for the extracorporeal elimination of toxins via RRT are as follows:

1. **Low molecular weight**, allowing passage through dialysis membrane pores; molecular weight must be below the membrane cutoff threshold.
2. **Low protein binding** (<80%), as only the unbound fraction can cross the membrane.
3. **Low volume of distribution** (generally <0.7–1.0 L/kg), ensuring availability of the toxin in the intravascular compartment for extracorporeal removal.

The EXTRIP Workgroup (https://www.extrip-workgroup.org) is an international consortium of experts that provides an evidence-based evaluation of toxins and their suitability for extracorporeal removal. We recommend consulting these resources when considering an extracorporeal detoxification procedure.

For effective renal clearance, a drug should have a Q₀ fraction of less than 0.5 (i.e. more than 50% should be eliminated by the kidneys) and should therefore be primarily cleared by the kidneys.

If kidney function is not impaired, endogenous renal clearance is usually sufficient for substances that are eliminated by the kidneys, and an additional extracorporeal modality requires a strong clinical indication. One such indication may be high nephrotoxicity, where there is a concern that intrarenal accumulation of the substance may cause severe acute kidney injury.

The physicochemical properties of the substance must permit passage through the membrane. Standard high-flux dialysis membranes generally have a molecular weight cut-off of 10–20 kDa. For larger molecules, membranes with higher cut-off thresholds must be selected.

Following effective clearance from the intravascular compartment and discontinuation of RRT, a rebound phenomenon must be anticipated for many toxins. Follow-up concentration monitoring should therefore be planned accordingly (see also the lithium section).

Substantial expertise in extracorporeal clearance modalities is required to perform extracorporeal detoxification. For optimal patient management, we recommend the involvement of trained specialists, such as nephrologists, clinical pharmacologists and toxicologists.

### Lithium Intoxication

| e6.8- RRT should be initiated in cases of life-threatening lithium intoxication or when clinical indications such as impaired consciousness, seizures, or severe cardiac arrhythmias are present. | A  ⊕⊝⊝⊝ |
| --- | --- |
| e6.9- In patients with impaired renal function, RRT should be initiated when the serum lithium concentration exceeds 4 mmol/L. | A  ⊕⊝⊝⊝ |
| e6.10- RRT should be considered when the serum lithium concentration exceeds 5 mmol/L, when the patient shows signs of confusion attributable to lithium intoxication, or when it is expected to take more than 36 hours for the lithium level to fall below 1 mmol/L. | B  ⊕⊝⊝⊝ |
| e6.11- RRT for lithium intoxication may be discontinued when the serum lithium concentration is below 1 mmol/L, when there is clinically significant improvement in the patient’s condition, or after approximately 6 hours of dialysis have been completed. | 0  ⊕⊝⊝⊝ |
| e6.12- Serum lithium concentration should be remeasured 12 hours after discontinuation of RRT to detect potential rebound increases. | A  ⊕⊝⊝⊝ |
| e6.13- IHD should be preferred as the primary method for lithium elimination. If IHD is not available, CRRT may be used. Alternatively, prolonged intermittent dialysis can be considered. | B  ⊕⊝⊝⊝ |
| e6.14- For rebound therapy, intermittent, prolonged intermittent, or continuous RRT modalities may all be employed. | 0  ⊕⊝⊝⊝ |

Lithium is a small chemical element with an atomic weight of 6.94. It has properties similar to sodium. Overdose or intoxication can lead to serious, life-threatening poisoning. Since approximately 80% of lithium is reabsorbed in the proximal tubule, endogenous clearance is a maximum of 25% of GFR, i.e., approximately 20-40 mL/min. Ultimately, lithium is eliminated almost exclusively by the kidneys. Since an extracorporeal procedure can eliminate the small molecule very well due to its lack of protein binding and low distribution volume, and since reabsorption does not occur, a renal replacement procedure is potentially more effective than the native kidneys for removing lithium.

For this reason, the EXTRIP (*EXtracorporeal TReatments In Poisoning*; www.extrip-workgroup.org) working group recommends the initiation of RRT in cases of severe poisoning with impaired renal function at lithium levels of 4 mmol/l or higher, or if there is a clinical indication such as impaired consciousness, convulsions, or life-threatening cardiac arrhythmias.

In 2015, the EXTRIP consortium published a systematic review and recommendations on extracorporeal therapy for lithium intoxication [46]. A Cochrane review, also published in 2015, listed the evidence available up to around 2014, but did not conduct a systematic review as no RCTs were available [47]. After 2015, a retrospective study from Sweden is worth mentioning, which performed RRT in 12 patients out of a total cohort of 91 patients with lithium poisoning [48]. Ten patients were primarily treated with IHD, two primarily with CRRT. Given the good evidence and a procedure that has now been clinically proven, the EXTRIP recommendation is therefore adopted here.

The endogenous lithium clearance (20-40 ml/min) is reported to be rather low in patients with normal renal function. In line with the high diffusive clearance of small molecules, an effective dialysis clearance of up to 160-180 ml/min has been reported [49-51]. A lower clearance of 19-64 ml/min has been measured in continuous procedures [46].

Although the intravascular space can be quickly cleared by dialysis, lithium can flow back from the interstitium into the vascular compartment once dialysis has ended. Therefore, it is important to repeatedly measure lithium levels after renal replacement therapy ends. If repeated procedures are necessary, the literature also describes the successful use of continuous procedures such as SLED or further IHDs.

## Stopping Renal Replacement Therapy

| e7.1- A period of 7 days may be used as a benchmark to define primary successful discontinuation of therapy. | Expert consensus |
| --- | --- |

Our systematic literature search identified 20 studies that provided information on the definition of successful discontinuation. The mean value across these studies was 5.95 days. Eight of the studies chose a period of seven days. Studies that retrospectively calculated the incidence of RRT within 90 days after discontinuation of primary therapy (usually 90 days) were not considered, as such a long period cannot be translated into clinical decision-making. Therefore, the consensus group considers a period of seven days to be a suitable measure for defining successful primary discontinuation.

| e7.2- Weaning protocols for RRT may be applied when discontinuing therapy. However, specific established protocols with defined variables cannot currently be recommended. | 0  ⊕⊝⊝⊝ |
| --- | --- |

Diuresis volume, concentration markers, and other biomarkers have been identified as surrogate parameters associated with the successful completion of RRT. In order to evaluate a set of clinical data, initial scoring systems have been proposed that could lead to greater predictive power. In recent years, five observational and retrospective studies have investigated various protocols and algorithms. Despite limited evidence, the studies demonstrated that specific protocols resulted in more reliable termination of RRT. However, an established protocol is still lacking [52, 53].

Baeg et al. 2021 [52] summarized four variables that correlated well with actual weaning success. These factors were a mean arterial pressure (MAP) of 50–78 mmHg, a urea nitrogen level of less than 35 mg/dL (±12.5 mmol/L), a potassium level of less than 4.1 mmol/L on the day of termination, and a urine output of greater than 300 mL the day after the end of RRT.

| e7.3- We recommend against the use of serum creatinine as a predictive marker for the discontinuation of RRT, given that serum creatinine levels are directly affected by the therapy itself. | Expert consensus |
| --- | --- |

Creatinine is not a reliable parameter because its measurement is influenced by both endogenous and machine clearance. The same may apply to cystatin C, which has been associated with increased weaning failure in several studies when levels are elevated. However, it is also significantly reduced in the blood by mechanical clearance [54, 55].

| e7.4- Outpatient medical follow-up should occur promptly after hospital discharge (within 2–4 weeks) in patients with reduced kidney function, including decreased GFR, following an episode of AKI. | Expert consensus |
| --- | --- |
| e7.5- Outpatient medical follow-up should also be conducted in patients with good recovery (GFR >60 mL/min) after an episode of acute kidney injury (AKI), typically within 3–6 months post-discharge. | Expert consensus |

AKI has been associated with an increased risk of long-term renal impairment [56]. A meta-analysis by Abdala 2021 [57] evaluated 17 studies that included clear definitions of AKI and CKD. The risk of developing CKD was significantly increased for non-surgical patients with an OR of 3.32 (95% CI 2.06-5.34) and for perioperative patients with an OR of 5.20 (95% CI 3.12-8.66). Overall, the OR was 4.31 (95% CI, 3.01–6.17).

The ADQI consensus group called for healthcare systems to establish structures and processes to adequately follow up patients after AKI [58]. If renal function remains impaired after discharge from the hospital, a follow-up visit should be scheduled within the following weeks. This will allow for the timely detection of potential complications or the initiation of preventive measures.

The guideline group believes that early detection of CKD is beneficial for affected patients from a clinical standpoint. This can include supportive therapies such as blood pressure control and treatment of calcium-phosphate balance disorders. Implementing an AKI aftercare structure in a post ICU setting would be beneficial.

# References

1. Bouman CS, Oudemans-Van Straaten HM, Tijssen JG, Zandstra DF, Kesecioglu J: **Effects of early high-volume continuous venovenous hemofiltration on survival and recovery of renal function in intensive care patients with acute renal failure: a prospective, randomized trial**. *Critical care medicine* 2002, **30**(10):2205‐2211.

2. Wald R, Gaudry S, da Costa BR, Adhikari NKJ, Bellomo R, Du B, Gallagher MP, Hoste EA, Lamontagne F, Joannidis M *et al*: **Initiation of continuous renal replacement therapy versus intermittent hemodialysis in critically ill patients with severe acute kidney injury: a secondary analysis of STARRT-AKI trial**. *Intensive care medicine* 2023:1305-1316.

3. Gaudry S, Hajage D, Schortgen F, Martin-Lefevre L, Pons B, Boulet E, Boyer A, Chevrel G, Lerolle N, Carpentier D *et al*: **Initiation Strategies for Renal-Replacement Therapy in the Intensive Care Unit**. *New England journal of medicine* 2016, **375**(2):122‐133.

4. Zarbock A, Kellum JA, Schmidt C, Van Aken H, Wempe C, Pavenstadt H, Boanta A, Gerss J, Meersch M: **Effect of early vs delayed initiation of renal replacement therapy on mortality in critically ill patients with acute kidney injury: the elain randomized clinical trial**. *JAMA - journal of the american medical association* 2016, **315**(20):2190‐2199.

5. Barbar SD, Clere-Jehl R, Bourredjem A, Hernu R, Montini F, Bruyère R, Lebert C, Bohé J, Badie J, Eraldi JP *et al*: **Timing of Renal-Replacement Therapy in Patients with Acute Kidney Injury and Sepsis**. *N Engl J Med* 2018, **379**(15):1431-1442.

6. Srisawat N, Laoveeravat P, Limphunudom P, Lumlertgul N, Peerapornratana S, Tiranathanagul K, Susantitaphong P, Praditpornsilpa K, Tungsanga K, Eiam-Ong S: **The effect of early renal replacement therapy guided by plasma neutrophil gelatinase associated lipocalin on outcome of acute kidney injury: A feasibility study**. *J Crit Care* 2018, **43**:36-41.

7. Bagshaw SM, Wald R, Adhikari NKJ, Bellomo R, da Costa BR, Dreyfuss D, Du B, Gallagher MP, Gaudry S, Hoste EA *et al*: **Timing of Initiation of Renal-Replacement Therapy in Acute Kidney Injury**. *N Engl J Med* 2020, **383**(3):240-251.

8. Fayad AI, Buamscha DG, Ciapponi A: **Timing of kidney replacement therapy initiation for acute kidney injury**. *Cochrane Database of Systematic Reviews* 2022(11).

9. Slessarev M, Salerno F, Ball IM, McIntyre CW: **Continuous renal replacement therapy is associated with acute cardiac stunning in critically ill patients**. *Hemodial Int* 2019, **23**(3):325-332.

10. Johansen N, Kjaergaard KD, Peters CD, Pedersen M, Jespersen B, Jensen JD: **Brain swelling during dialysis: A randomized trial comparing low-flux hemodialysis with pre-dilution hemodiafiltration***Clin Nephrol* 2017, **87 (2017)**(5):221-230.

11. Okada K, Abe M, Takashima H, Baba S, Taniguchi M, Suzuki H, Yoshida Y, Oikawa O, Maruyama N, Soma M: **Randomized trial of frequent low-efficiency and short hemodialysis/hemofiltration in hemodialysis patients with acute brain injury**. *Int J Artif Organs* 2013, **36**(11):793-802.

12. Schoenfelder T, Chen X, Bless HH: **Effects of continuous and intermittent renal replacement therapies among adult patients with acute kidney injury**. *GMS Health Technol Assess* 2017, **13**:Doc01.

13. Naorungroj T, Neto AS, Wang A, Gallagher M, Bellomo R: **Renal outcomes according to renal replacement therapy modality and treatment protocol in the ATN and RENAL trials**. *Crit Care* 2022, **26**(1):269.

14. Vaara ST, Serpa Neto A, Bellomo R, Adhikari NKJ, Dreyfuss D, Gallagher M, Gaudry S, Hoste E, Joannidis M, Pettila V *et al*: **Regional Practice Variation and Outcomes in the Standard Versus Accelerated Initiation of Renal Replacement Therapy in Acute Kidney Injury (STARRT-AKI) Trial: A Post Hoc Secondary Analysis**. *Crit Care Explor* 2024, **6**(2):e1053.

15. Bonnassieux M, Duclos A, Schneider AG, Schmidt A, Bénard S, Cancalon C, Joannes-Boyau O, Ichai C, Constantin JM, Lefrant JY *et al*: **Renal Replacement Therapy Modality in the ICU and Renal Recovery at Hospital Discharge**. *Crit Care Med* 2018, **46**(2):e102-e110.

16. Truche AS, Darmon M, Bailly S, Clec'h C, Dupuis C, Misset B, Azoulay E, Schwebel C, Bouadma L, Kallel H *et al*: **Continuous renal replacement therapy versus intermittent hemodialysis in intensive care patients: impact on mortality and renal recovery**. *Intensive Care Med* 2016, **42**(9):1408-1417.

17. Koyner JL, Mackey RH, Echeverri J, Rosenthal NA, Carabuena LA, Bronson-Lowe D, Harenski K, Neyra JA: **Initial renal replacement therapy (RRT) modality associates with 90-day postdischarge RRT dependence in critically ill AKI survivors**. *J Crit Care* 2024, **82**:154764.

18. Hermes C, Nydahl P, Grunow JJ, Schaller SJ: **[Positioning therapy for intensive care patients]**. *Dtsch Med Wochenschr* 2024, **149**(17):1028-1033.

19. Perme C, Nalty T, Winkelman C, Kenji Nawa R, Masud F: **Safety and Efficacy of Mobility Interventions in Patients with Femoral Catheters in the ICU: A Prospective Observational Study**. *Cardiopulm Phys Ther J* 2013, **24**(2):12-17.

20. Toonstra AL, Zanni JM, Sperati CJ, Nelliot A, Mantheiy E, Skinner EH, Needham DM: **Feasibility and Safety of Physical Therapy during Continuous Renal Replacement Therapy in the Intensive Care Unit**. *Ann Am Thorac Soc* 2016, **13**(5):699-704.

21. Mayer KP, Joseph-Isang E, Robinson LE, Parry SM, Morris PE, Neyra JA: **Safety and Feasibility of Physical Rehabilitation and Active Mobilization in Patients Requiring Continuous Renal Replacement Therapy: A Systematic Review**. *Crit Care Med* 2020, **48**(11):e1112-e1120.

22. Bento HA, Dummer D, Lohse BD, Noren C, Tonna JE: **Walking While Dialyzing: A Retrospective Observation of Early Mobility and Ambulation for Patients on Continuous Renal Replacement Therapy**. *Crit Care Explor* 2020, **2**(6):e0131.

23. Hermes C, Nydahl P, Blobner M, Dubb R, Filipovic S, Kaltwasser A, Ulm B, Schaller SJ: **Assessment of mobilization capacity in 10 different ICU scenarios by different professions**. *PLoS One* 2020, **15**(10):e0239853.

24. Ragland C, Ochoa L, Hartjes T: **Early mobilisation in intensive care during renal replacement therapy: A quality improvement project**. *Intensive Crit Care Nurs* 2019, **52**:22-27.

25. Dubb R, Nydahl P, Hermes C, Schwabbauer N, Toonstra A, Parker AM, Kaltwasser A, Needham DM: **Barriers and Strategies for Early Mobilization of Patients in Intensive Care Units**. *Ann Am Thorac Soc* 2016, **13**(5):724-730.

26. Eggers D, Hermes C, Esteve H, Nickoleit M, Filipovic S, Konig V, Riessen R, Kruger L, Nydahl P: **[Interprofessional curriculum for early mobilization : Developed by the nursing section of the DGIIN in close cooperation with the German early mobilization network]**. *Med Klin Intensivmed Notfmed* 2023, **118**(6):487-491.

27. Chen Y, Xu S: **Enhancing Quality Control in Continuous Renal Replacement Therapy Through ICU Specialist Nursing Care Management Program**. *Altern Ther Health Med* 2023, **30**(2):160-165.

28. Bai M, Zhou M, He L, Ma F, Li Y, Yu Y, Wang P, Li L, Jing R, Zhao L *et al*: **Citrate versus heparin anticoagulation for continuous renal replacement therapy: an updated meta-analysis of RCTs**. *Intensive Care Med* 2015, **41**(12):2098-2110.

29. Jacobs R, Verbrugghe W, Dams K, Roelant E, Couttenye MM, Devroey D, Jorens P: **Regional Citrate Anticoagulation in Continuous Renal Replacement Therapy: Is Metabolic Fear the Enemy of Logic? A Systematic Review and Meta-Analysis of Randomised Controlled Trials**. *Life (Basel)* 2023, **13**(5):1198.

30. Tsujimoto H, Tsujimoto Y, Nakata Y, Fujii T, Takahashi S, Akazawa M, Kataoka Y: **Pharmacological interventions for preventing clotting of extracorporeal circuits during continuous renal replacement therapy**. *Cochrane Database Syst Rev* 2020, **12**(12):Cd012467.

31. Liu C, Mao Z, Kang H, Hu J, Zhou F: **Regional citrate versus heparin anticoagulation for continuous renal replacement therapy in critically ill patients: a meta-analysis with trial sequential analysis of randomized controlled trials**. *Crit Care* 2016, **20**(1):144.

32. Zarbock A, Küllmar M, Kindgen-Milles D, Wempe C, Gerss J, Brandenburger T, Dimski T, Tyczynski B, Jahn M, Mülling N *et al*: **Effect of Regional Citrate Anticoagulation vs Systemic Heparin Anticoagulation During Continuous Kidney Replacement Therapy on Dialysis Filter Life Span and Mortality Among Critically Ill Patients With Acute Kidney Injury: a Randomized Clinical Trial**. *JAMA* 2020, **324**(16):1629‐1639.

33. Palevsky PM, Zhang JH, O'Connor TZ, Chertow GM, Crowley ST, Choudhury D, Finkel K, Kellum JA, Paganini E, Schein RMH *et al*: **Intensity of renal support in critically ill patients with acute kidney injury**. *New England Journal of Medicine* 2008, **359**(1):7-20.

34. Bellomo R, Cass A, Cole L, Finfer S, Gallagher M, Lo S, McArthur C, McGuinness S, Myburgh J, Norton R *et al*: **Intensity of continuous renal-replacement therapy in critically ill patients**. *N Engl J Med* 2009, **361**(17):1627-1638.

35. Abdul-Aziz MH, Alffenaar JC, Bassetti M, Bracht H, Dimopoulos G, Marriott D, Neely MN, Paiva JA, Pea F, Sjovall F *et al*: **Antimicrobial therapeutic drug monitoring in critically ill adult patients: a Position Paper()**. *Intensive Care Med* 2020, **46**(6):1127-1153.

36. Guilhaumou R, Benaboud S, Bennis Y, Dahyot-Fizelier C, Dailly E, Gandia P, Goutelle S, Lefeuvre S, Mongardon N, Roger C *et al*: **Optimization of the treatment with beta-lactam antibiotics in critically ill patients-guidelines from the French Society of Pharmacology and Therapeutics (Societe Francaise de Pharmacologie et Therapeutique-SFPT) and the French Society of Anaesthesia and Intensive Care Medicine (Societe Francaise d'Anesthesie et Reanimation-SFAR)**. *Crit Care* 2019, **23**(1):104.

37. Hong LT, Downes KJ, FakhriRavari A, Abdul-Mutakabbir JC, Kuti JL, Jorgensen S, Young DC, Alshaer MH, Bassetti M, Bonomo RA *et al*: **International consensus recommendations for the use of prolonged-infusion beta-lactam antibiotics: Endorsed by the American College of Clinical Pharmacy, British Society for Antimicrobial Chemotherapy, Cystic Fibrosis Foundation, European Society of Clinical Microbiology and Infectious Diseases, Infectious Diseases Society of America, Society of Critical Care Medicine, and Society of Infectious Diseases Pharmacists**. *Pharmacotherapy* 2023, **43**(8):740-777.

38. Matusik E, Boidin C, Friggeri A, Richard JC, Bitker L, Roberts JA, Goutelle S: **Therapeutic Drug Monitoring of Antibiotic Drugs in Patients Receiving Continuous Renal Replacement Therapy or Intermittent Hemodialysis: A Critical Review**. *Ther Drug Monit* 2022b, **44**(1):86-102.

39. Richter DC, Frey O, Röhr A, Roberts JA, Köberer A, Fuchs T, Papadimas N, Heinzel-Gutenbrunner M, Brenner T, Lichtenstern C *et al*: **Therapeutic drug monitoring-guided continuous infusion of piperacillin/tazobactam significantly improves pharmacokinetic target attainment in critically ill patients: a retrospective analysis of four years of clinical experience**. *Infection* 2019, **47**(6):1001-1011.

40. Hagel S, Bach F, Brenner T, Bracht H, Brinkmann A, Annecke T, Hohn A, Weigand M, Michels G, Kluge S *et al*: **Effect of therapeutic drug monitoring-based dose optimization of piperacillin/tazobactam on sepsis-related organ dysfunction in patients with sepsis: a randomized controlled trial**. *Intensive care medicine* 2022, **48**(3):311‐321.

41. Ewoldt TMJ, Abdulla A, Rietdijk WJR, Muller AE, de Winter BCM, Hunfeld NGM, Purmer IM, van Vliet P, Wils EJ, Haringman J *et al*: **Model-informed precision dosing of beta-lactam antibiotics and ciprofloxacin in critically ill patients: a multicentre randomised clinical trial**. *Intensive Care Med* 2022, **48**(12):1760-1771.

42. Liebchen U, Briegel J, Brinkmann A, Frey O, Wicha SG: **Individualised dosing of antibiotics in ICU patients: timing, target and model selection matter**. *Intensive Care Med* 2023, **49**(4):475-476.

43. Luxton T, King N, Walti C, Jeuken L, Sandoe J: **A systematic review of the effect of therapeutic drug monitoring on patient health outcomes during treatment with penicillins**. *J Antimicrob Chemother* 2022, **77**(6):1532-1541.

44. Crass RL, Cojutti PG, Pai MP, Pea F: **Reappraisal of Linezolid Dosing in Renal Impairment To Improve Safety**. *Antimicrob Agents Chemother* 2019, **63**(8):e00605-00619.

45. Rao GG, Konicki R, Cattaneo D, Alffenaar JW, Marriott DJE, Neely M, Committee IAS: **Therapeutic Drug Monitoring Can Improve Linezolid Dosing Regimens in Current Clinical Practice: A Review of Linezolid Pharmacokinetics and Pharmacodynamics**. *Ther Drug Monit* 2020, **42**(1):83-92.

46. Decker BS, Goldfarb DS, Dargan PI, Friesen M, Gosselin S, Hoffman RS, Lavergne V, Nolin TD, Ghannoum M: **Extracorporeal Treatment for Lithium Poisoning: Systematic Review and Recommendations from the EXTRIP Workgroup**. *Clin J Am Soc Nephrol* 2015, **10**(5):875-887.

47. Lavonas EJ, Buchanan, J.: **Hemodialysis for lithium poisoning**. *Cochrane Database Syst Rev* 2015, **2015**(9):Cd007951.

48. Ott M, Stegmayr B, Salander Renberg E, Werneke U: **Lithium intoxication: Incidence, clinical course and renal function - a population-based retrospective cohort study**. *J Psychopharmacol* 2016, **30**(10):1008-1019.

49. Eyer F, Pfab R, Felgenhauer N, Lutz J, Heemann U, Steimer W, Zondler S, Fichtl B, Zilker T: **Lithium poisoning: pharmacokinetics and clearance during different therapeutic measures**. *J Clin Psychopharmacol* 2006, **26**(3):325-330.

50. Meertens JH, Jagernath DR, Eleveld DJ, Zijlstra JG, Franssen CF: **Haemodialysis followed by continuous veno-venous haemodiafiltration in lithium intoxication; a model and a case**. *Eur J Intern Med* 2009, **20**(3):e70-73.

51. Schmidt JJ, Lorenzen J, Chatzikyrkou C, Lichtinghagen R, Kielstein JT: **Total collected dialysate lithium concentration after successful dialysis treatment in case of intoxication**. *BMC Pharmacol Toxicol* 2014, **15**:49.

52. Baeg SI, Jeon J, Yoo H, Na SJ, Kim K, Chung CR, Yang JH, Jeon K, Lee JE, Huh W *et al*: **A Scoring Model with Simple Clinical Parameters to Predict Successful Discontinuation of Continuous Renal Replacement Therapy**. *Blood Purif* 2021, **50**(6):779-789.

53. Itenov TS, Berthelsen RE, Jensen JU, Gerds TA, Pedersen LM, Strange D, Thormar K, Loken J, Andersen MH, Tousi H *et al*: **Predicting recovery from acute kidney injury in critically ill patients: development and validation of a prediction model**. *Crit Care Resusc* 2018, **20**(1):54-60.

54. Zhang Z, Xu X, Ni H, Jin N: **Serum cystatin C is associated with renal function recovery in critically ill patients undergoing continuous renal replacement therapy**. *Nephron Clin Pract* 2012, **122**(3-4):86-92.

55. Yang T, Sun S, Zhao Y, Liu Q, Han M, Lin L, Su B, Huang S, Yang L: **Biomarkers upon discontinuation of renal replacement therapy predict 60-day survival and renal recovery in critically ill patients with acute kidney injury**. *Hemodialysis International* 2018, **22**(1):56-65.

56. Vijayan A, Abdel-Rahman EM, Liu KD, Goldstein SL, Agarwal A, Okusa MD, Cerda J, Committee ANS: **Recovery after Critical Illness and Acute Kidney Injury**. *Clin J Am Soc Nephrol* 2021, **16**(10):1601-1609.

57. Abdala PM, Swanson EA, Hutchens MP: **Meta-analysis of AKI to CKD transition in perioperative patients**. *Perioper Med (Lond)* 2021, **10**(1):24.

58. Kashani K, Rosner MH, Haase M, Lewington AJP, O'Donoghue DJ, Wilson FP, Nadim MK, Silver SA, Zarbock A, Ostermann M *et al*: **Quality Improvement Goals for Acute Kidney Injury**. *Clin J Am Soc Nephrol* 2019, **14**(6):941-953.
